# Supplementary figures and images for: A MET-PTPRK kinase-phosphatase rheostat controls ZNRF3 and Wnt signaling
Source: eLife. 2021 Sep 30;10:e70885. doi: 10.7554/eLife.70885 (PMC8516413; doi:10.7554/eLife.70885)

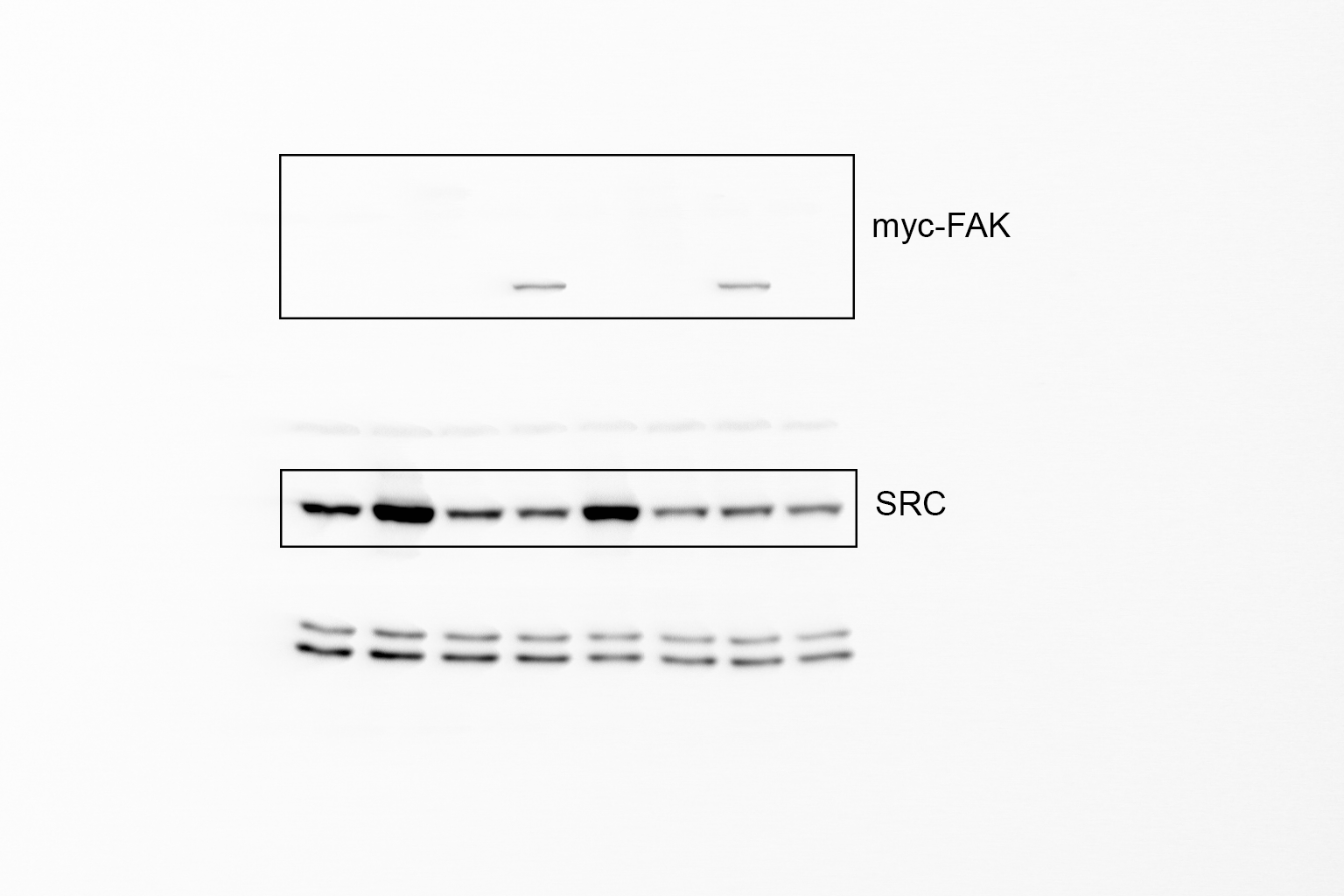

Supplement: Figure 1—source data 1. [file elife-70885-fig1-data1.zip › Figure 1-source data 1/Figure 1D_Input (FAK_SRC).tif]

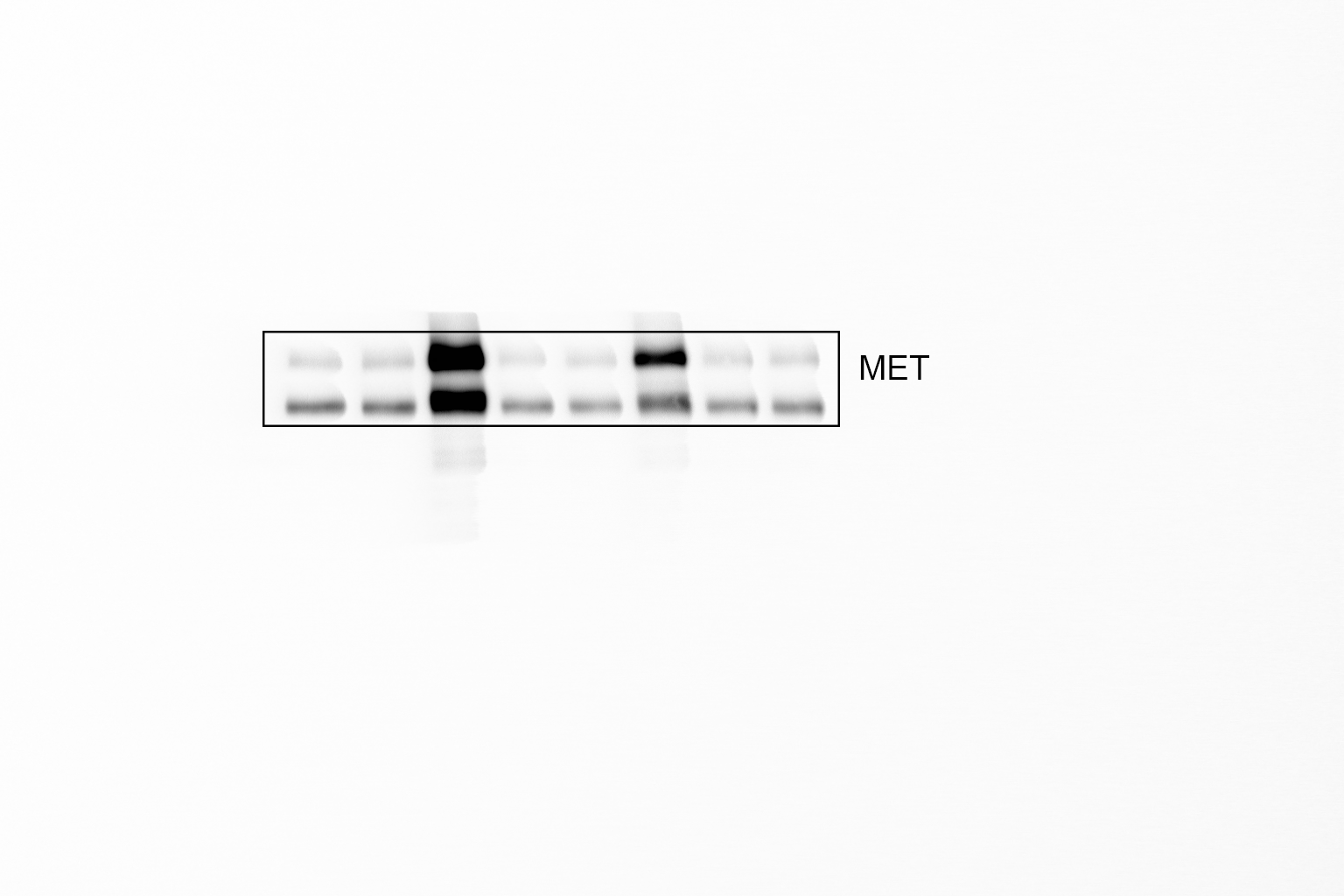

Supplement: Figure 1—source data 1. [file elife-70885-fig1-data1.zip › Figure 1-source data 1/Figure 1D_Input (MET).tif]

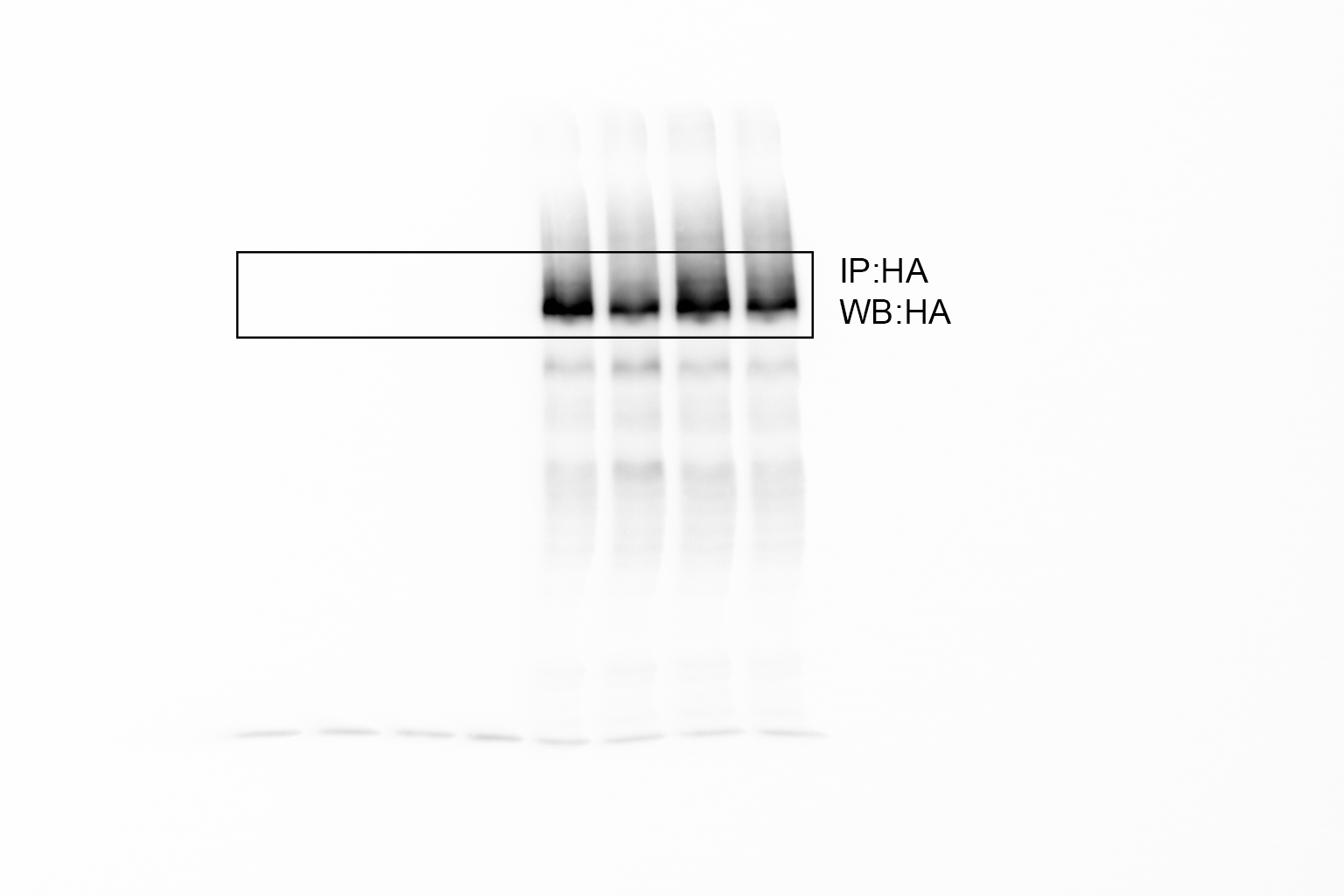

Supplement: Figure 1—source data 1. [file elife-70885-fig1-data1.zip › Figure 1-source data 1/Figure 1D_IP(HA).tif]

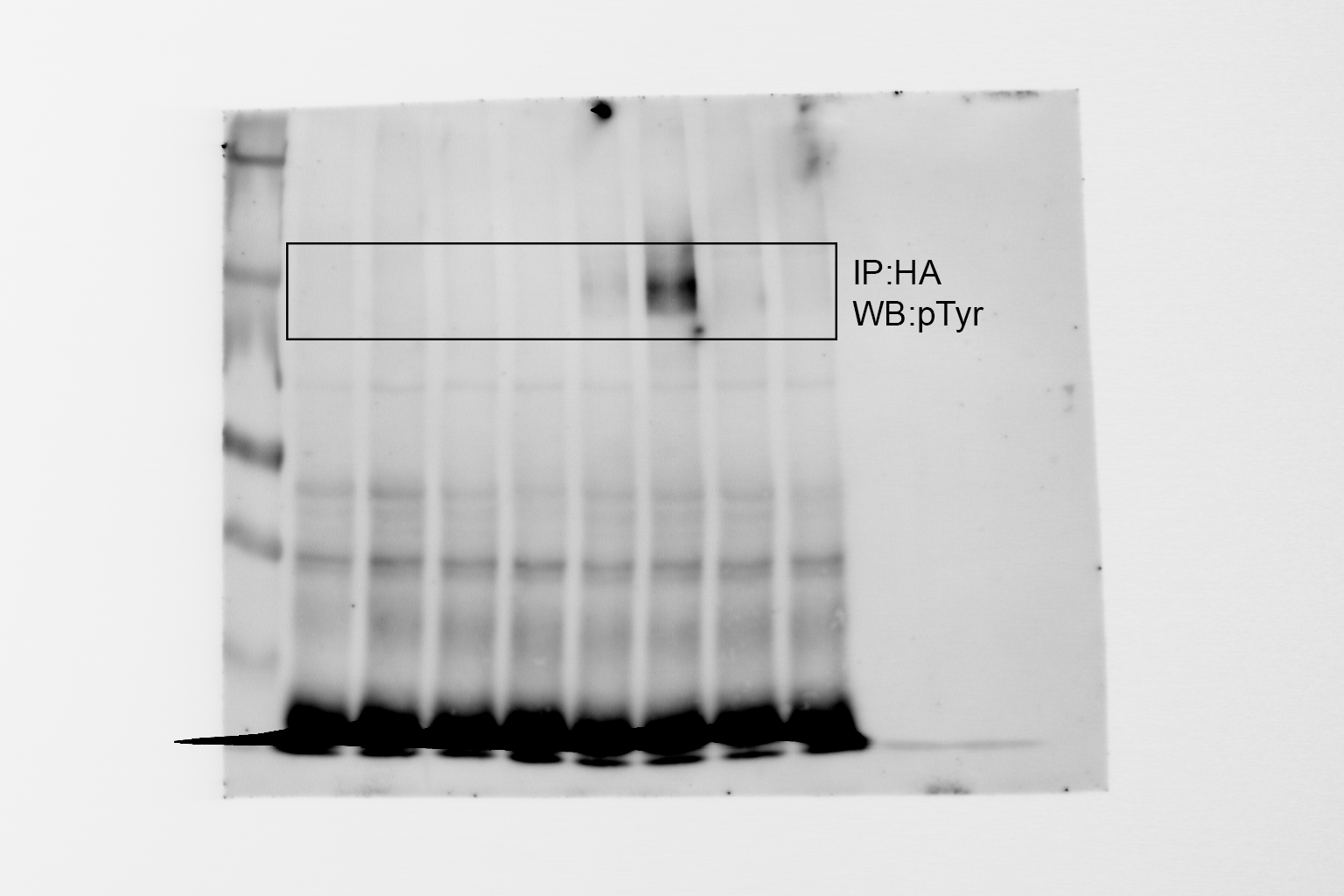

Supplement: Figure 1—source data 1. [file elife-70885-fig1-data1.zip › Figure 1-source data 1/Figure 1D_IP(pTyr).tif]

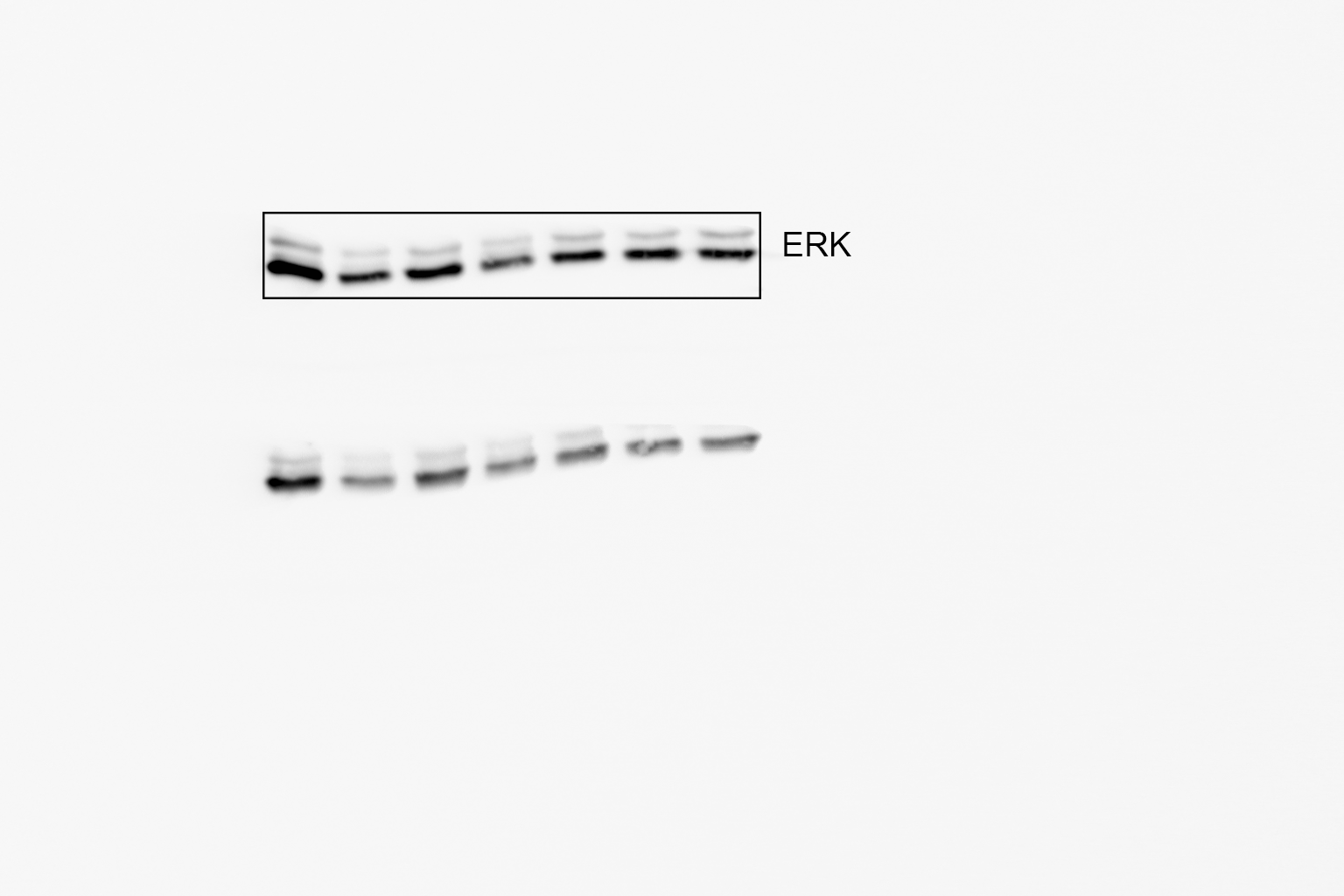

Supplement: Figure 2—source data 1. [file elife-70885-fig2-data1.zip › Figure 2-source data1/Figure 2A/Figure 2A (ERK)tif.tif]

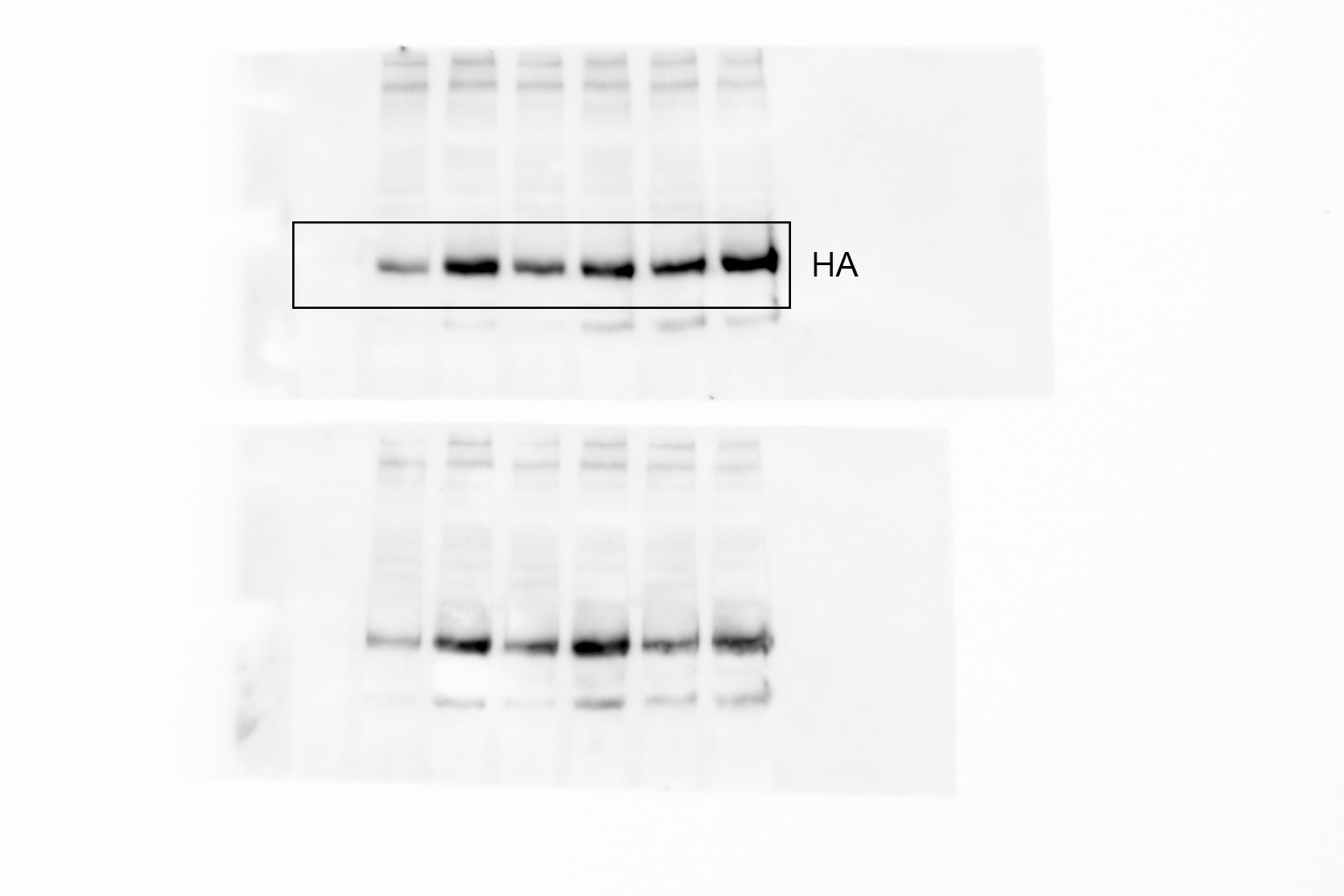

Supplement: Figure 2—source data 1. [file elife-70885-fig2-data1.zip › Figure 2-source data1/Figure 2A/Figure 2A (HA)tif.tif]

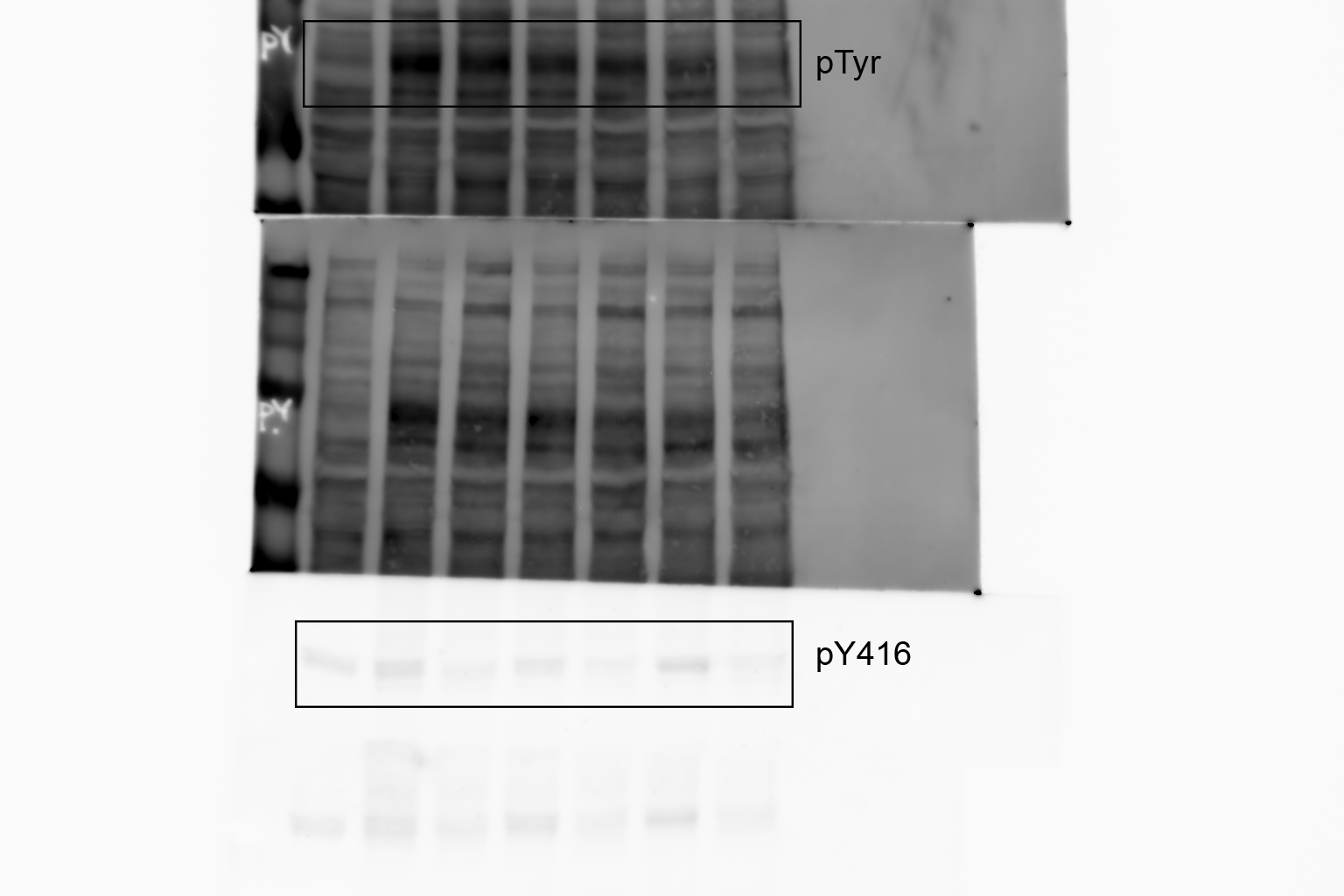

Supplement: Figure 2—source data 1. [file elife-70885-fig2-data1.zip › Figure 2-source data1/Figure 2A/Figure 2A (pTyr_pY416)tif.tif]

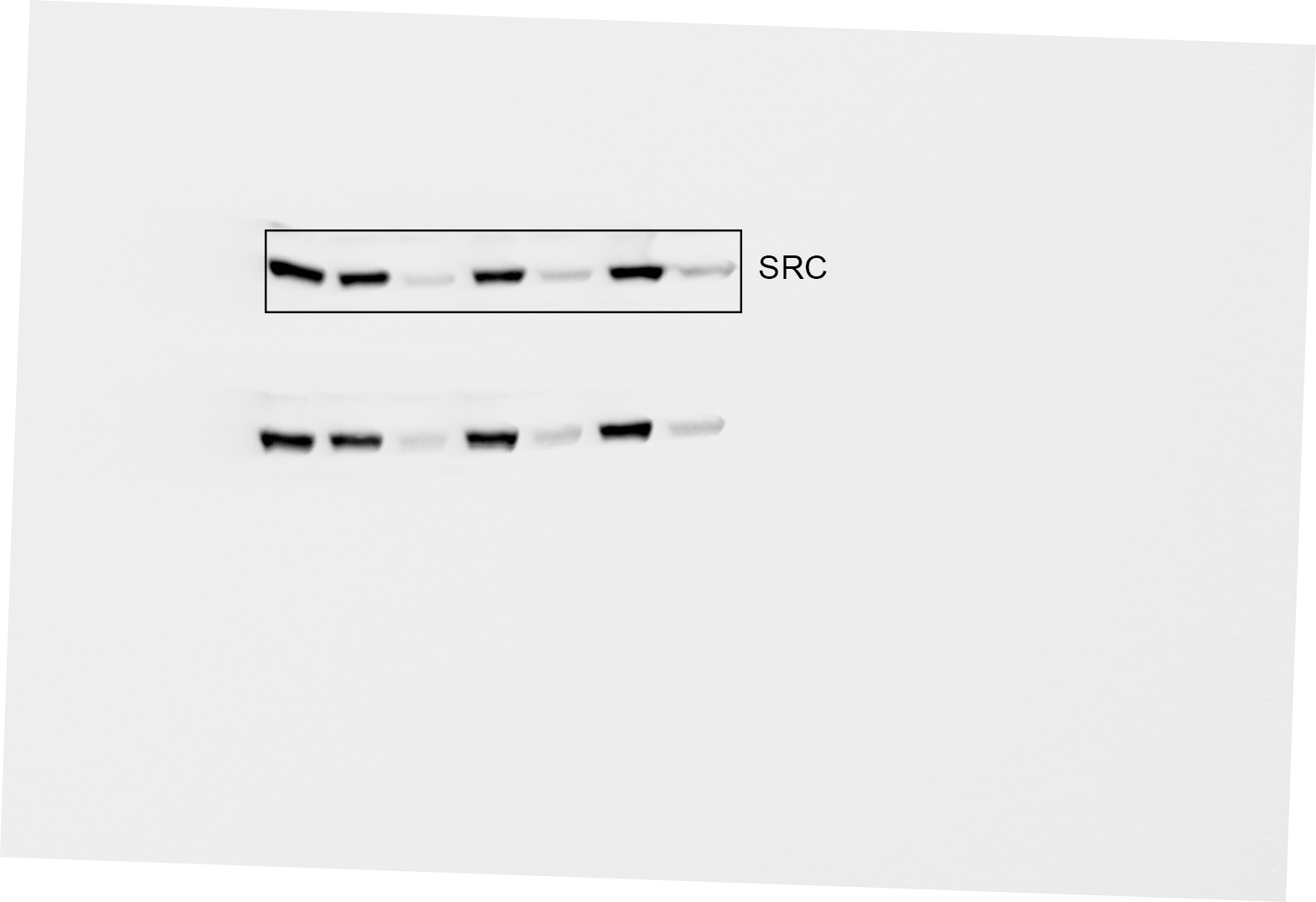

Supplement: Figure 2—source data 1. [file elife-70885-fig2-data1.zip › Figure 2-source data1/Figure 2A/Figure 2A (SRC)tif.tif]

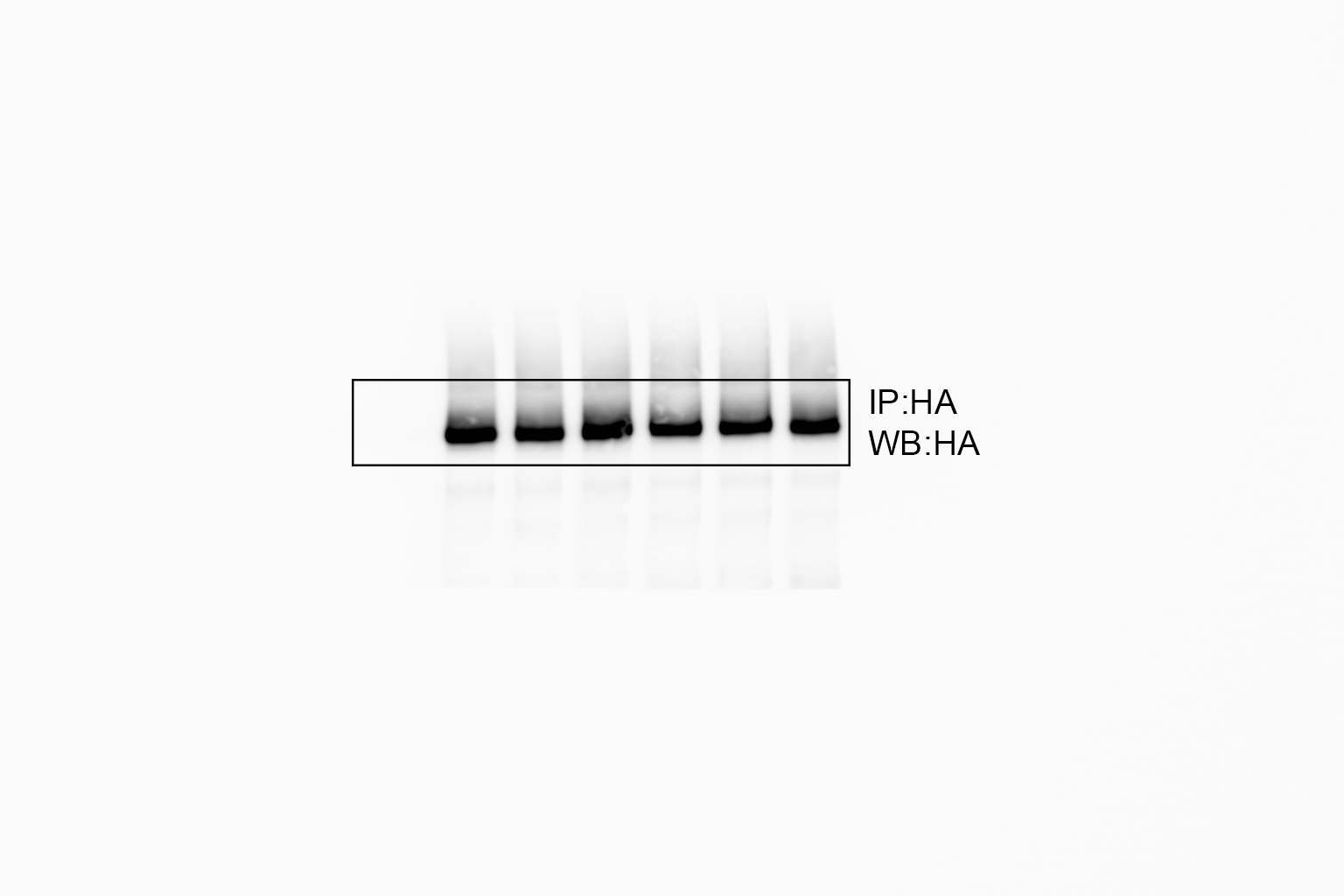

Supplement: Figure 2—source data 1. [file elife-70885-fig2-data1.zip › Figure 2-source data1/Figure 2B/Figure 2B (HA).tif]

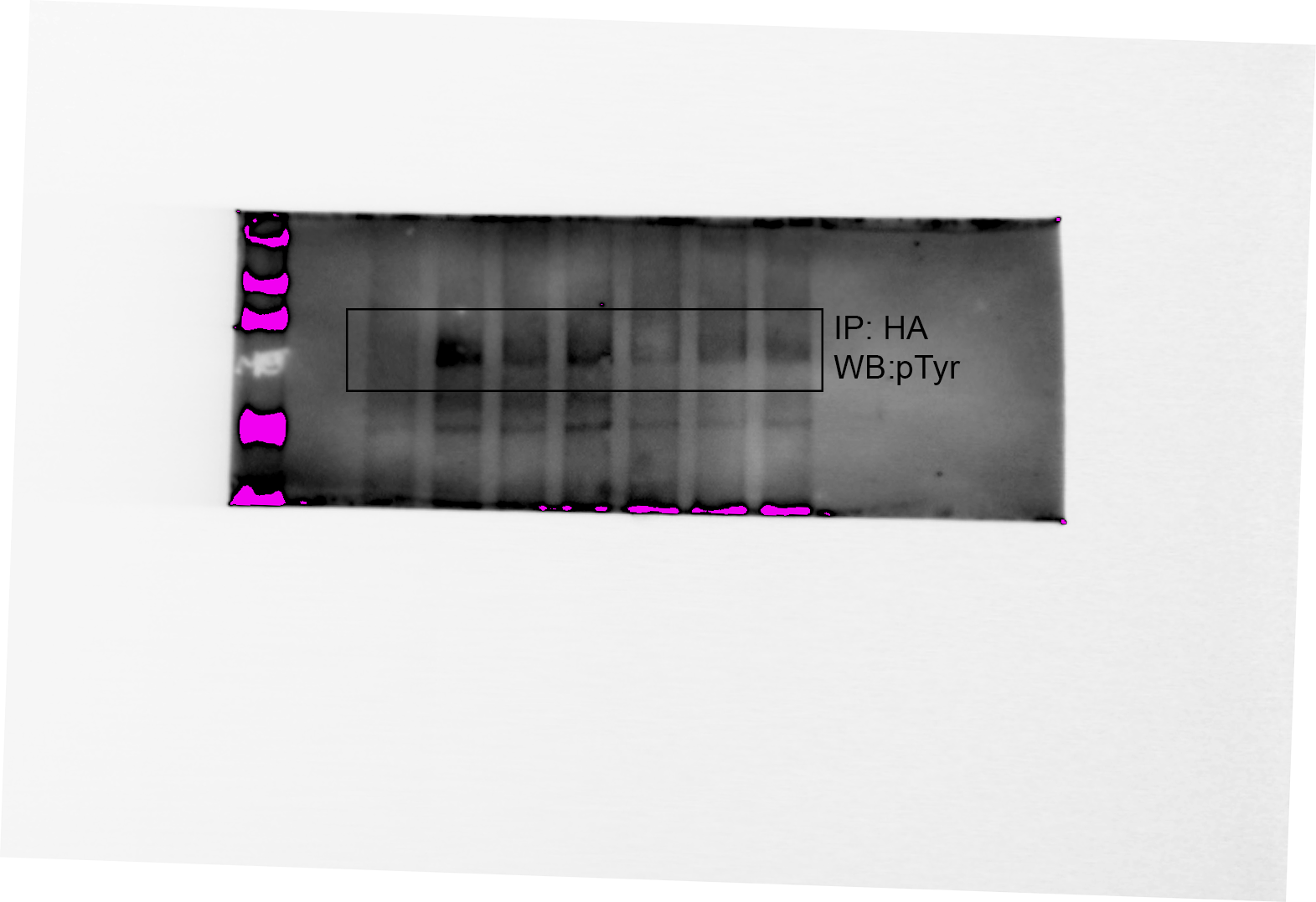

Supplement: Figure 2—source data 1. [file elife-70885-fig2-data1.zip › Figure 2-source data1/Figure 2B/Figure 2B (pTyr).tif]

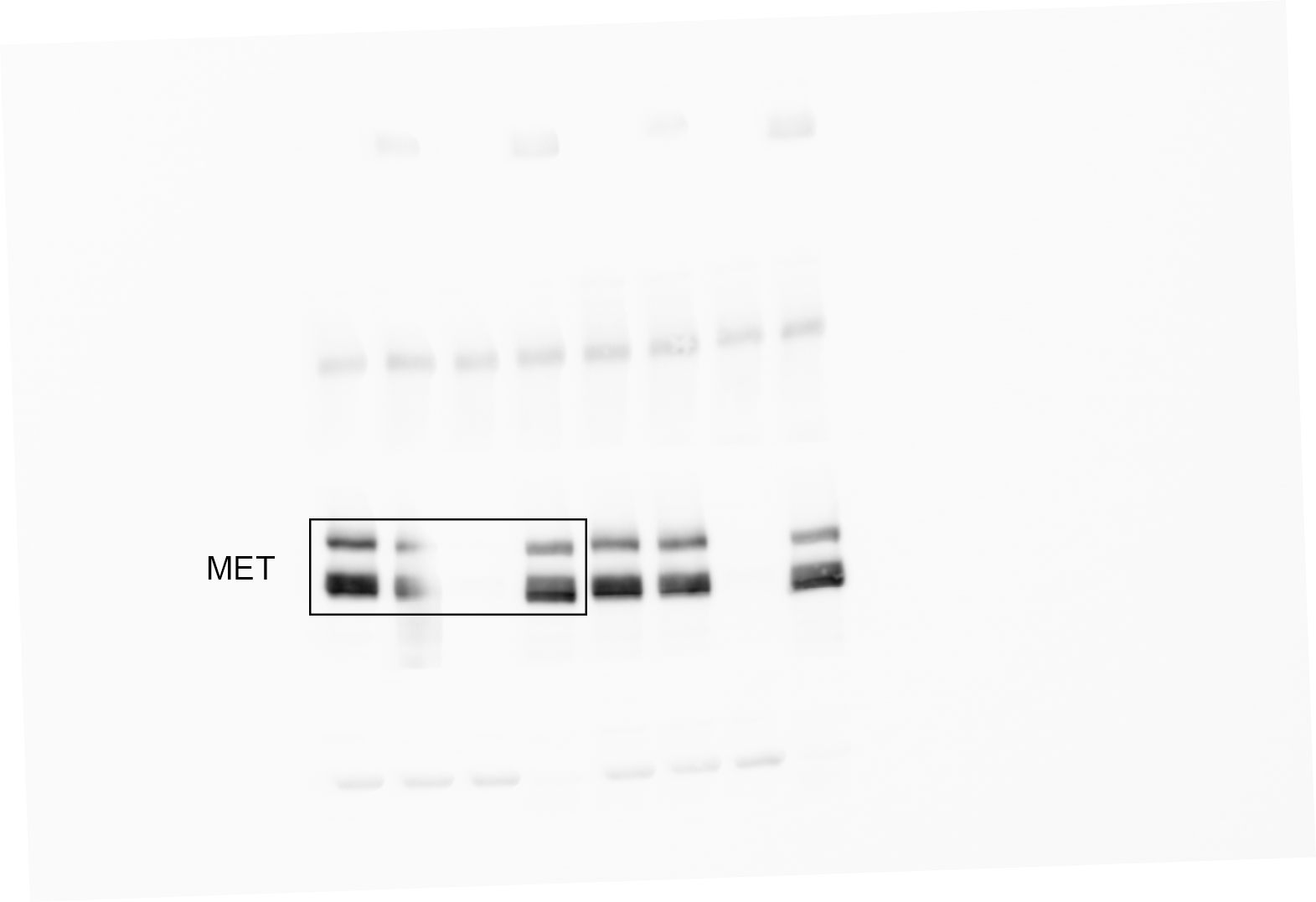

Supplement: Figure 2—source data 1. [file elife-70885-fig2-data1.zip › Figure 2-source data1/Figure 2D/Figure 2D Input(MET).tif]

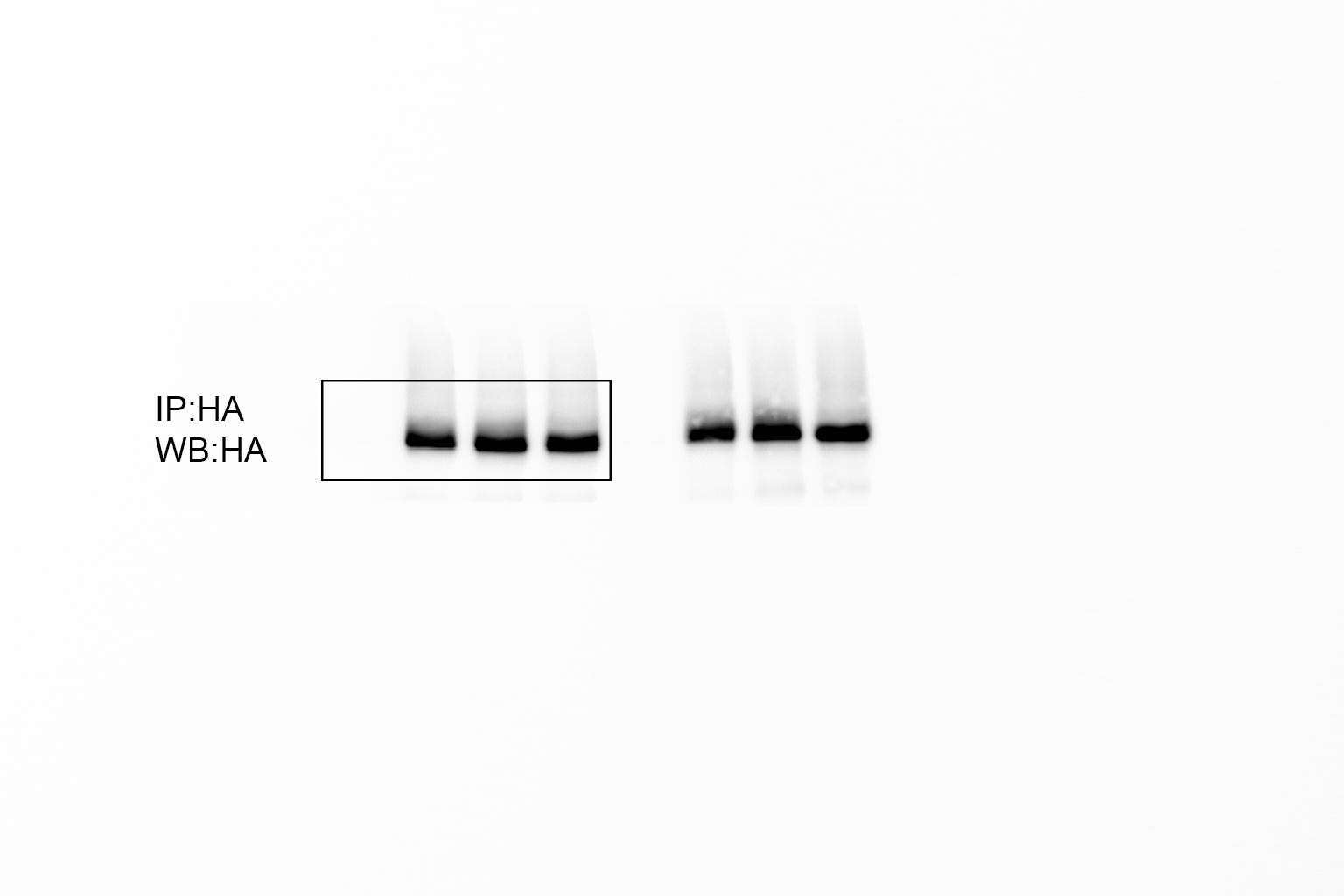

Supplement: Figure 2—source data 1. [file elife-70885-fig2-data1.zip › Figure 2-source data1/Figure 2D/Figure 2D IP(HA).tif]

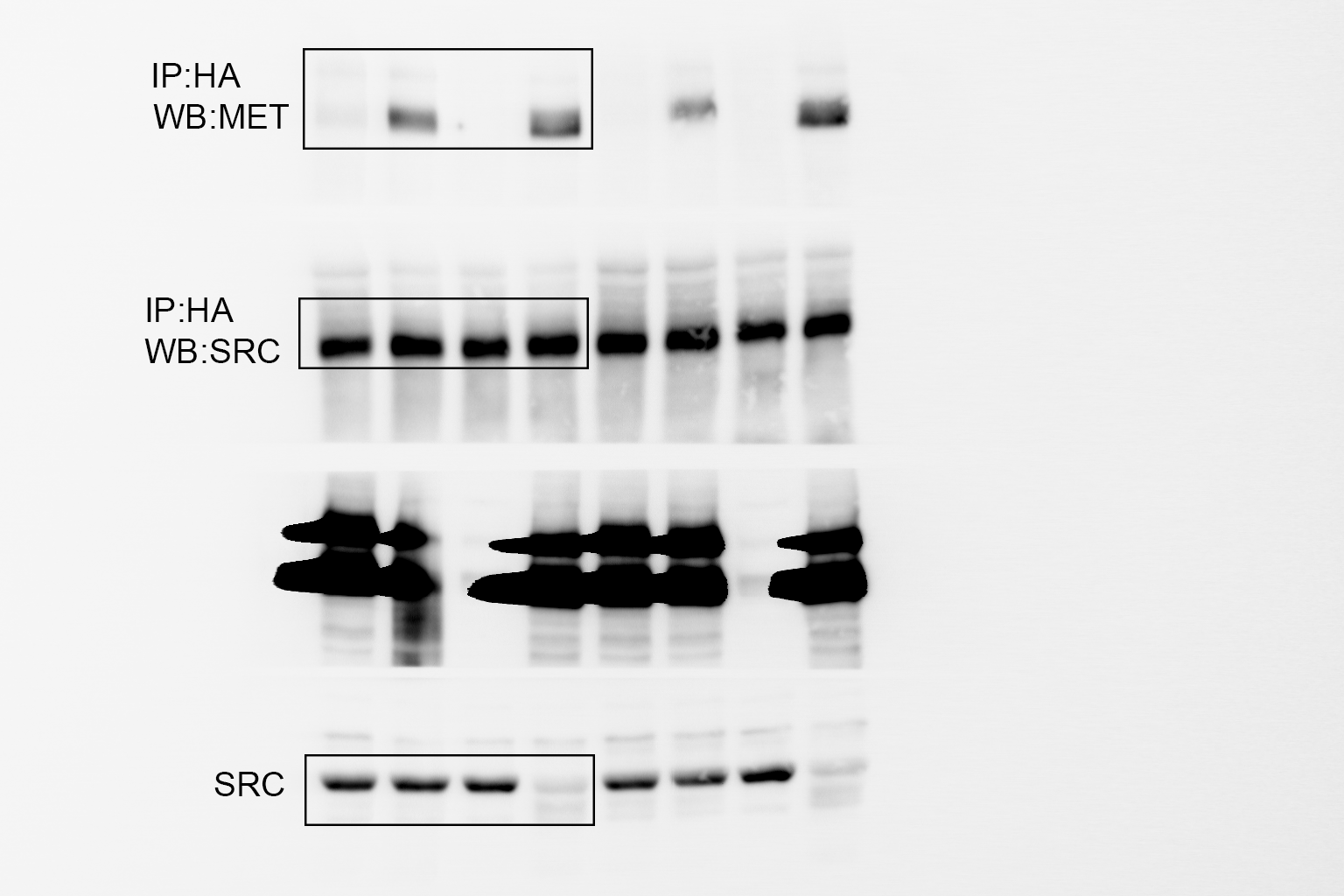

Supplement: Figure 2—source data 1. [file elife-70885-fig2-data1.zip › Figure 2-source data1/Figure 2D/Figure 2D IP(MET_SRC)_Input(SRC).tif]

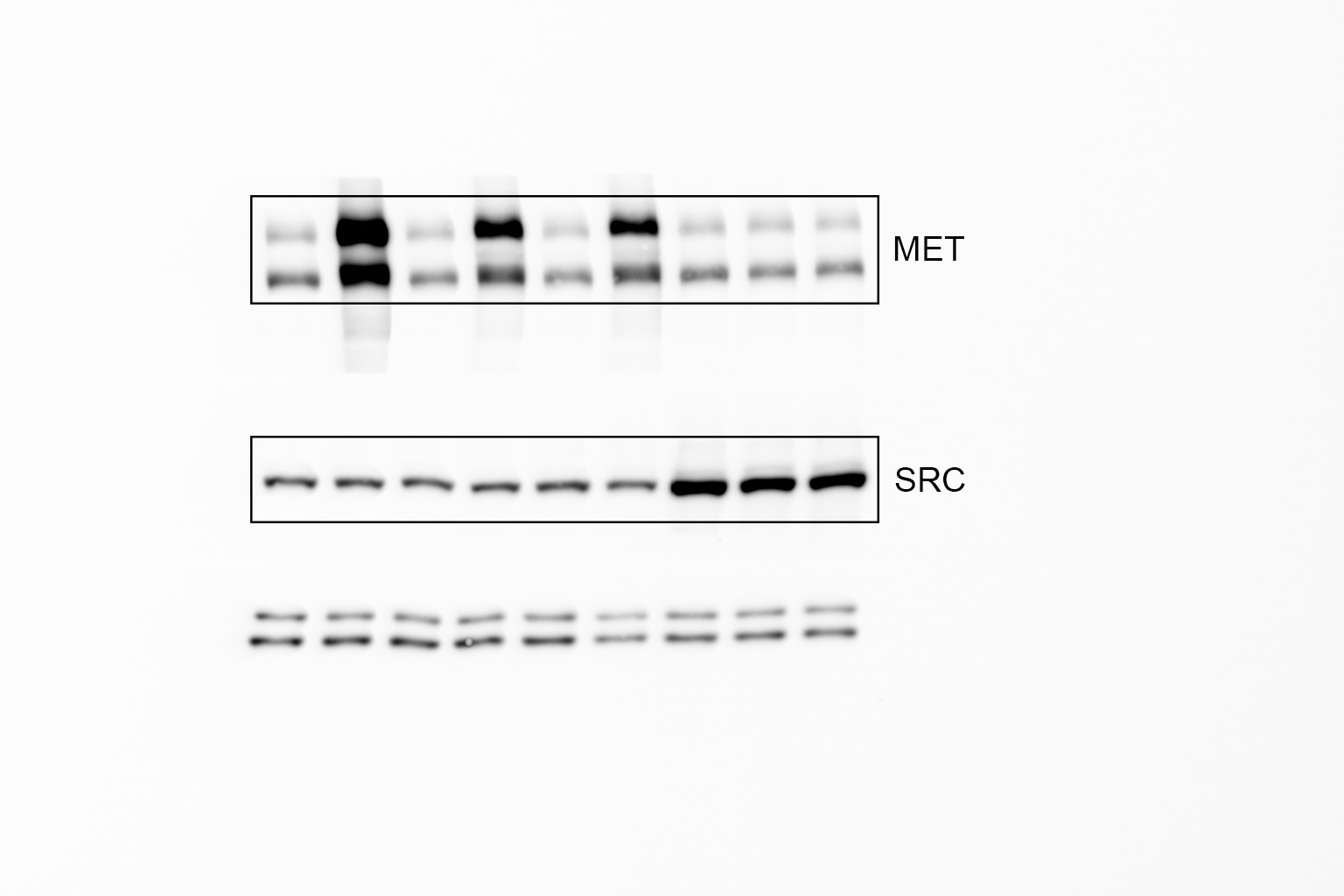

Supplement: Figure 2—source data 1. [file elife-70885-fig2-data1.zip › Figure 2-source data1/Figure 2E/Figure 2E Input(MET_SRC).tif]

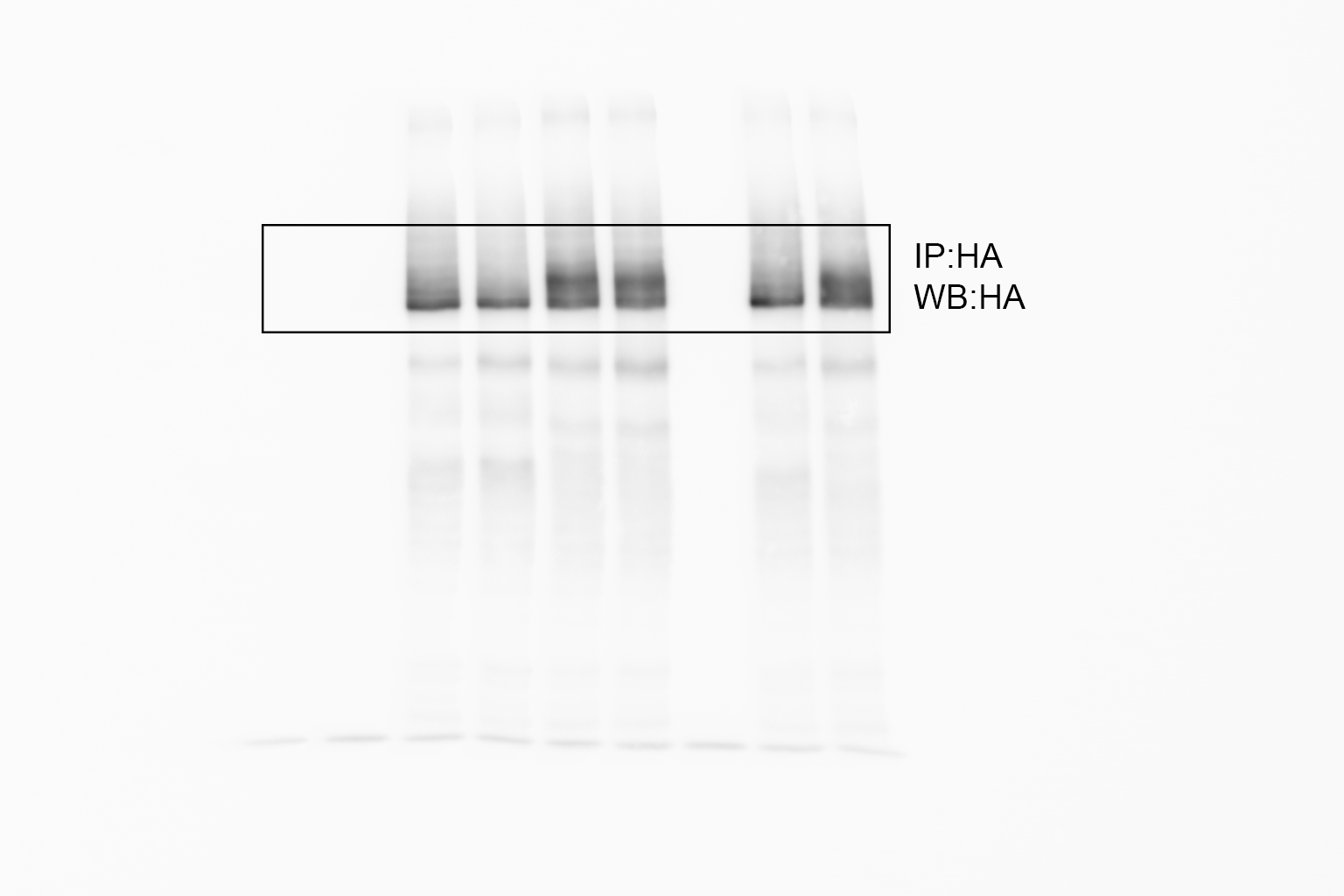

Supplement: Figure 2—source data 1. [file elife-70885-fig2-data1.zip › Figure 2-source data1/Figure 2E/Figure 2E IP(HA).tif]

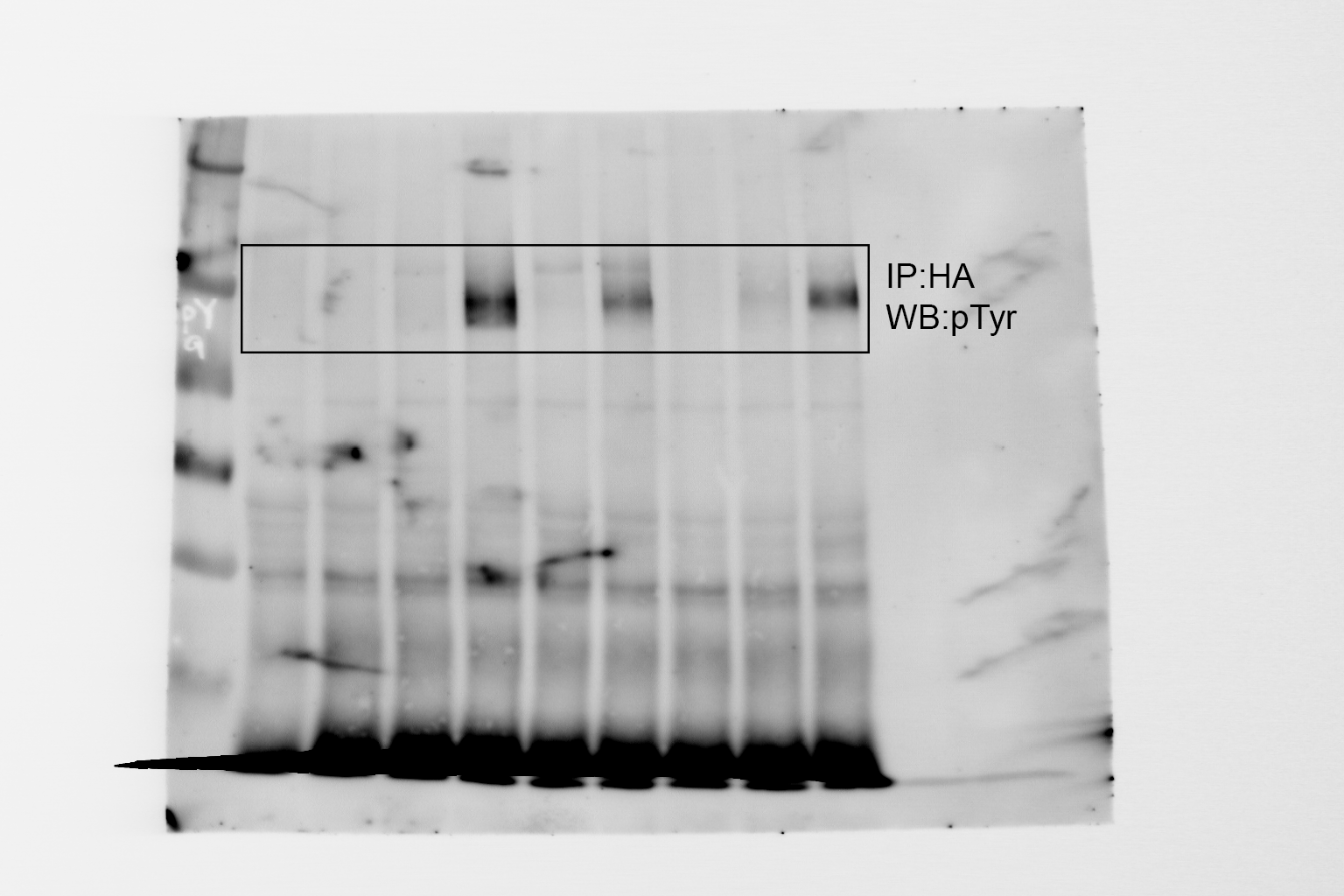

Supplement: Figure 2—source data 1. [file elife-70885-fig2-data1.zip › Figure 2-source data1/Figure 2E/Figure 2E IP(pTyr).tif]

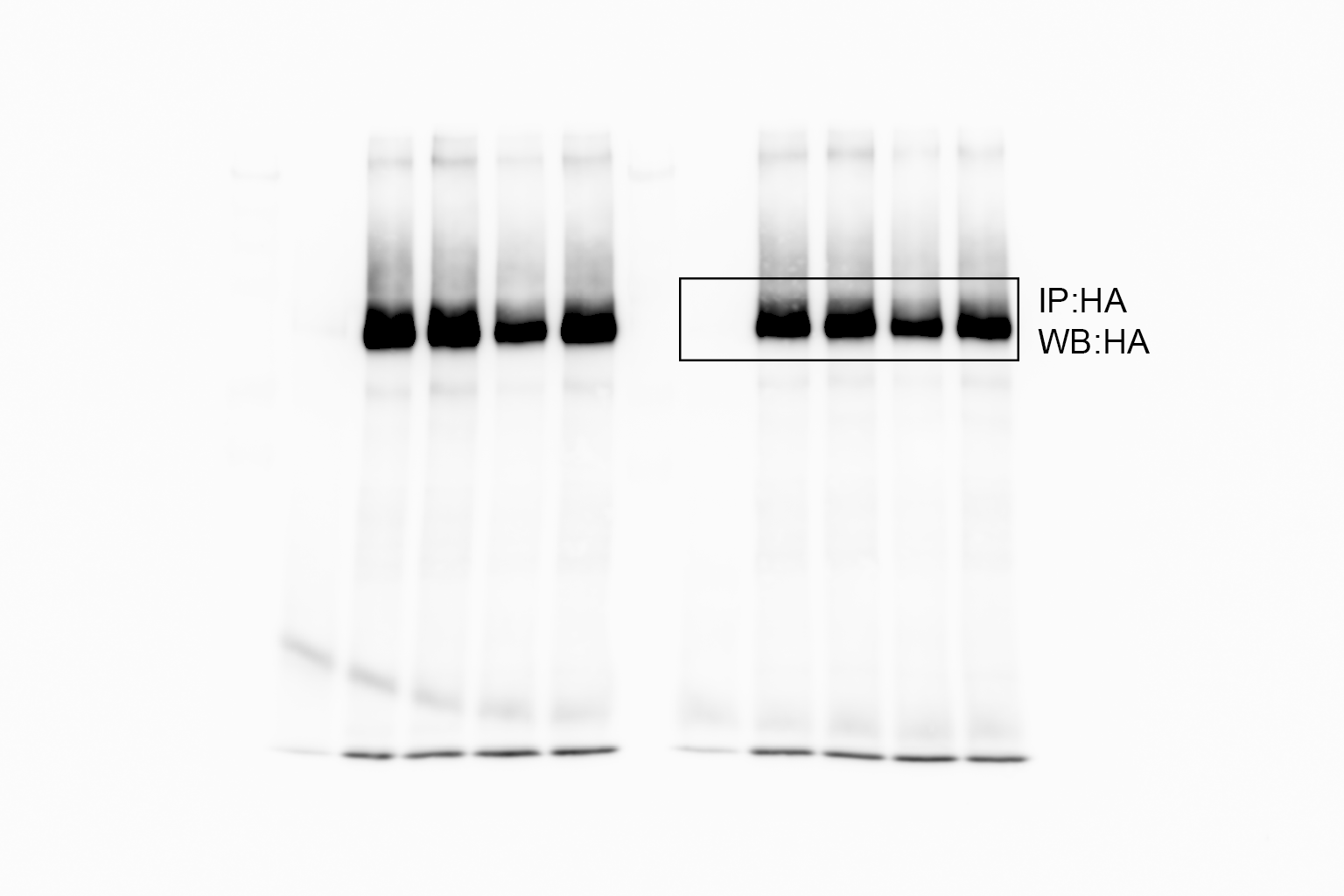

Supplement: Figure 2—source data 1. [file elife-70885-fig2-data1.zip › Figure 2-source data1/Figure 2F/Figure 2F IP(HA).tif]

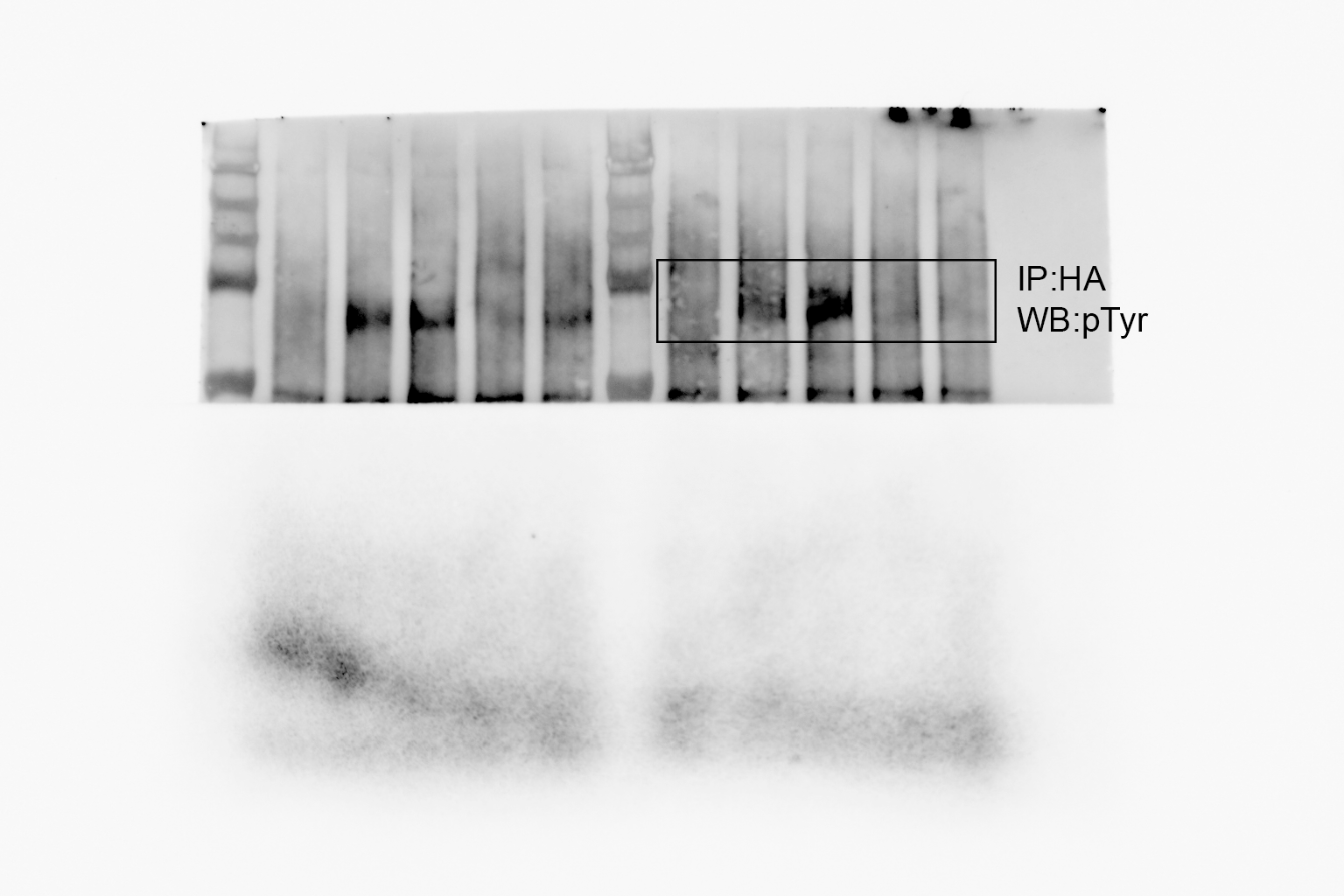

Supplement: Figure 2—source data 1. [file elife-70885-fig2-data1.zip › Figure 2-source data1/Figure 2F/Figure 2F IP(pTyr).tif]

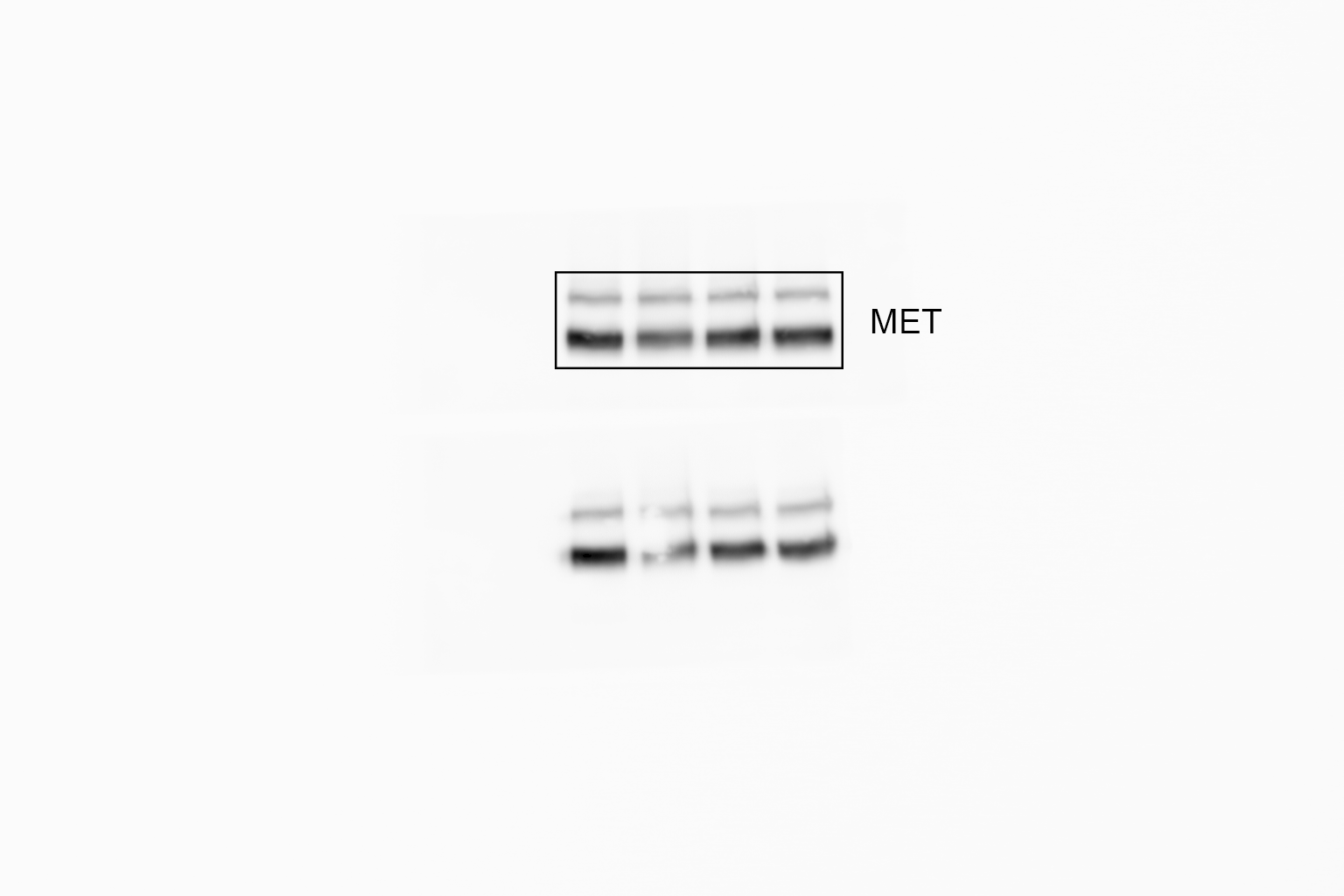

Supplement: Figure 3—source data 1. [file elife-70885-fig3-data1.zip › Figure 3-source data 1/Figure 3A/Figure 3A Input (MET).tif]

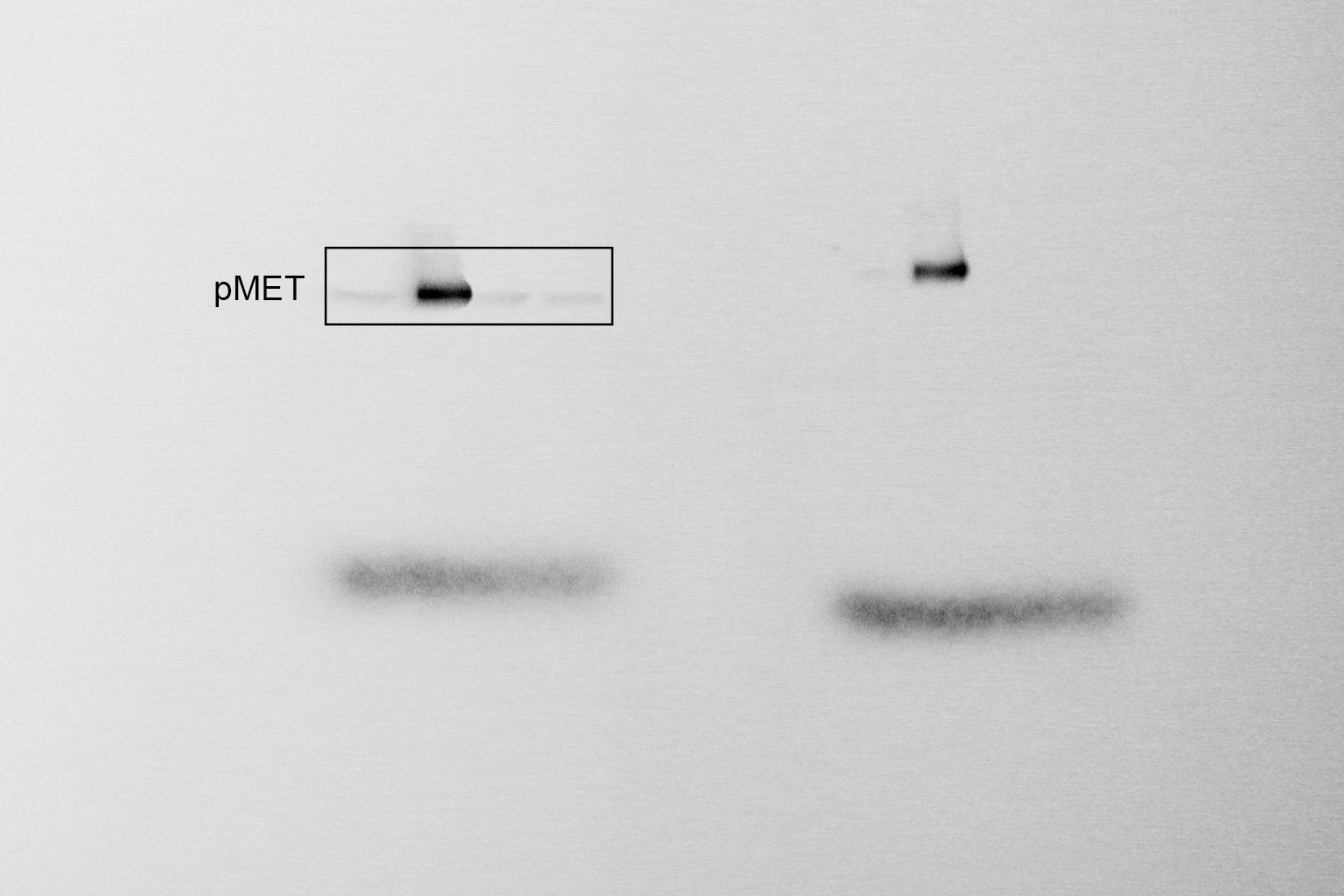

Supplement: Figure 3—source data 1. [file elife-70885-fig3-data1.zip › Figure 3-source data 1/Figure 3A/Figure 3A Input (pMET).tif]

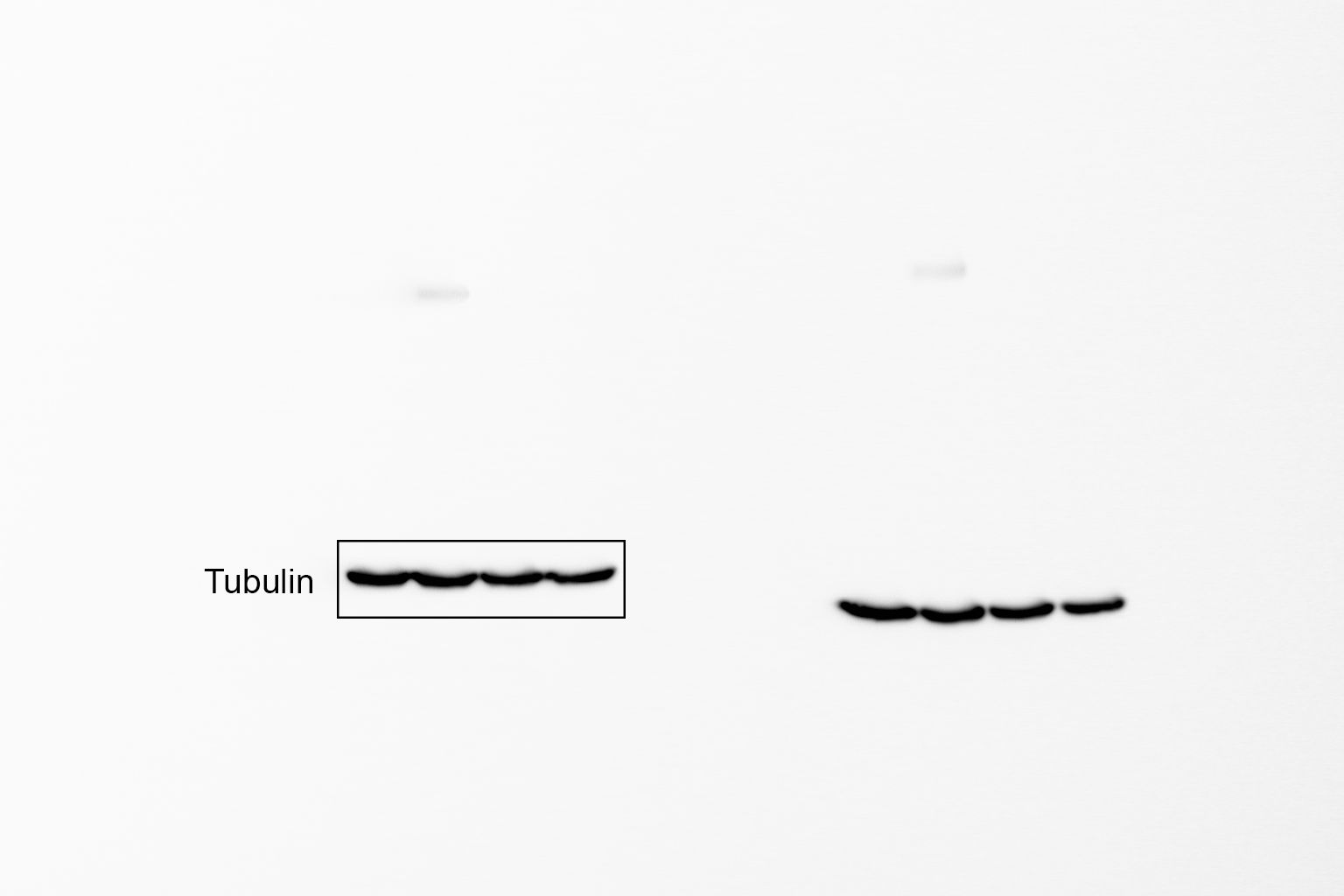

Supplement: Figure 3—source data 1. [file elife-70885-fig3-data1.zip › Figure 3-source data 1/Figure 3A/Figure 3A Input (Tub).tif]

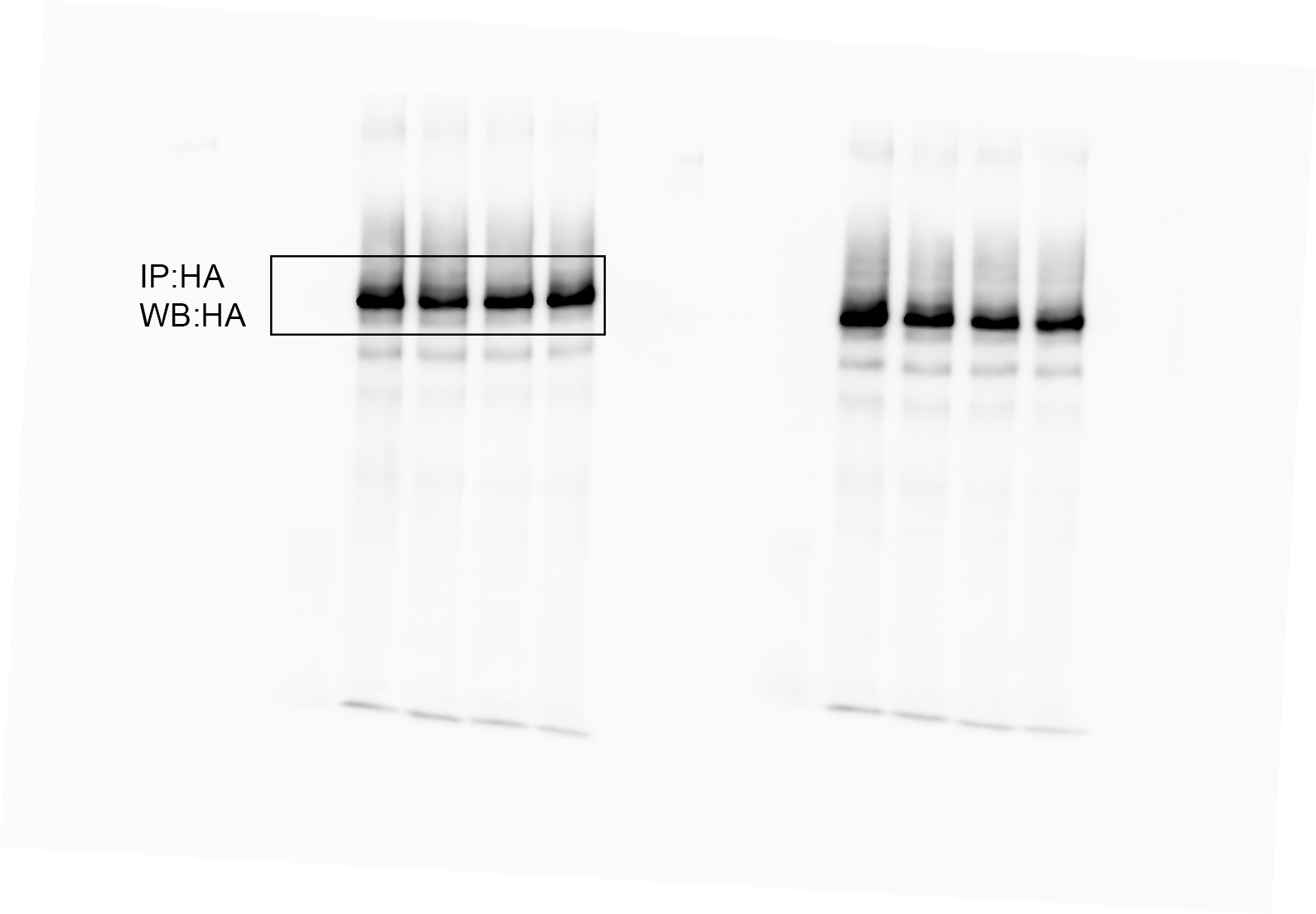

Supplement: Figure 3—source data 1. [file elife-70885-fig3-data1.zip › Figure 3-source data 1/Figure 3A/Figure 3A IP (HA).tif]

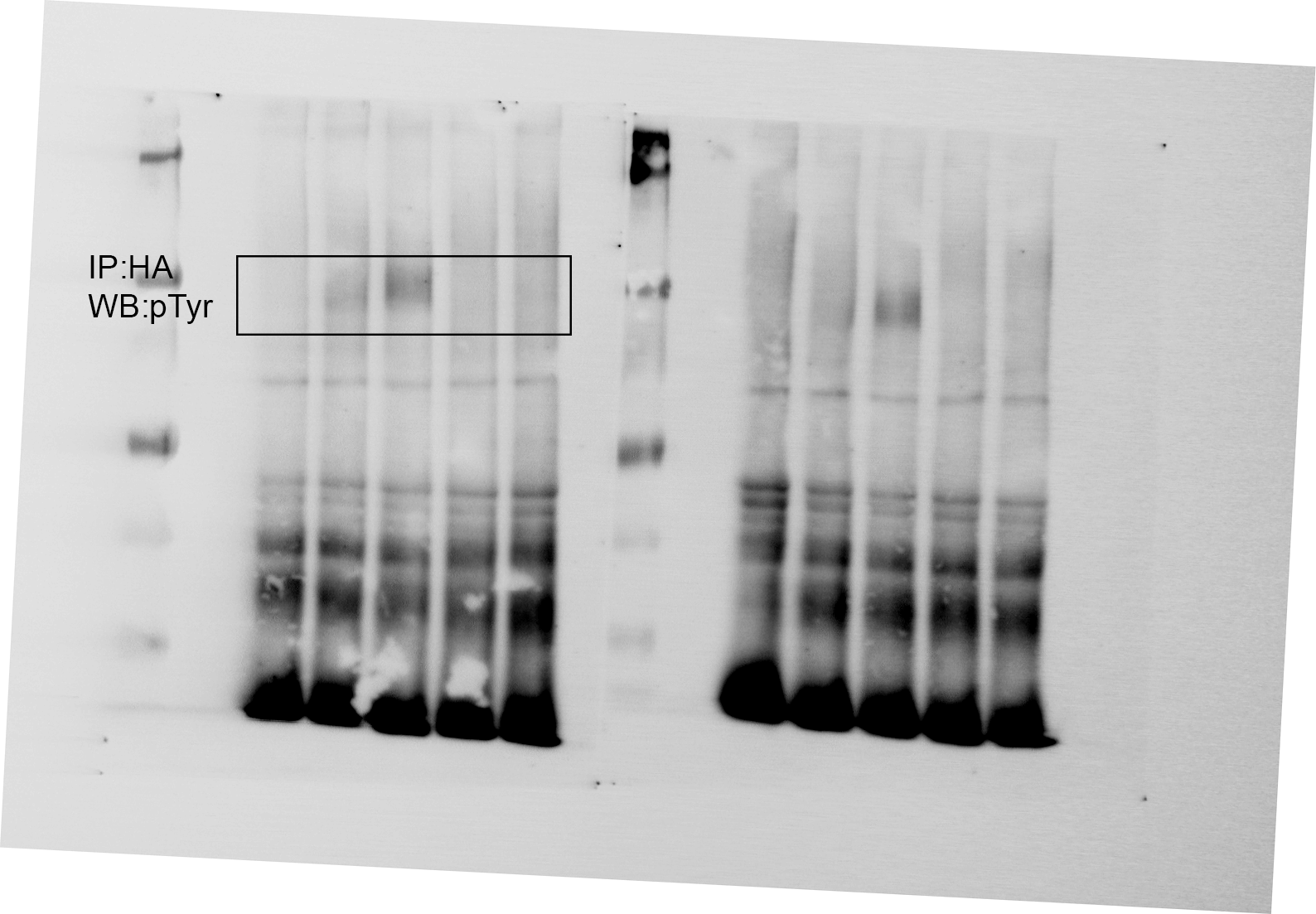

Supplement: Figure 3—source data 1. [file elife-70885-fig3-data1.zip › Figure 3-source data 1/Figure 3A/Figure 3A IP (pTyr).tif]

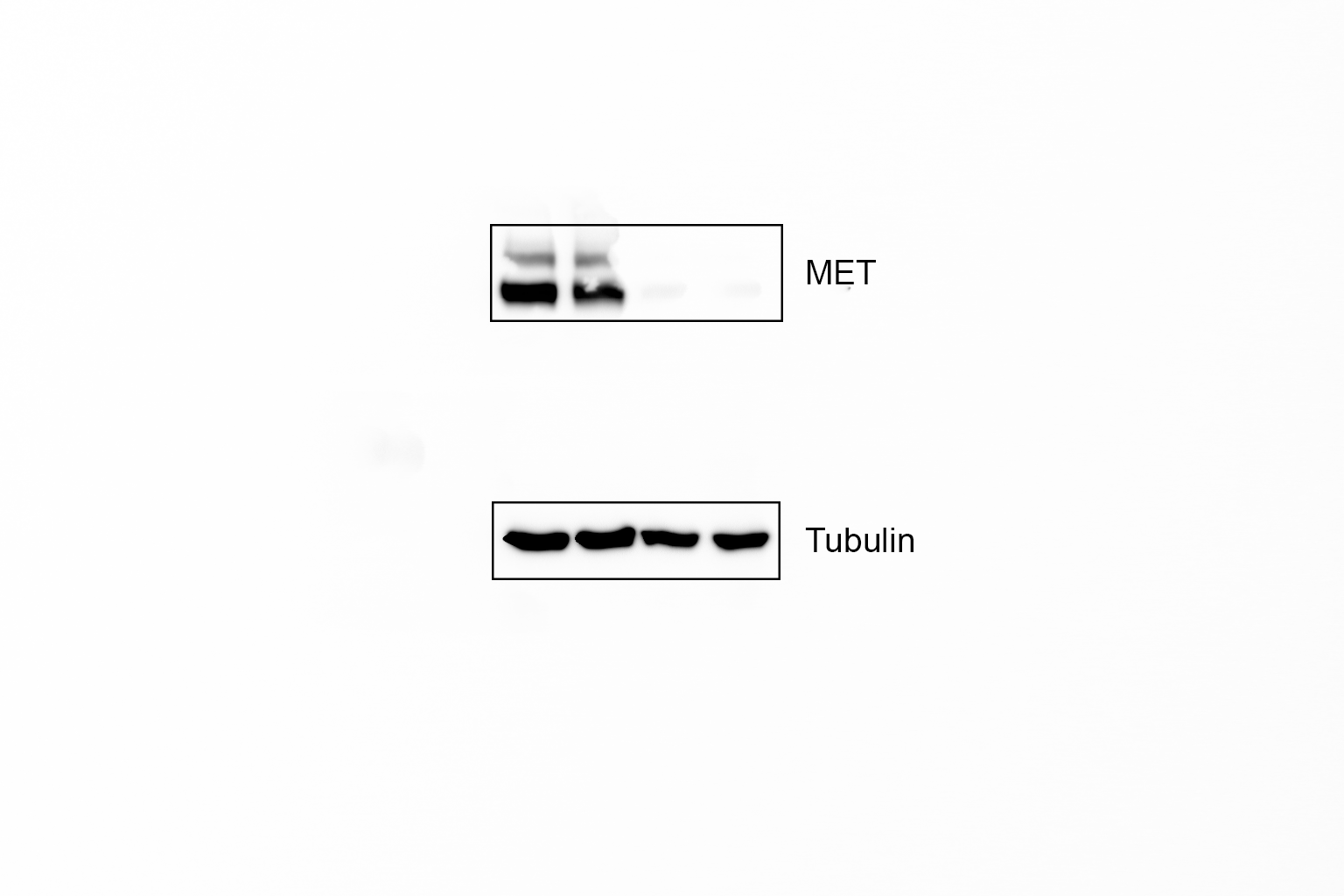

Supplement: Figure 3—source data 1. [file elife-70885-fig3-data1.zip › Figure 3-source data 1/Figure 3B/Figure 3B Input(Tub_MET)tif.tif]

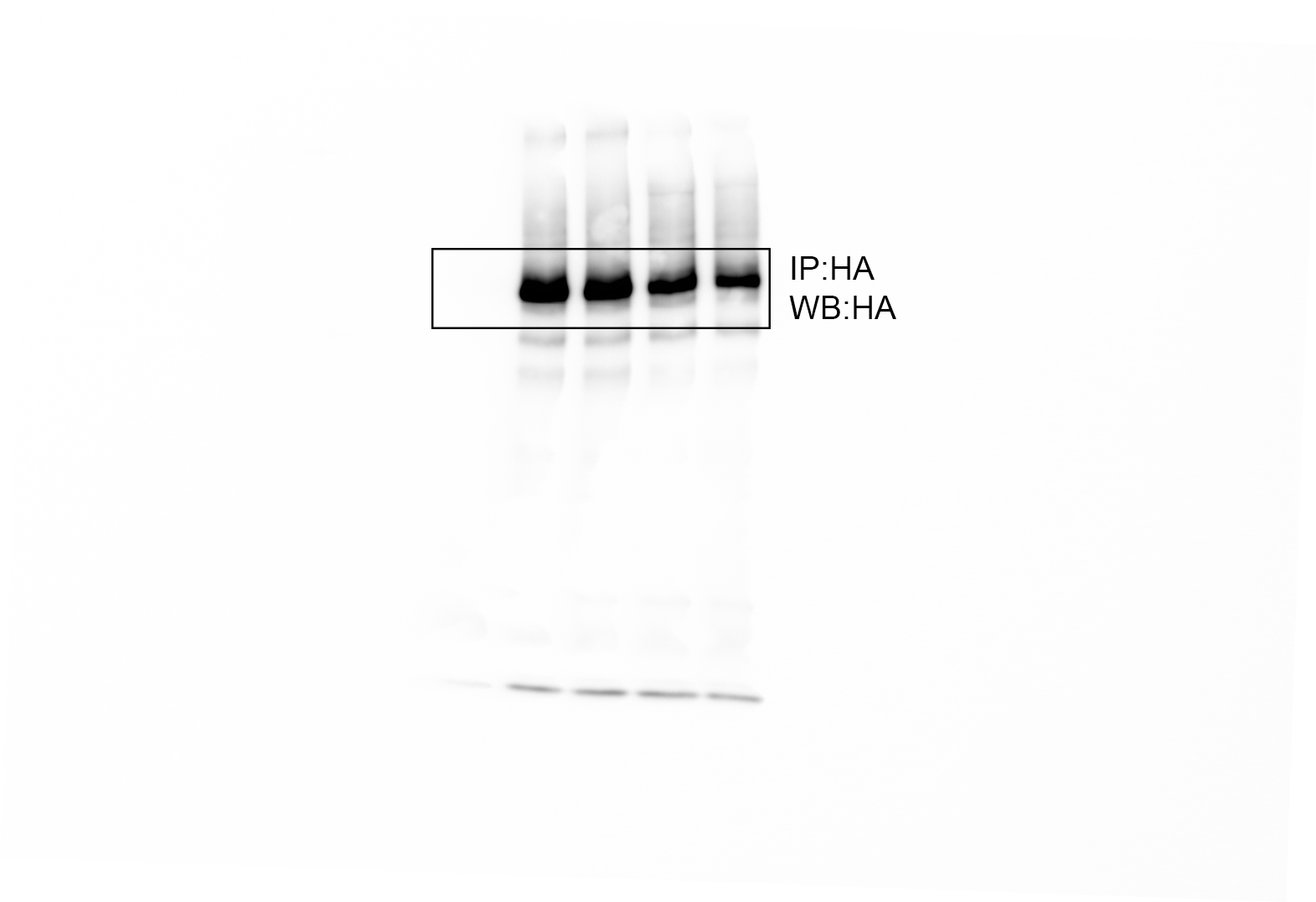

Supplement: Figure 3—source data 1. [file elife-70885-fig3-data1.zip › Figure 3-source data 1/Figure 3B/Figure 3B IP(HA)tif.tif]

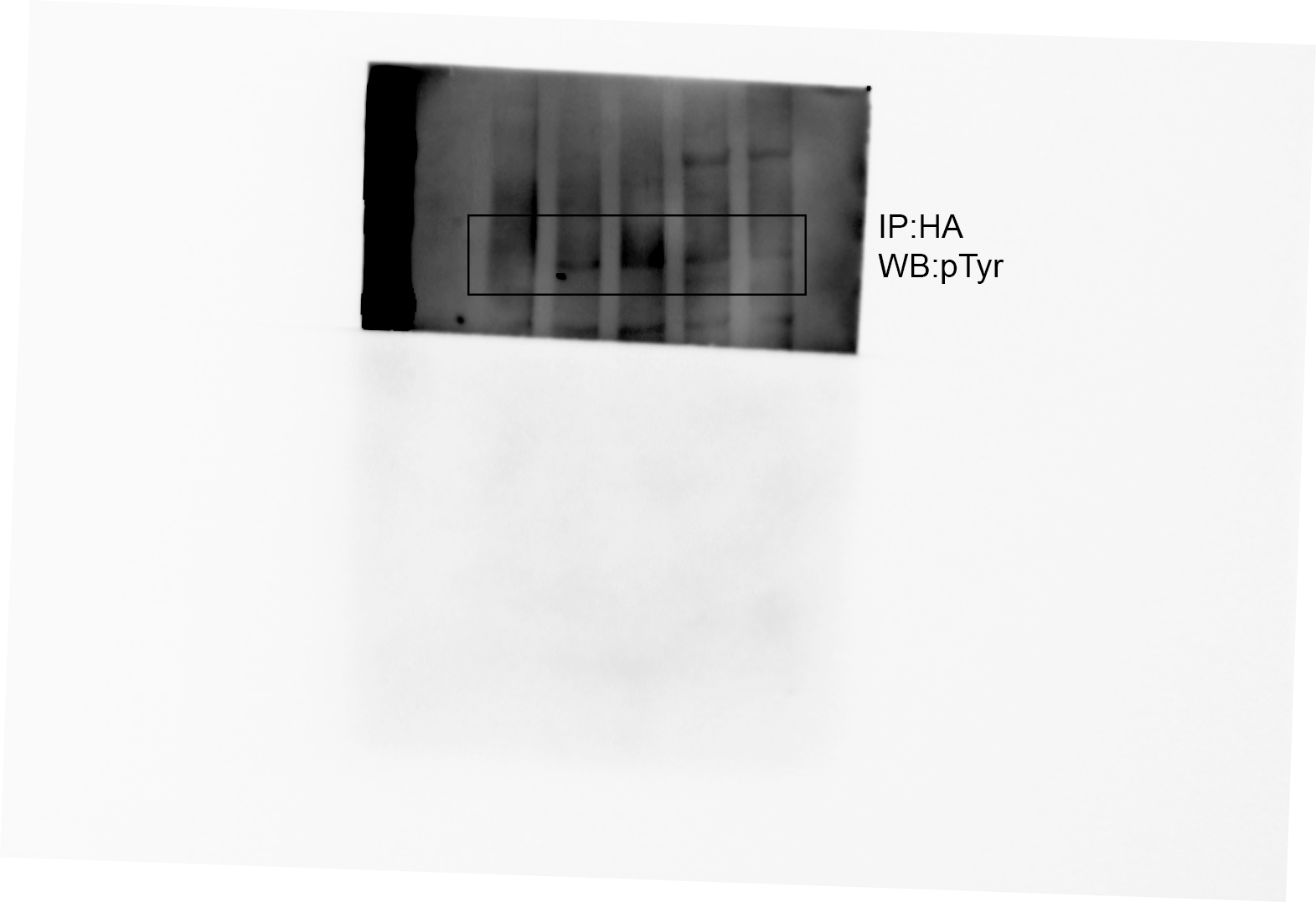

Supplement: Figure 3—source data 1. [file elife-70885-fig3-data1.zip › Figure 3-source data 1/Figure 3B/Figure 3B IP(pTyr)tif.tif]

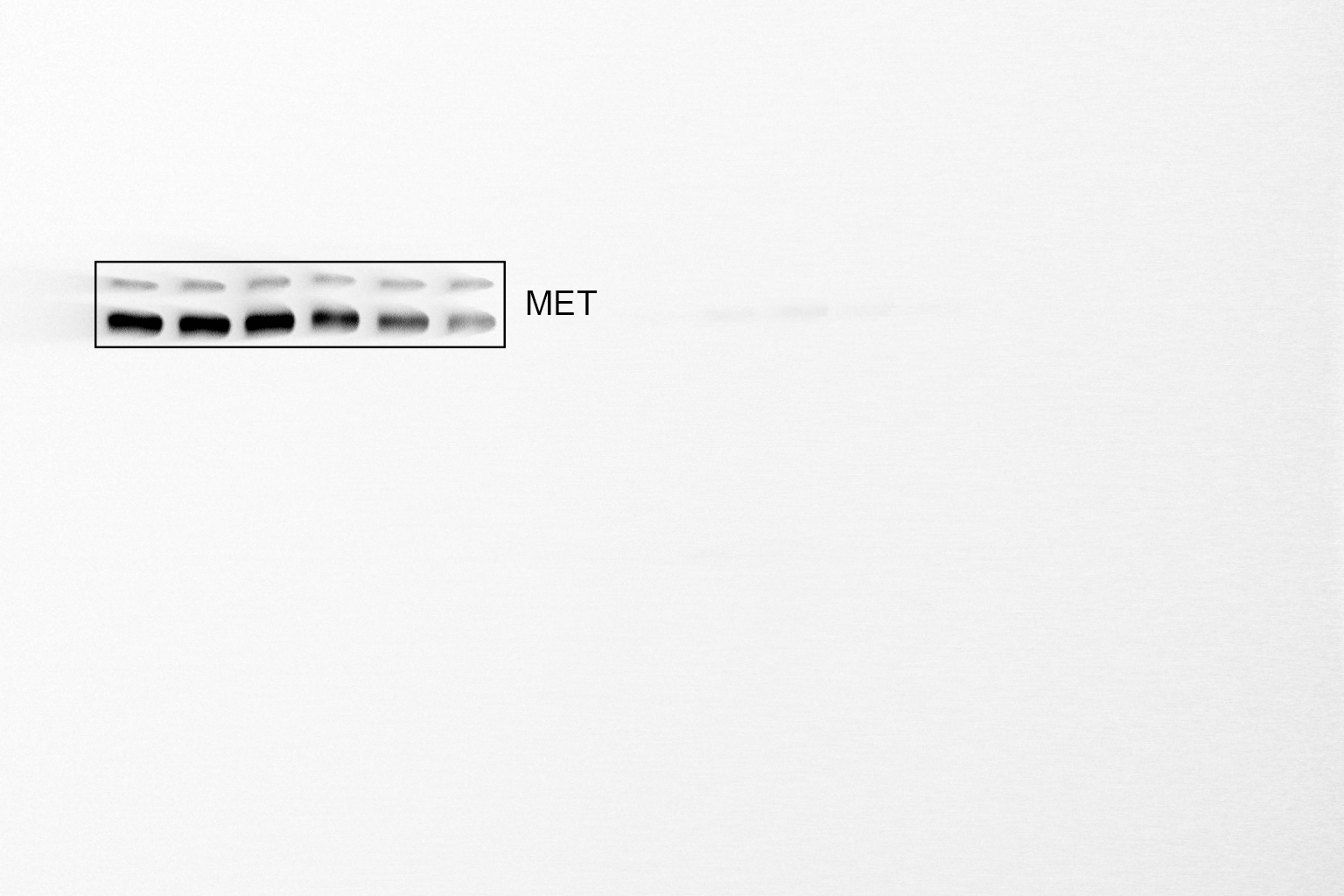

Supplement: Figure 3—source data 1. [file elife-70885-fig3-data1.zip › Figure 3-source data 1/Figure 3C/Figure 3C Input(MET).tif]

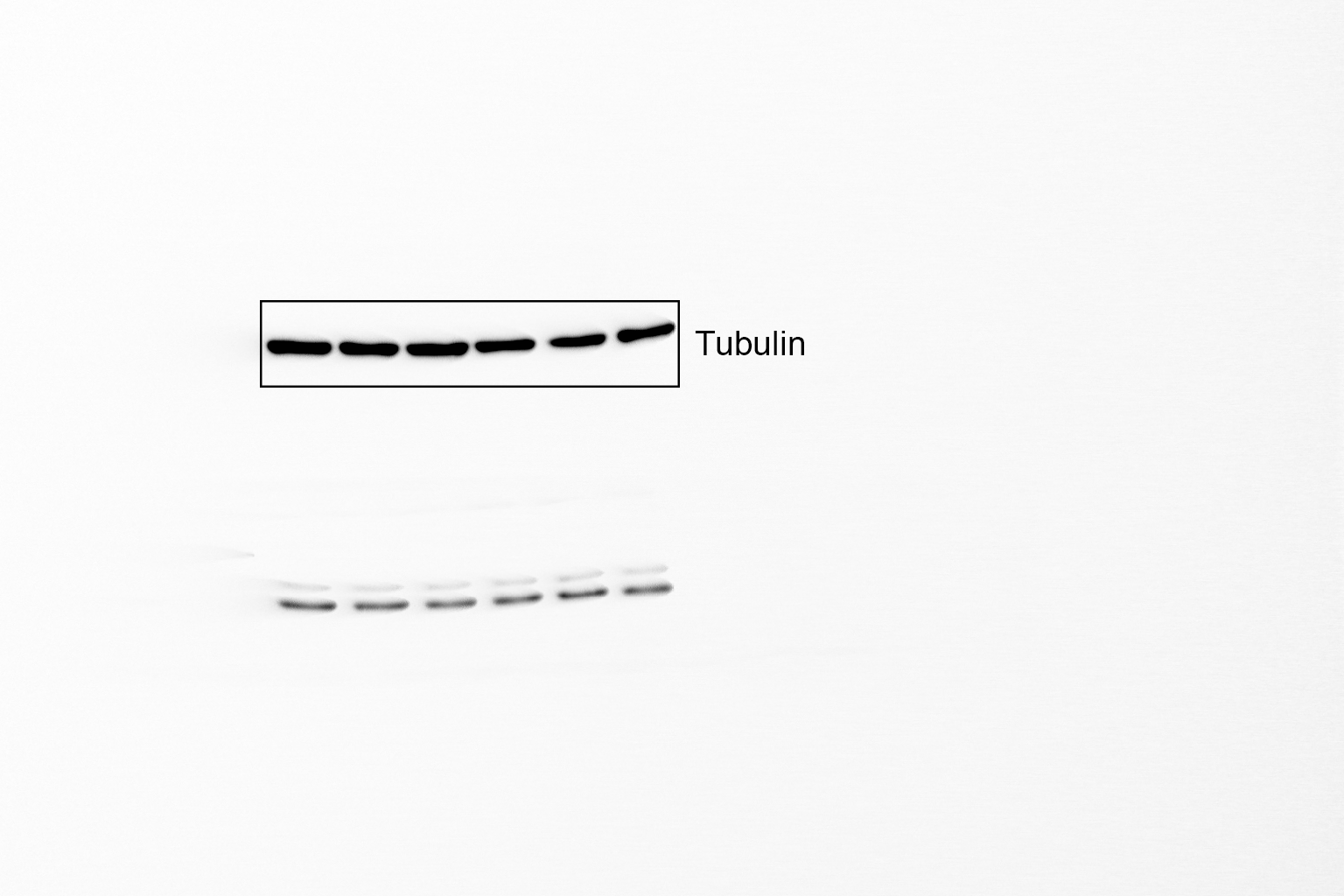

Supplement: Figure 3—source data 1. [file elife-70885-fig3-data1.zip › Figure 3-source data 1/Figure 3C/Figure 3C Input(Tub).tif]

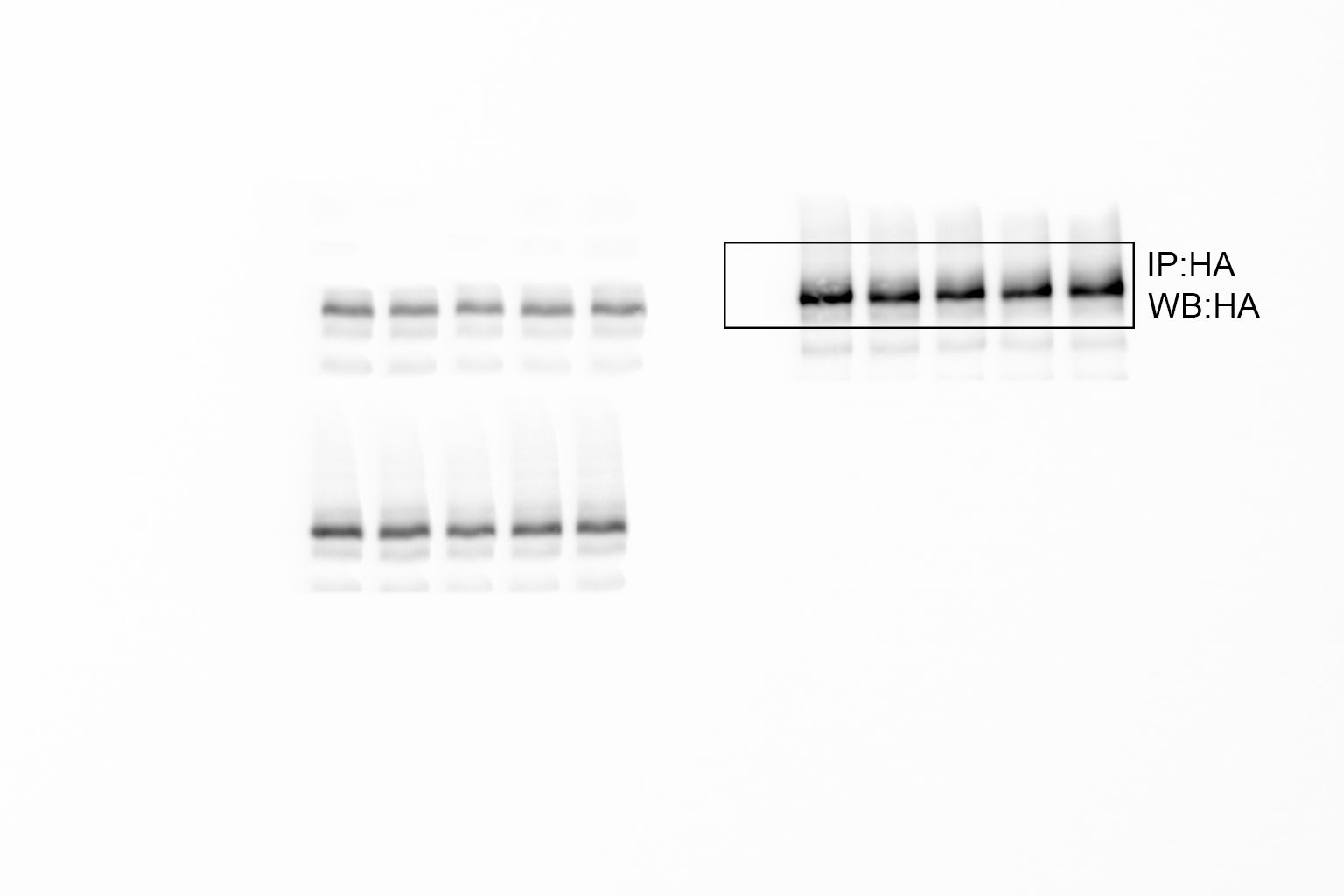

Supplement: Figure 3—source data 1. [file elife-70885-fig3-data1.zip › Figure 3-source data 1/Figure 3C/Figure 3C IP(HA).tif]

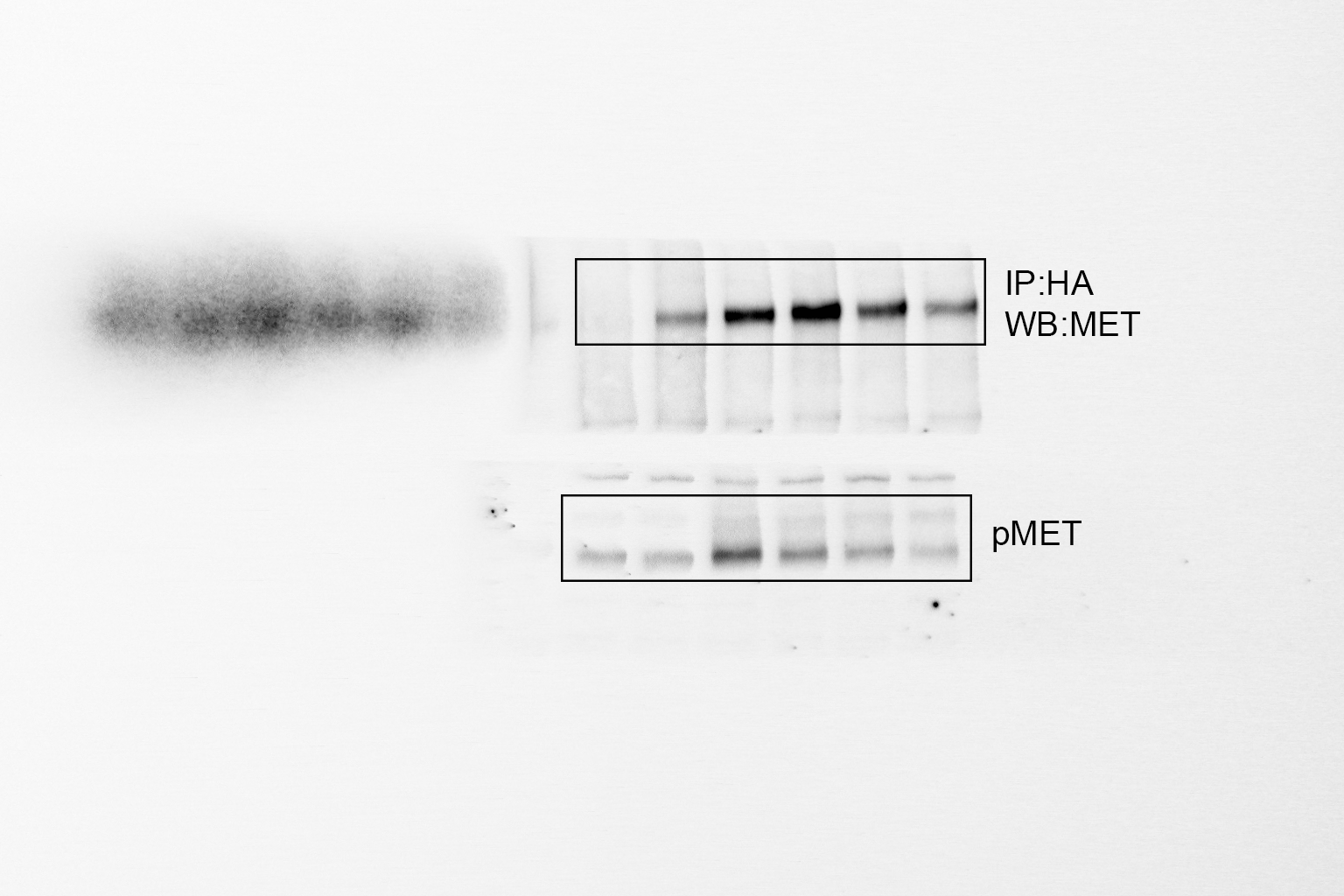

Supplement: Figure 3—source data 1. [file elife-70885-fig3-data1.zip › Figure 3-source data 1/Figure 3C/Figure 3C IP(MET)_Input(pMET).tif]

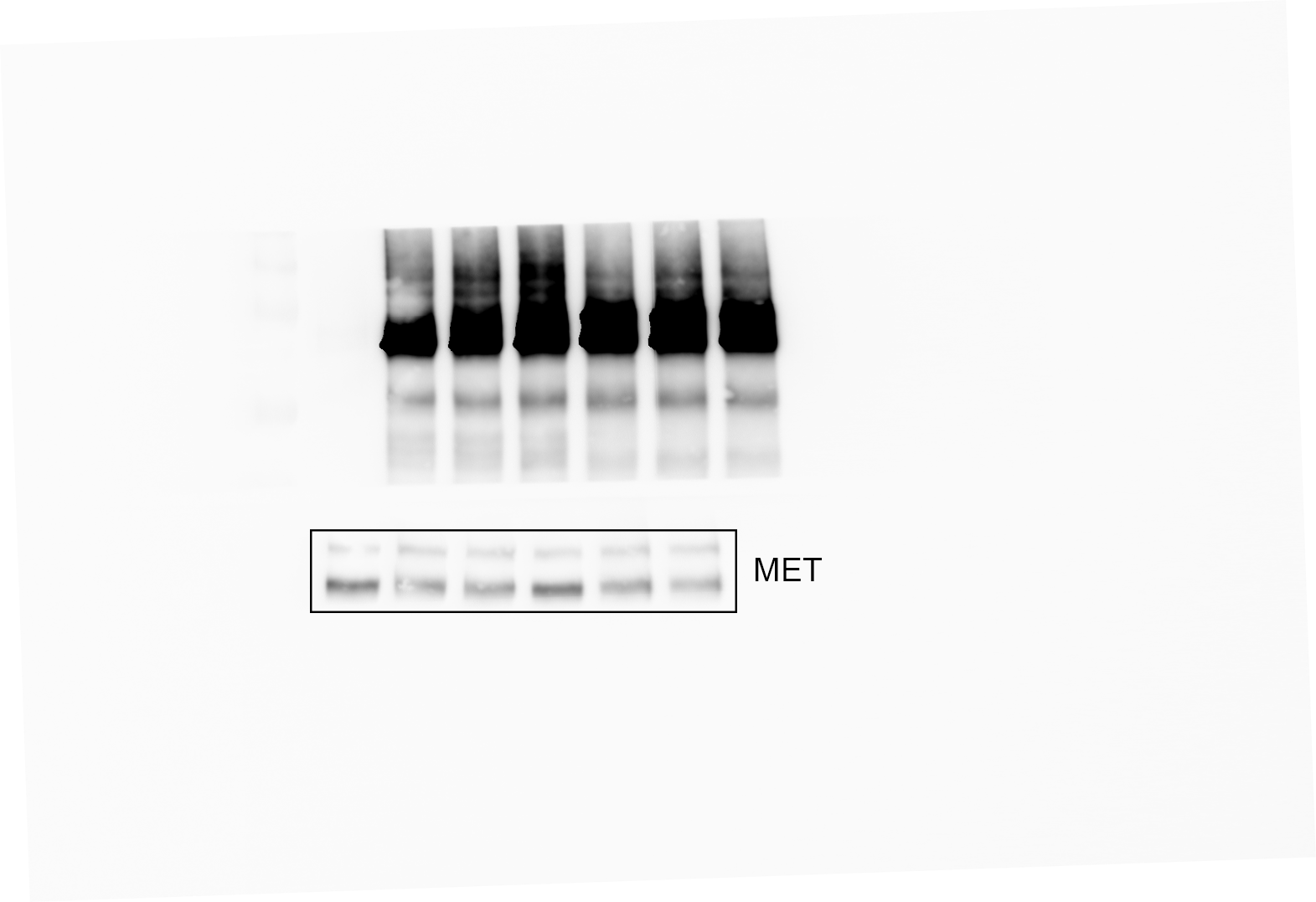

Supplement: Figure 3—source data 1. [file elife-70885-fig3-data1.zip › Figure 3-source data 1/Figure 3D/Figure 3D Input(MET).tif]

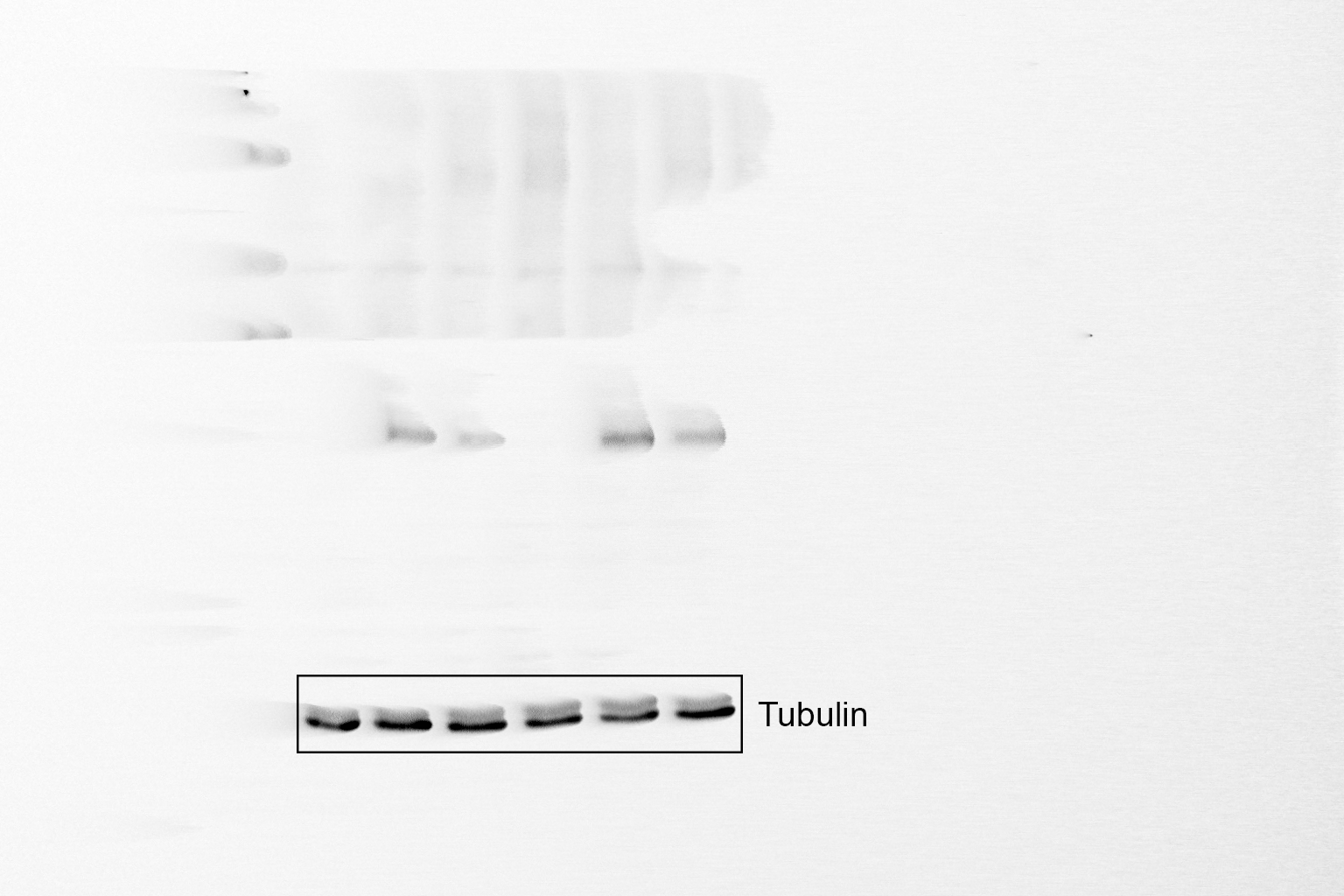

Supplement: Figure 3—source data 1. [file elife-70885-fig3-data1.zip › Figure 3-source data 1/Figure 3D/Figure 3D Input(Tub).tif]

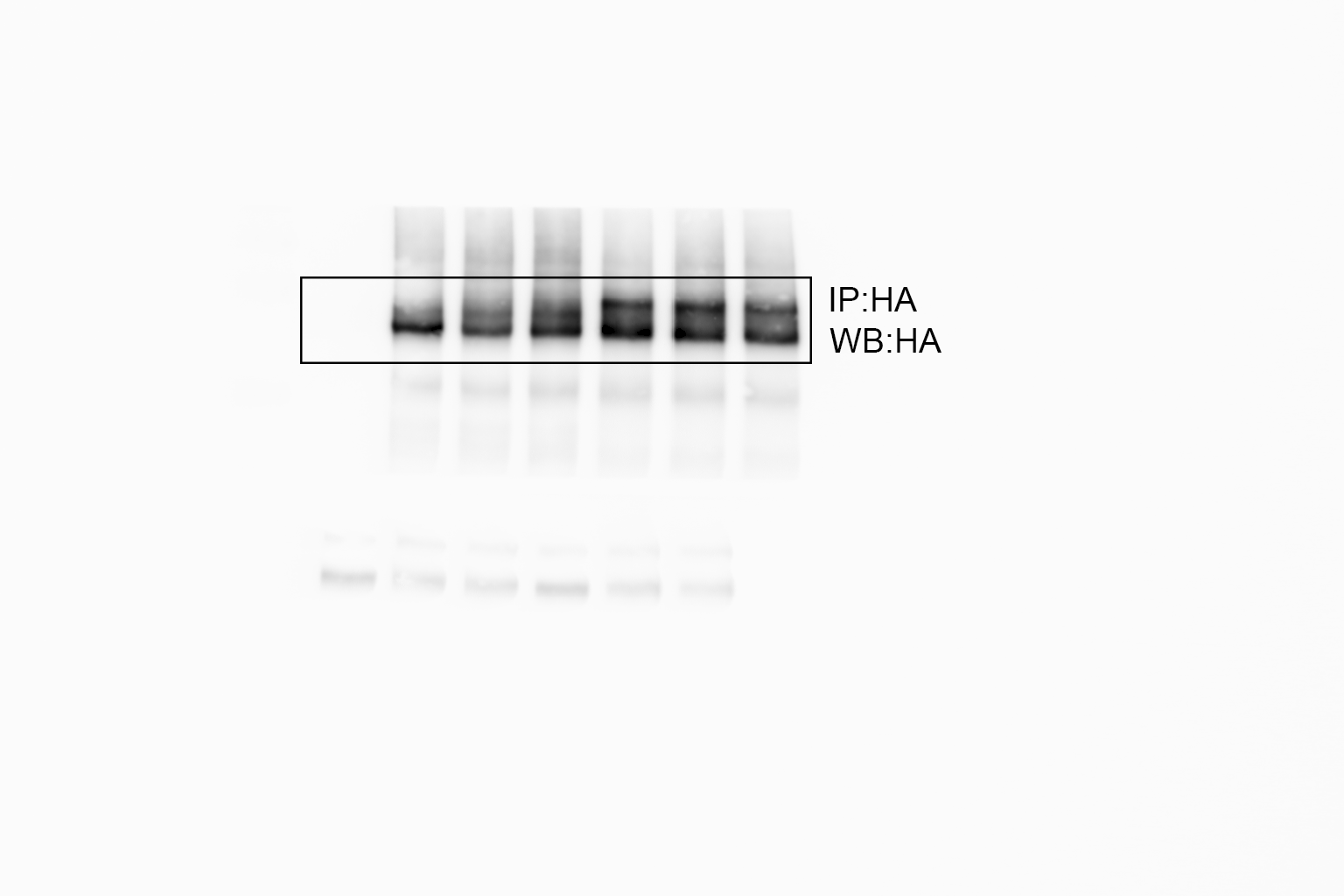

Supplement: Figure 3—source data 1. [file elife-70885-fig3-data1.zip › Figure 3-source data 1/Figure 3D/Figure 3D IP(HA).tif]

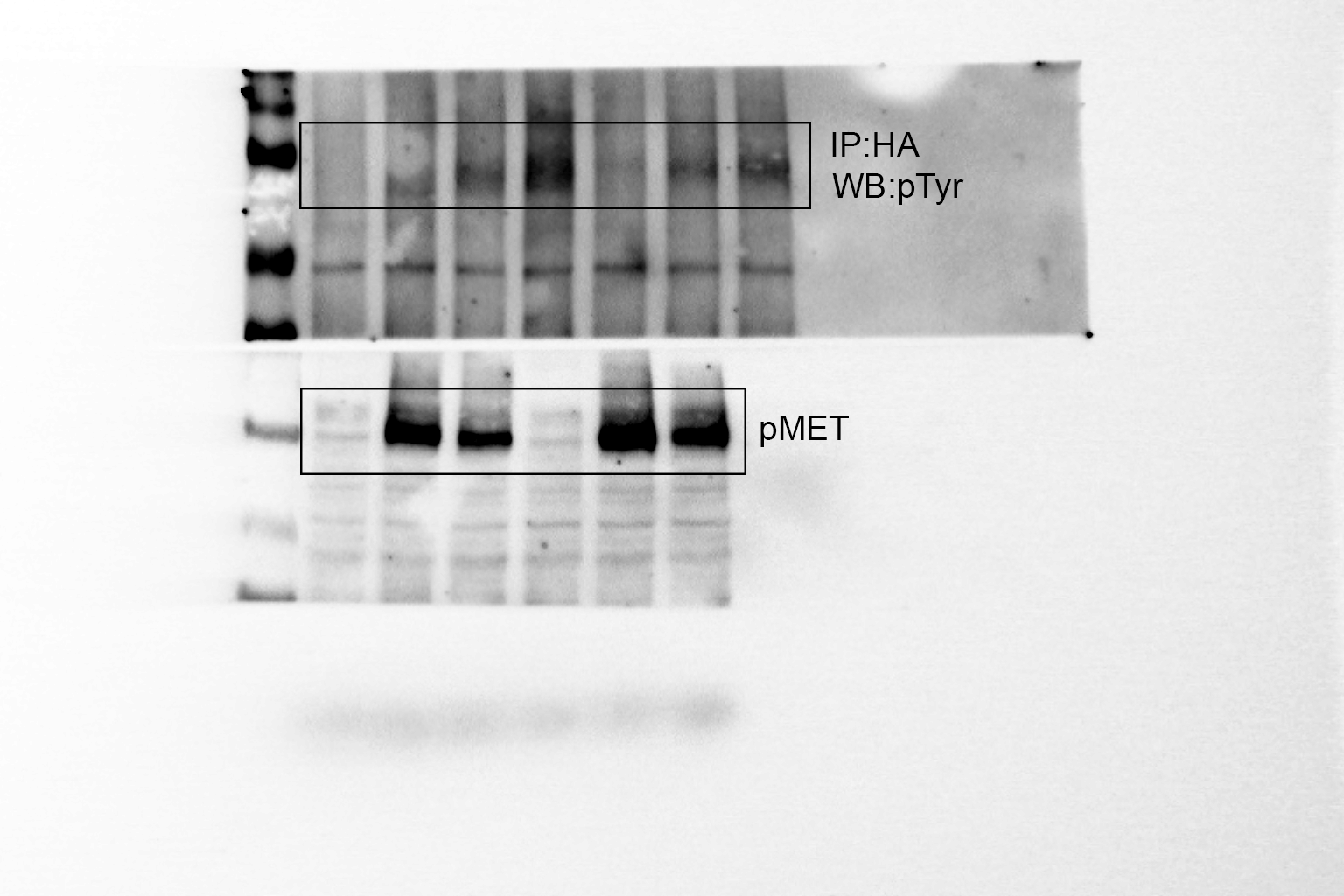

Supplement: Figure 3—source data 1. [file elife-70885-fig3-data1.zip › Figure 3-source data 1/Figure 3D/Figure 3D IP(pTyr)_Input(pMET).tif]

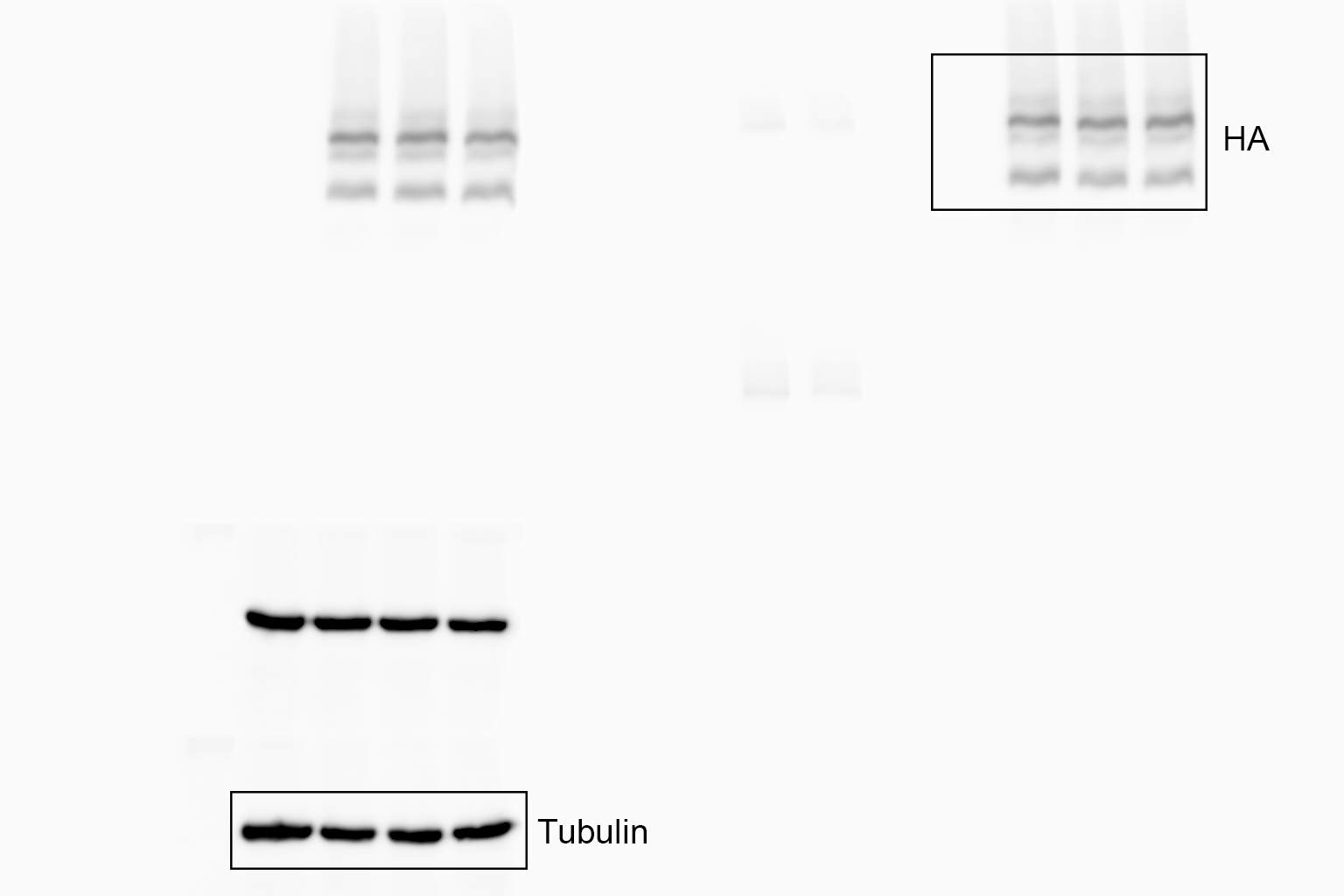

Supplement: Figure 4—source data 1. [file elife-70885-fig4-data1.zip › Figure 4-source data 1/Figure 4A/Figure 4A Input (Tub_HA).tif]

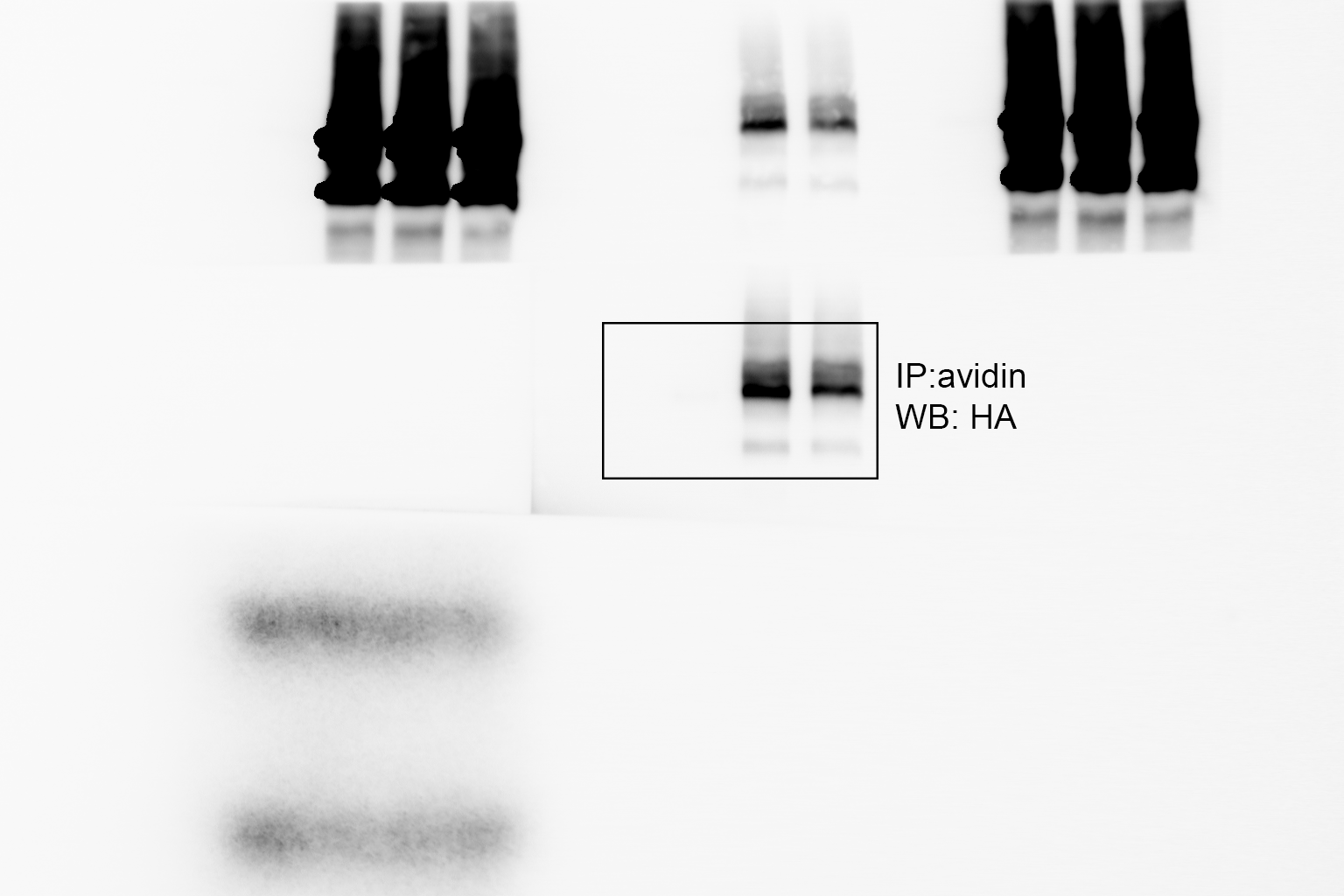

Supplement: Figure 4—source data 1. [file elife-70885-fig4-data1.zip › Figure 4-source data 1/Figure 4A/Figure 4A IP (HA).tif]

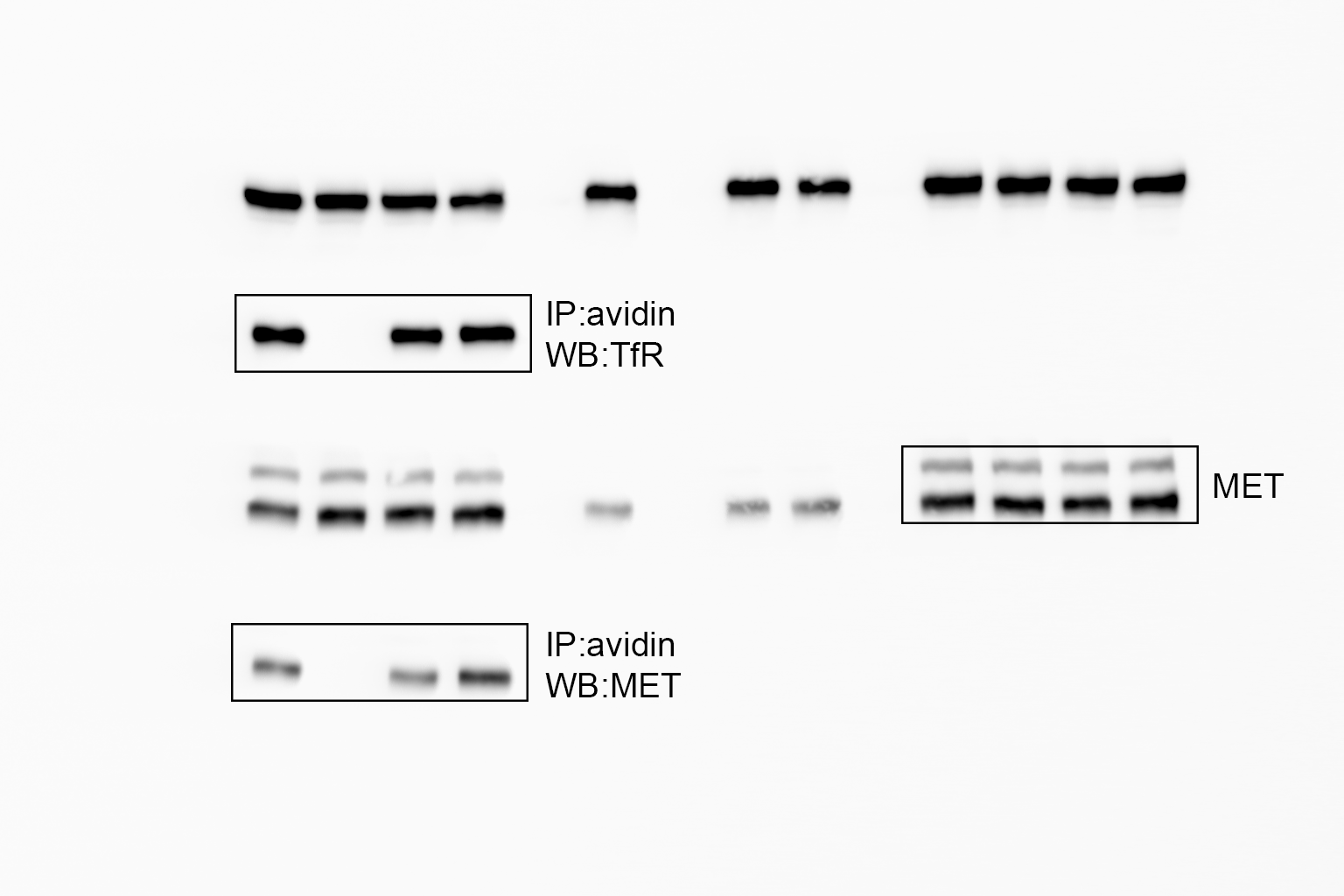

Supplement: Figure 4—source data 1. [file elife-70885-fig4-data1.zip › Figure 4-source data 1/Figure 4A/Figure 4A IP(TfR_MET)_ Input(MET).tif]

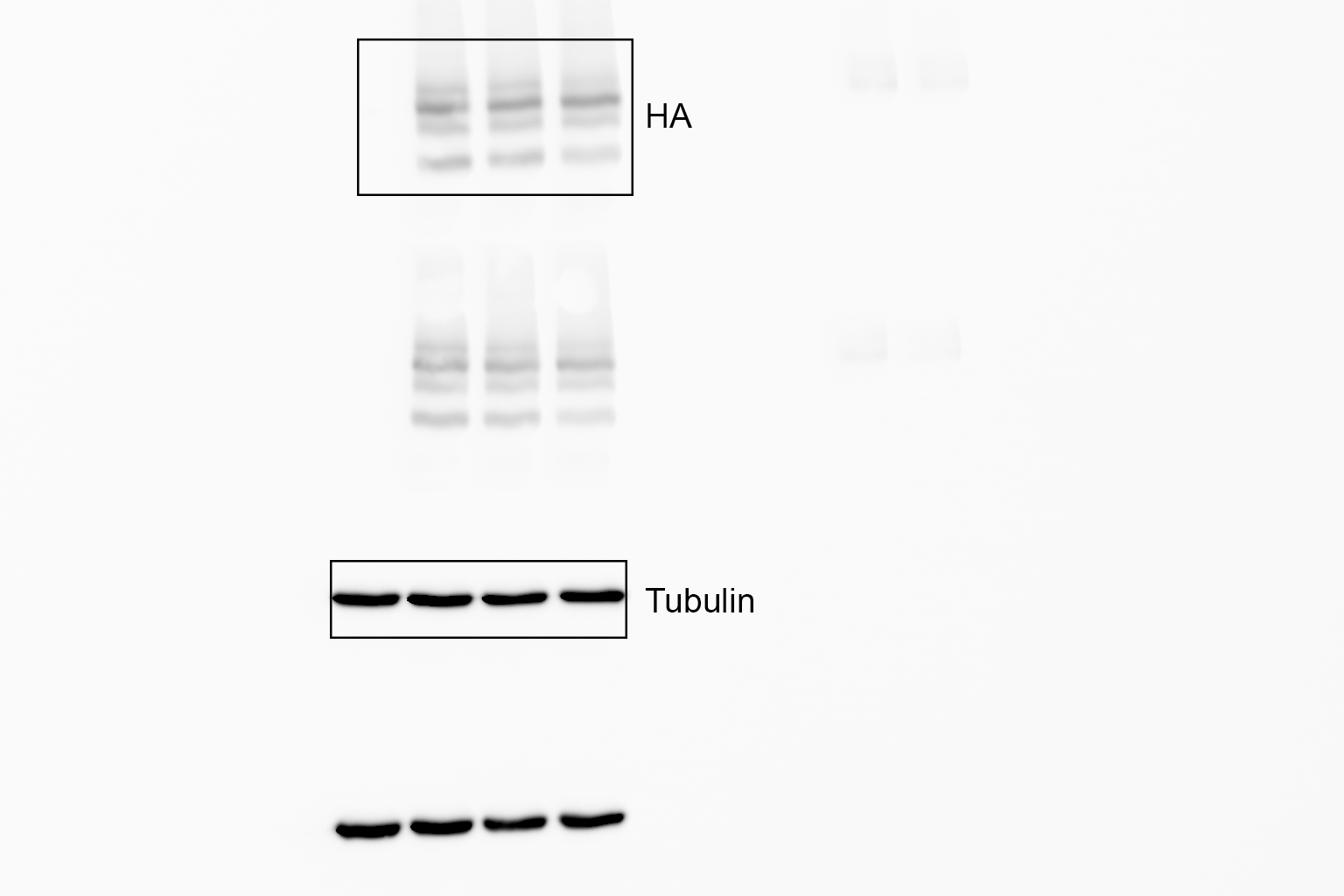

Supplement: Figure 4—source data 1. [file elife-70885-fig4-data1.zip › Figure 4-source data 1/Figure 4B/Figure 4B Input(HA_Tub).tif]

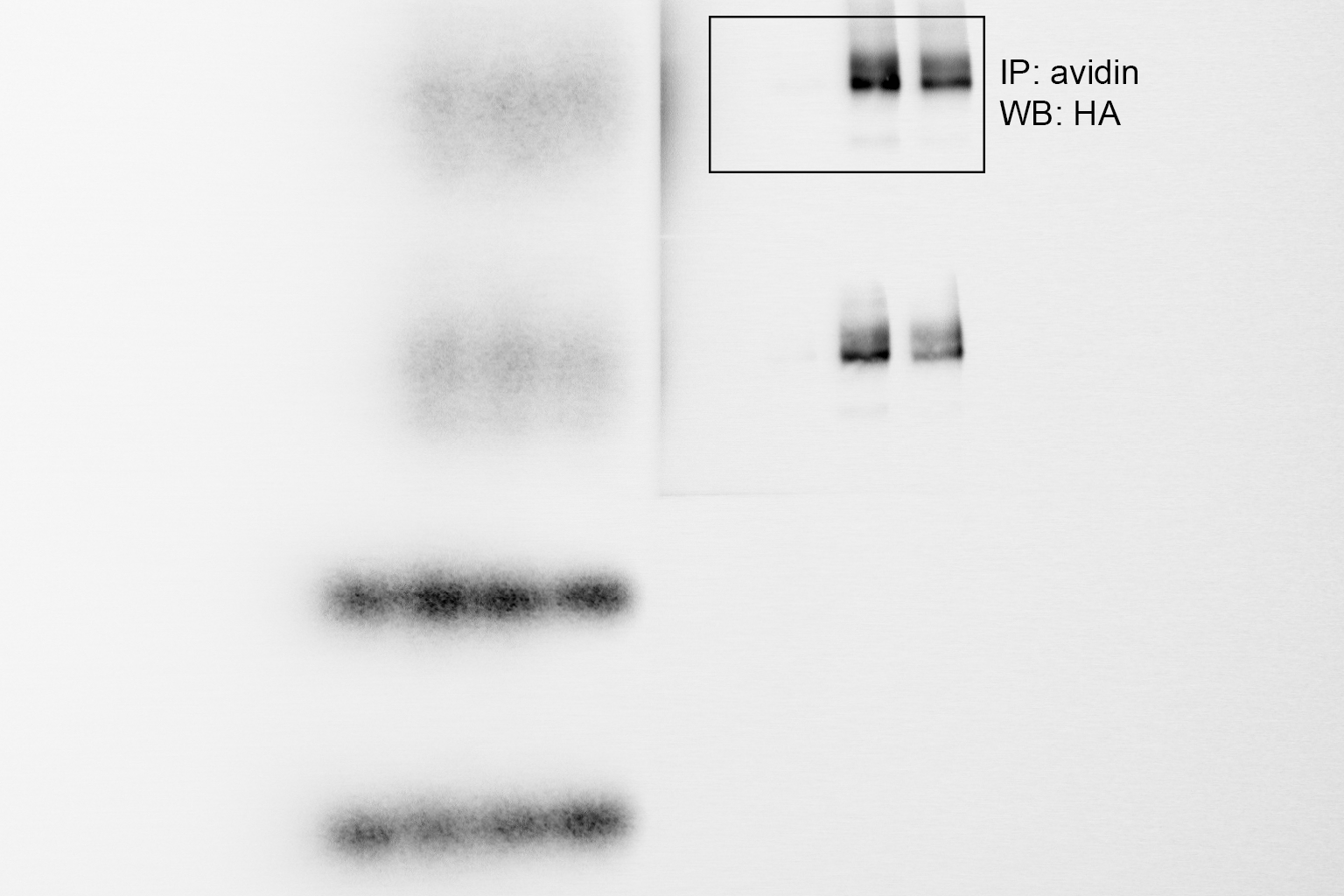

Supplement: Figure 4—source data 1. [file elife-70885-fig4-data1.zip › Figure 4-source data 1/Figure 4B/Figure 4B IP(HA).tif]

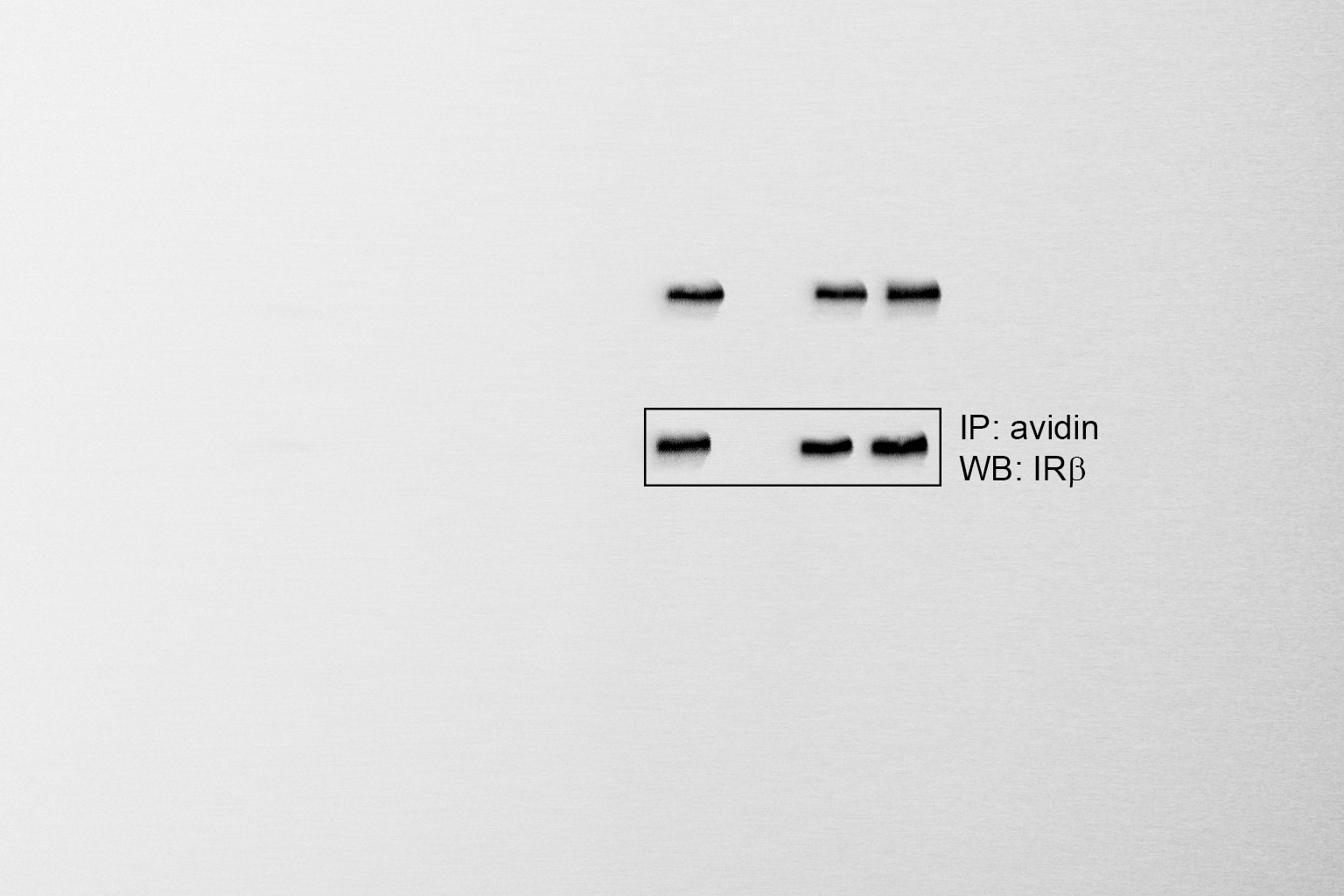

Supplement: Figure 4—source data 1. [file elife-70885-fig4-data1.zip › Figure 4-source data 1/Figure 4B/Figure 4B IP(IRb).tif]

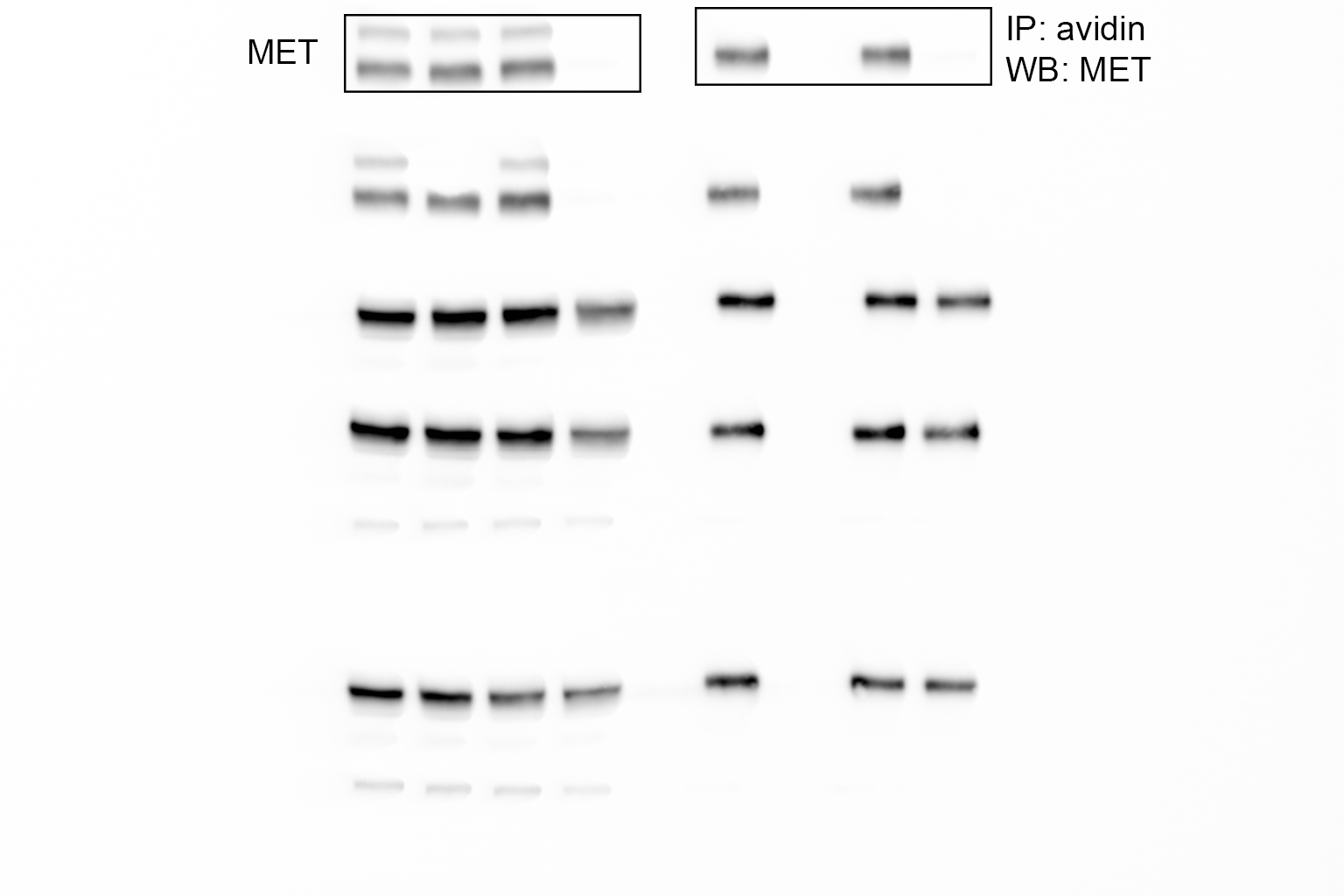

Supplement: Figure 4—source data 1. [file elife-70885-fig4-data1.zip › Figure 4-source data 1/Figure 4B/Figure 4B IP(MET)_ Input (MET).tif]

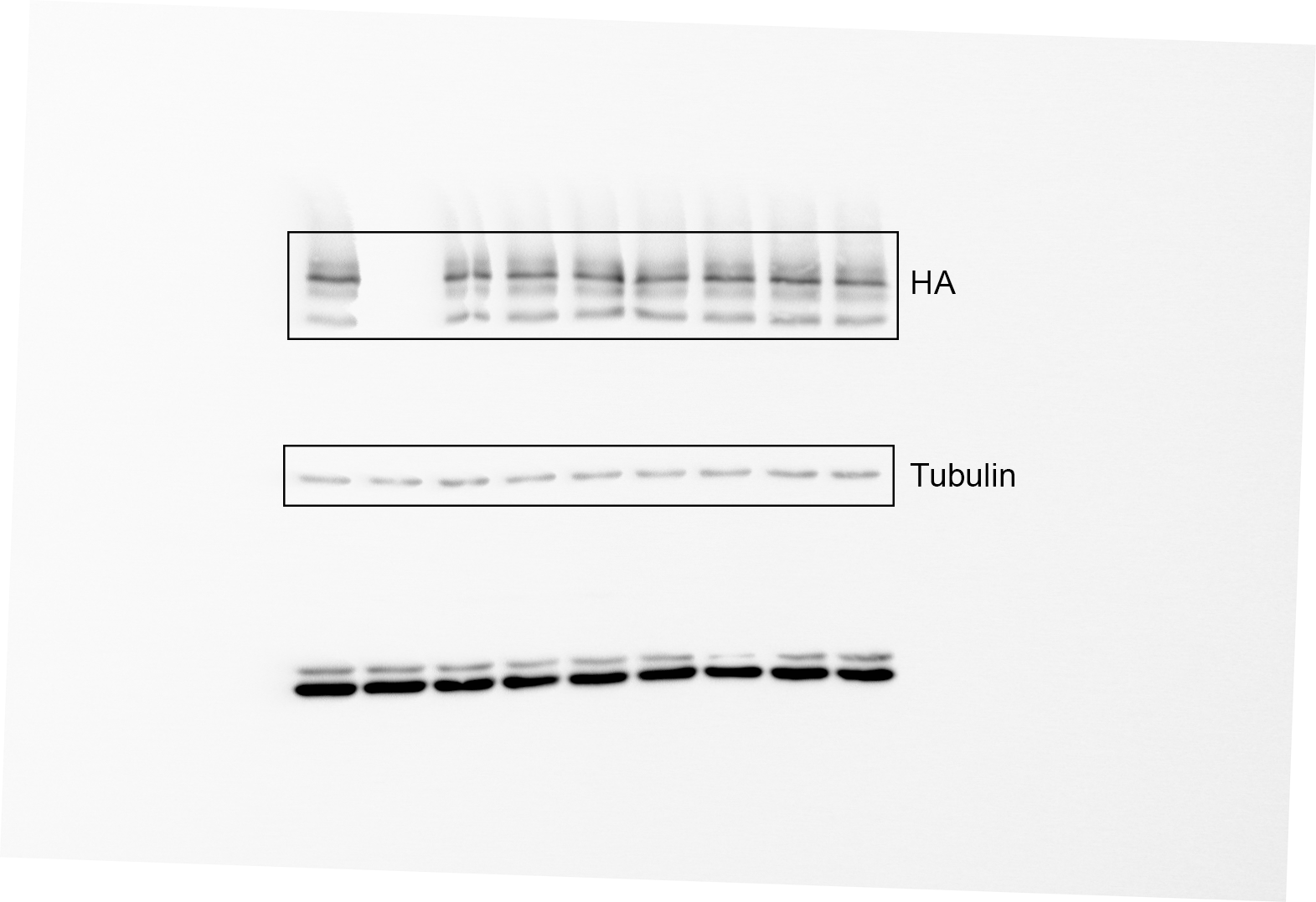

Supplement: Figure 4—source data 1. [file elife-70885-fig4-data1.zip › Figure 4-source data 1/Figure 4C/Figure 4C Input (HA_Tub).tif]

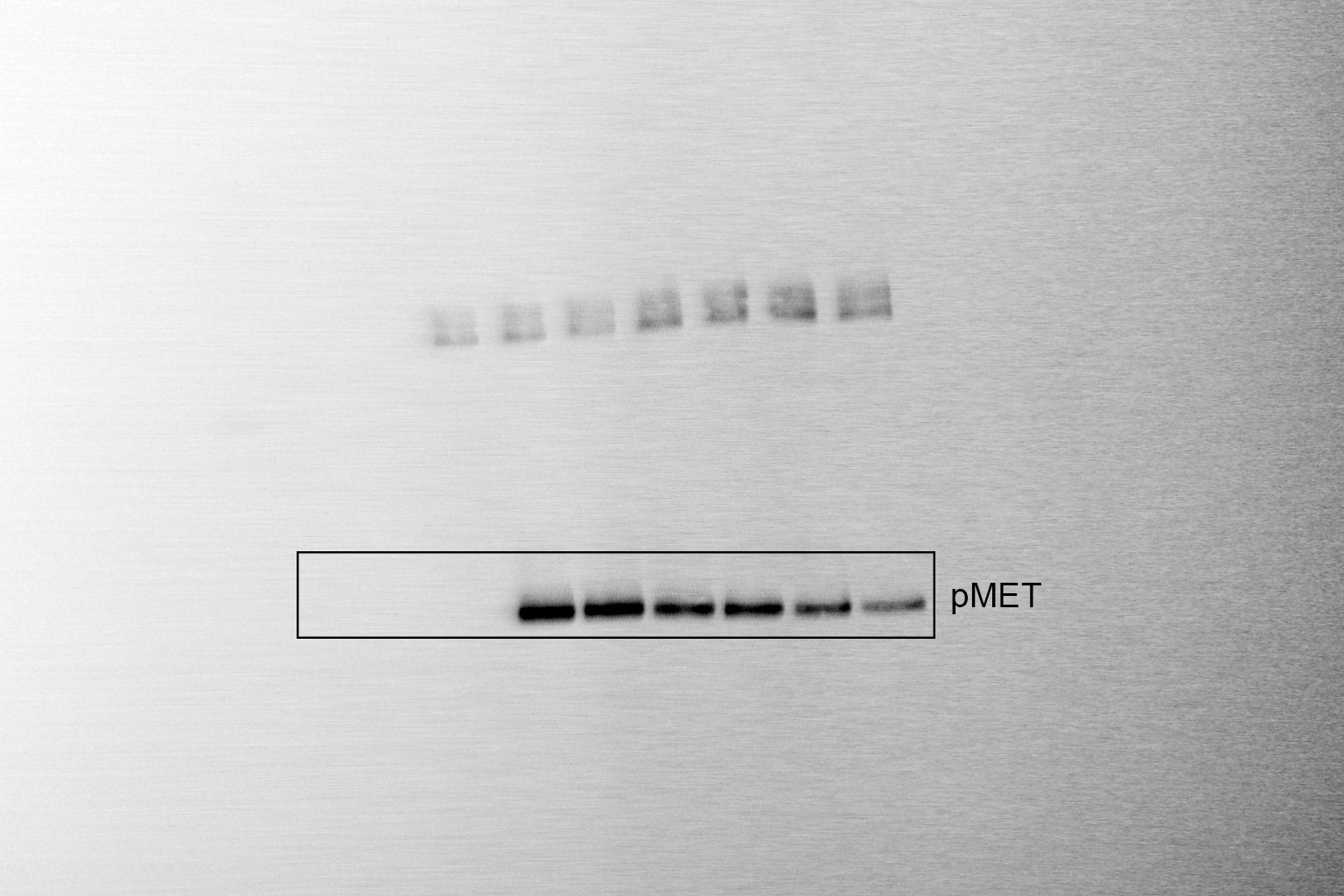

Supplement: Figure 4—source data 1. [file elife-70885-fig4-data1.zip › Figure 4-source data 1/Figure 4C/Figure 4C Input (pMET).tif]

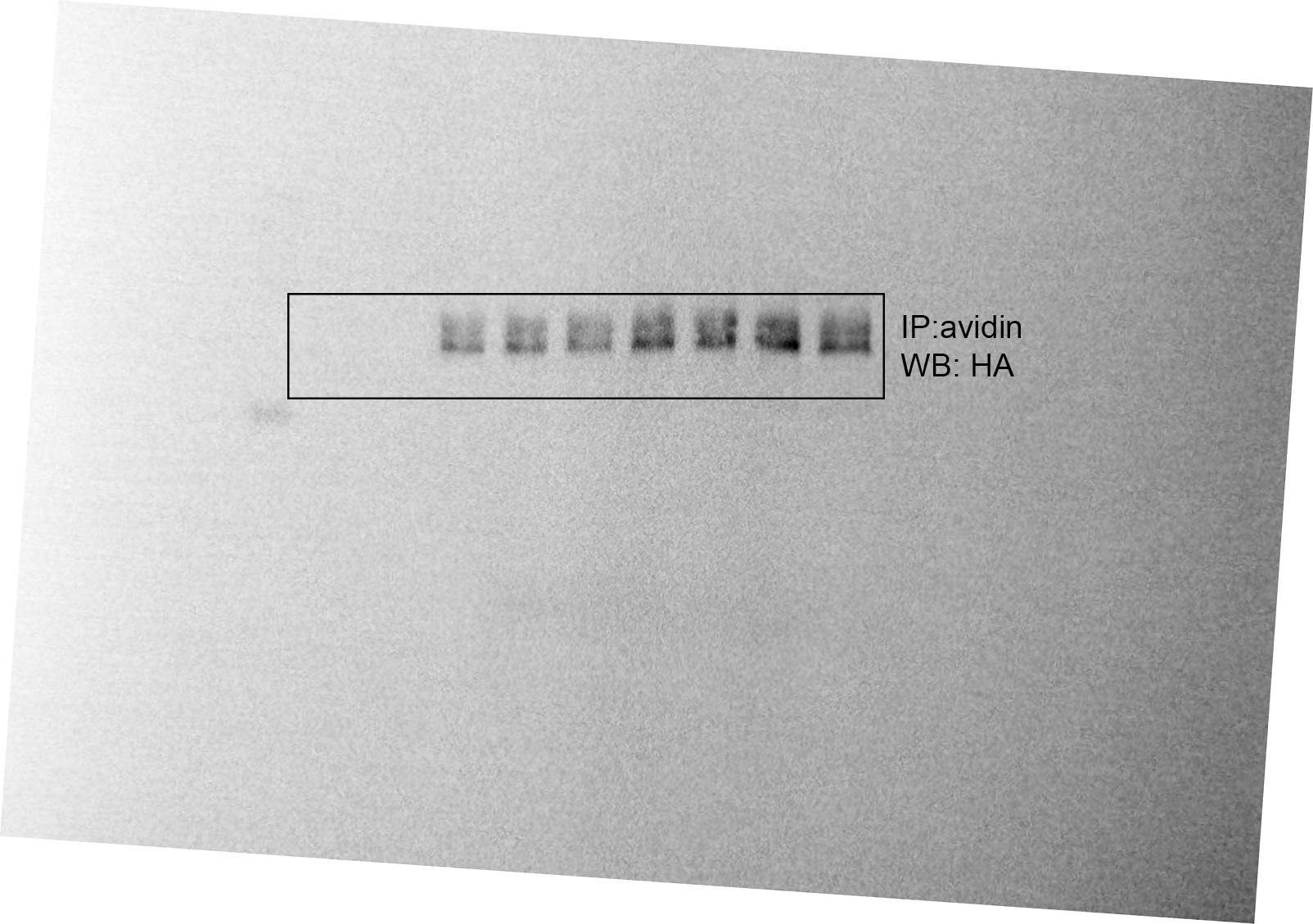

Supplement: Figure 4—source data 1. [file elife-70885-fig4-data1.zip › Figure 4-source data 1/Figure 4C/Figure 4C IP (HA).tif]

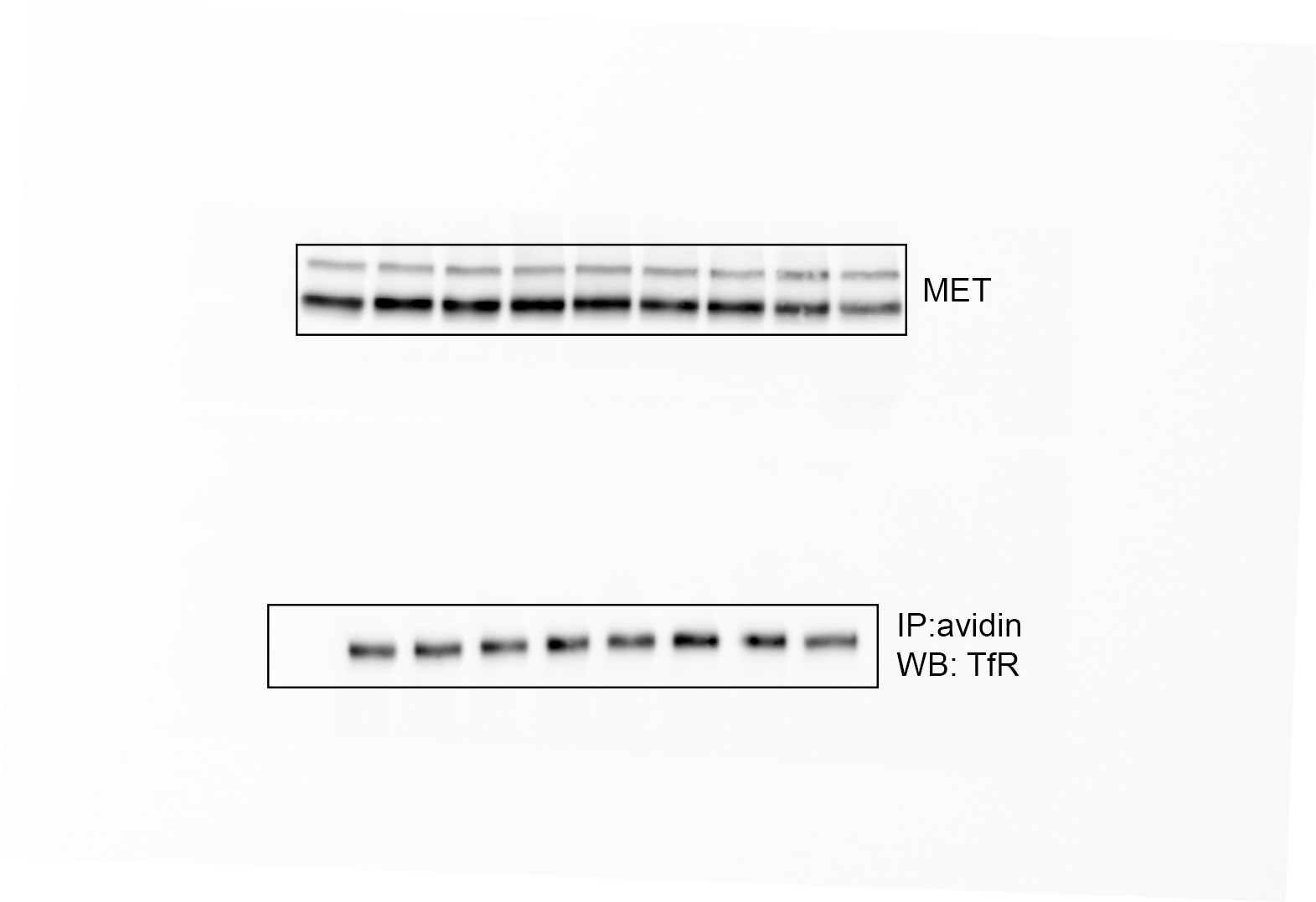

Supplement: Figure 4—source data 1. [file elife-70885-fig4-data1.zip › Figure 4-source data 1/Figure 4C/Figure 4C IP (TfR)_Input (MET).tif]

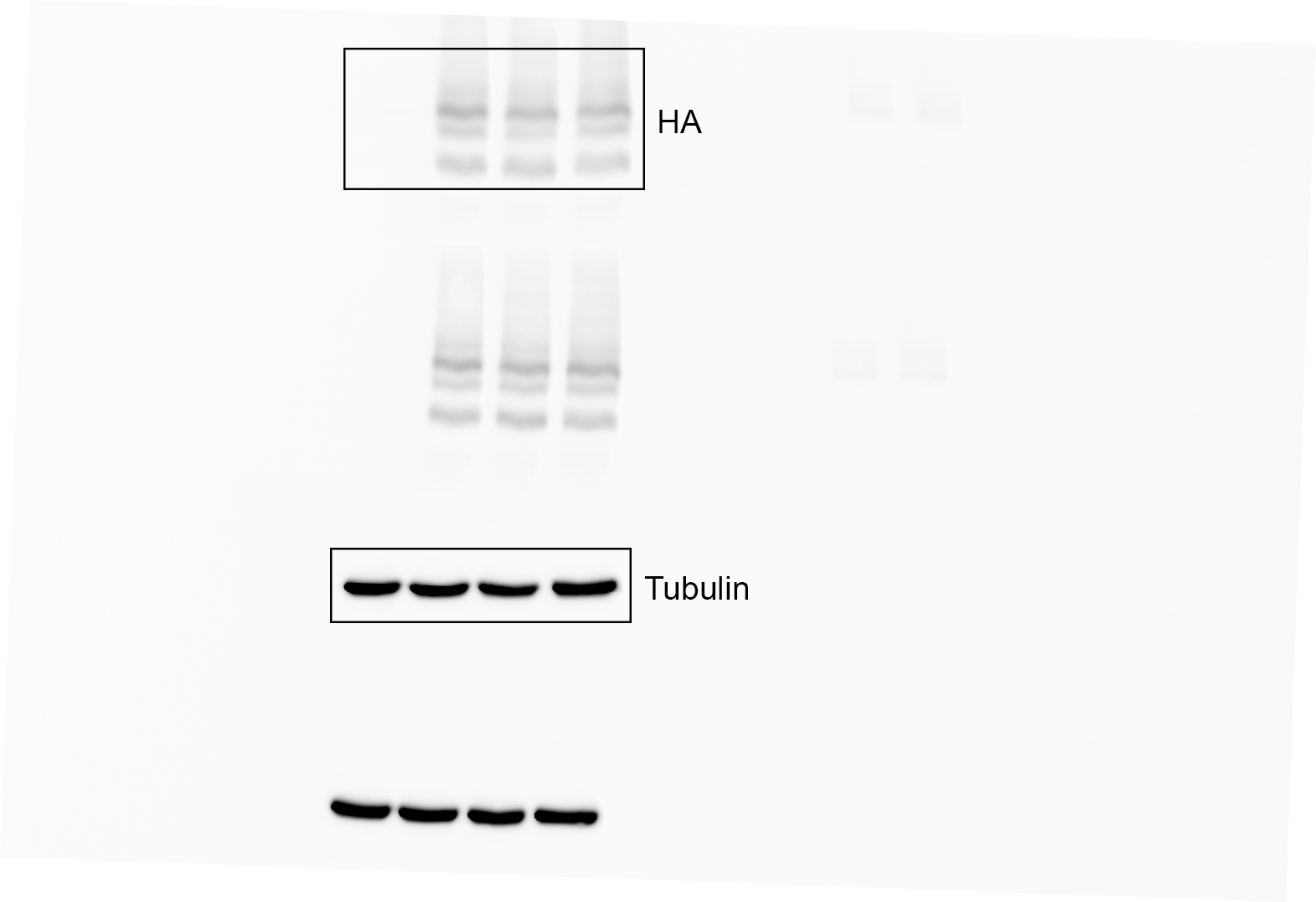

Supplement: Figure 4—figure supplement 1—source data 1. [file elife-70885-fig4-figsupp1-data1.zip › Figure 4-Figure supplement 1- source data 1/Figure 4-Figure supplement 1A/Figure4-Figure supplement 1_ Input (HA_Tub).tif]

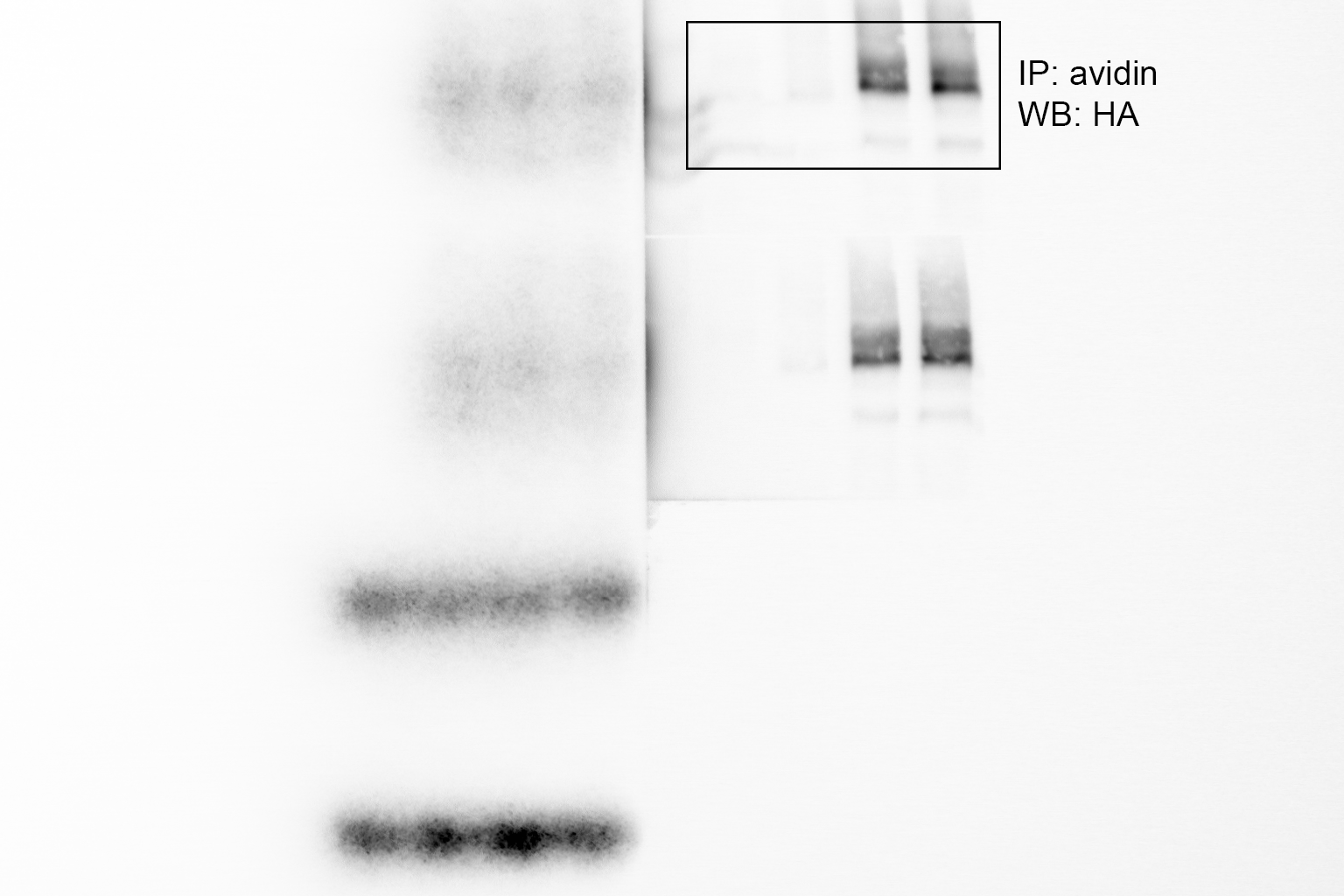

Supplement: Figure 4—figure supplement 1—source data 1. [file elife-70885-fig4-figsupp1-data1.zip › Figure 4-Figure supplement 1- source data 1/Figure 4-Figure supplement 1A/Figure4-Figure supplement 1_ IP (HA).tif]

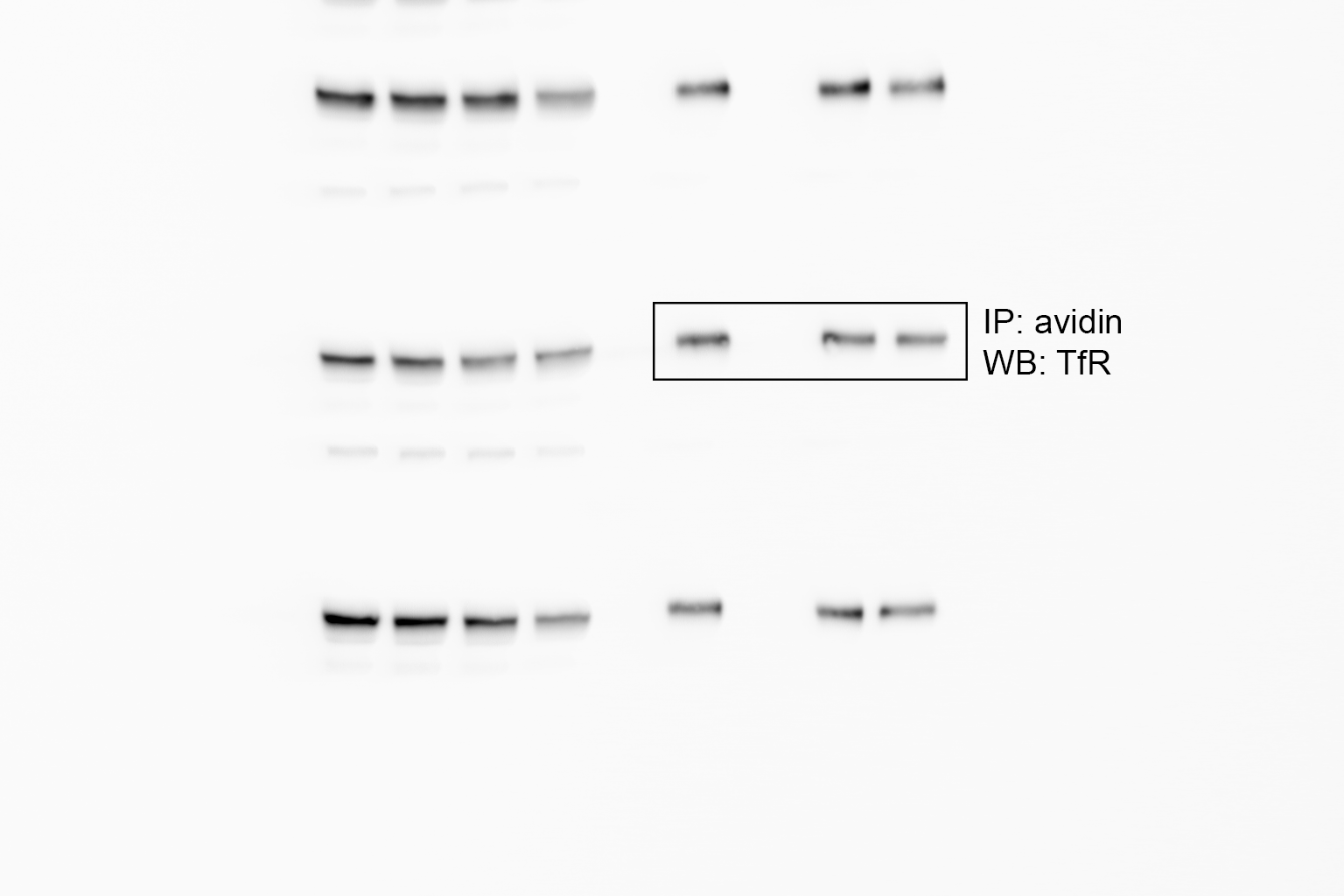

Supplement: Figure 4—figure supplement 1—source data 1. [file elife-70885-fig4-figsupp1-data1.zip › Figure 4-Figure supplement 1- source data 1/Figure 4-Figure supplement 1A/Figure4-Figure supplement 1_ IP (TfR).tif]

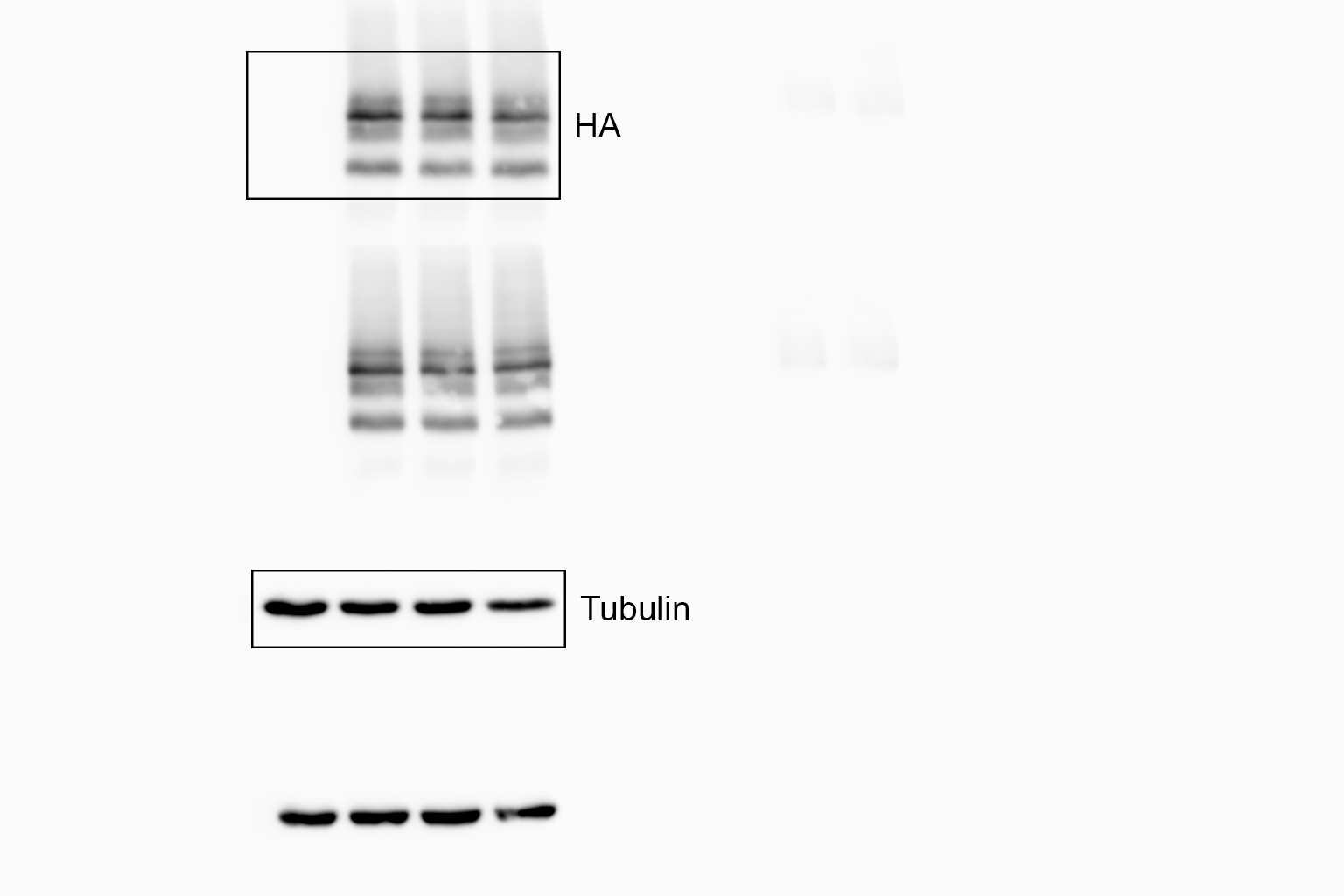

Supplement: Figure 4—figure supplement 1—source data 1. [file elife-70885-fig4-figsupp1-data1.zip › Figure 4-Figure supplement 1- source data 1/Figure 4-Figure supplement 1B/Figure 4-Figure supplement 1B_Input (HA_Tub).tif]

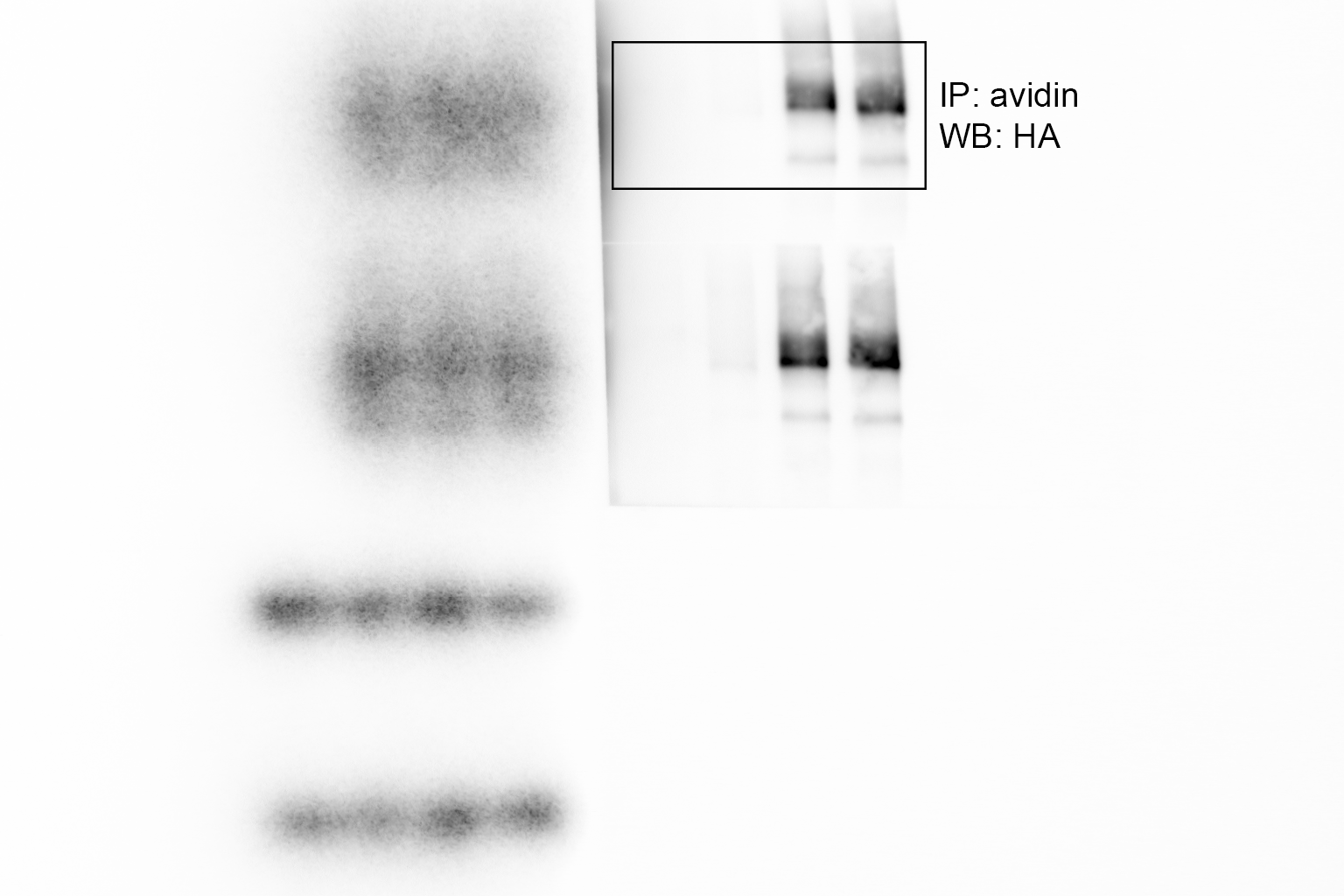

Supplement: Figure 4—figure supplement 1—source data 1. [file elife-70885-fig4-figsupp1-data1.zip › Figure 4-Figure supplement 1- source data 1/Figure 4-Figure supplement 1B/Figure 4-Figure supplement 1B_IP (HA).tif]

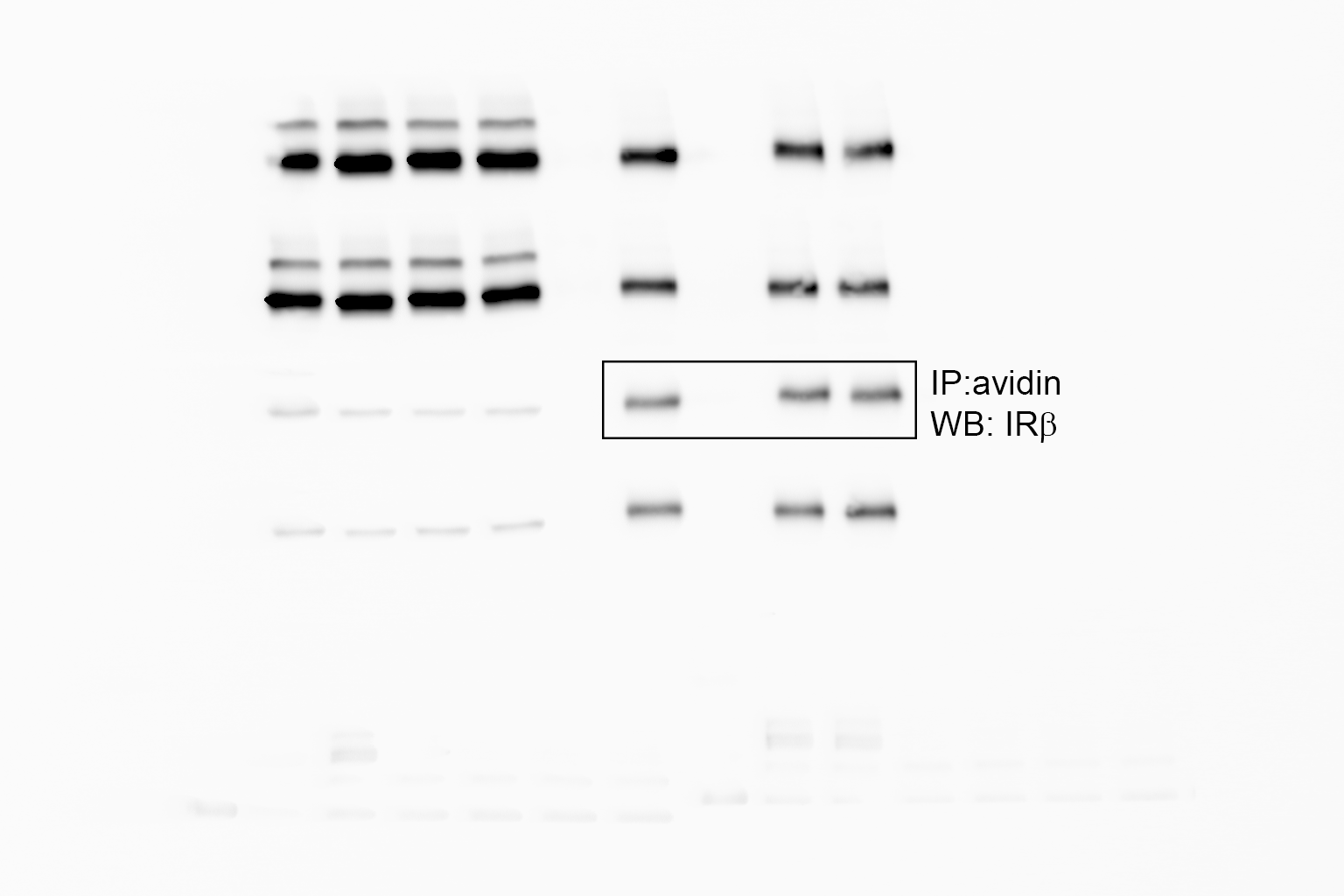

Supplement: Figure 4—figure supplement 1—source data 1. [file elife-70885-fig4-figsupp1-data1.zip › Figure 4-Figure supplement 1- source data 1/Figure 4-Figure supplement 1B/Figure 4-Figure supplement 1B_IP (IRb).tif]

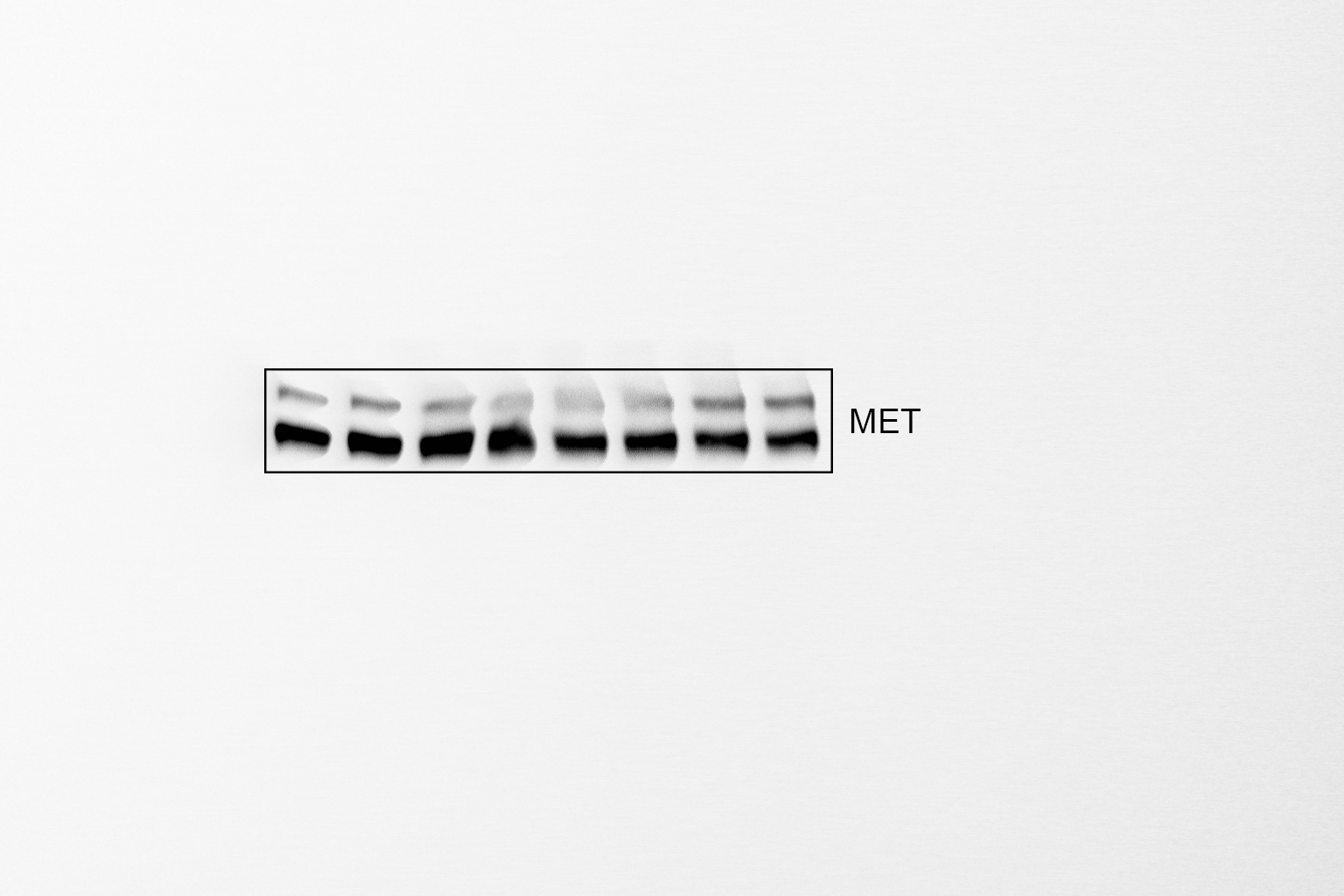

Supplement: Figure 5—source data 1. [file elife-70885-fig5-data1.zip › Figure 5-source data1/Figure 5A/Figure 5A_Input (MET).tif]

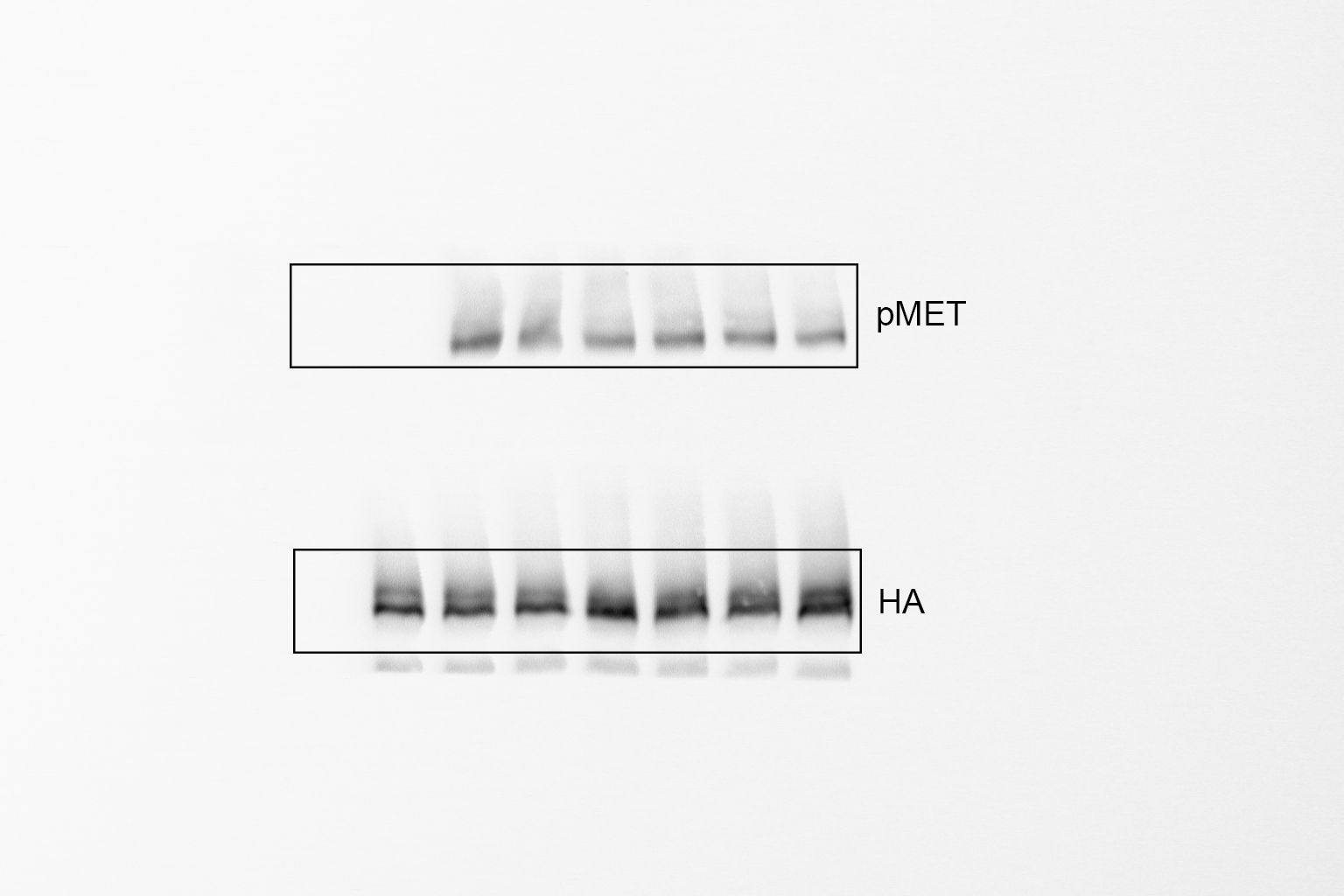

Supplement: Figure 5—source data 1. [file elife-70885-fig5-data1.zip › Figure 5-source data1/Figure 5A/Figure 5A_Input (pMET_HA).tif]

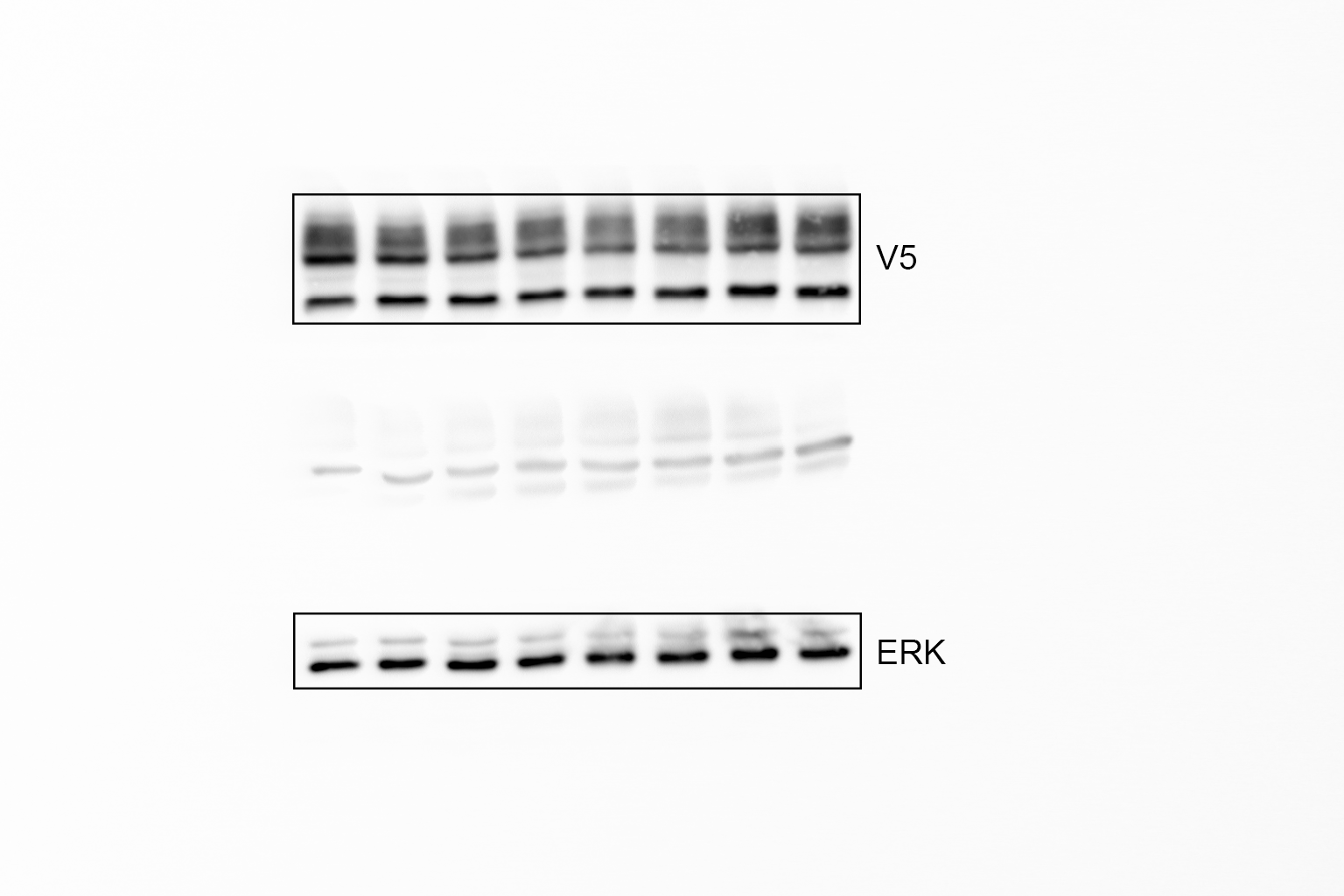

Supplement: Figure 5—source data 1. [file elife-70885-fig5-data1.zip › Figure 5-source data1/Figure 5A/Figure 5A_Input (V5_ERK).tif]

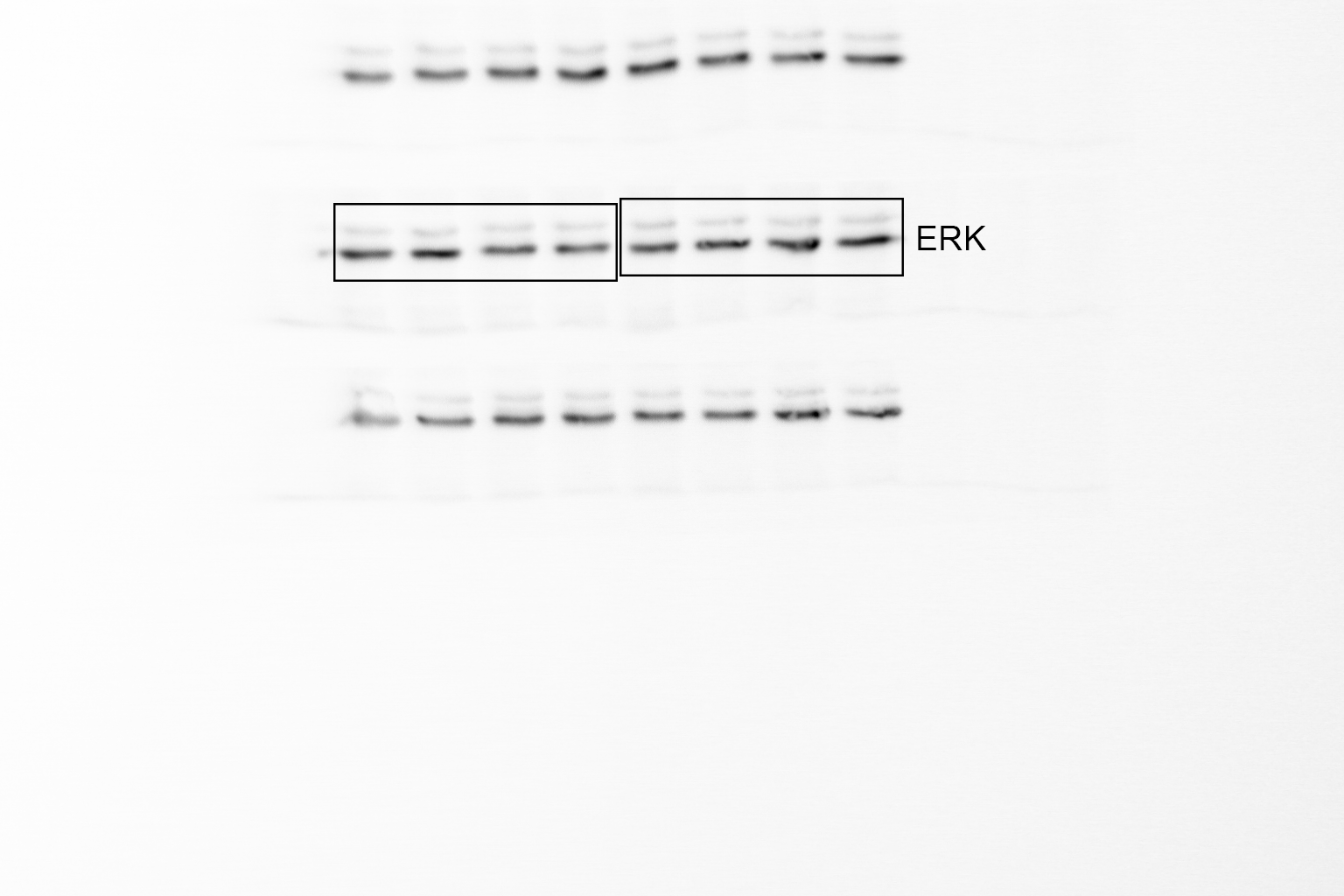

Supplement: Figure 5—source data 1. [file elife-70885-fig5-data1.zip › Figure 5-source data1/Figure 5B/Figure 5B_Input (ERK).tif]

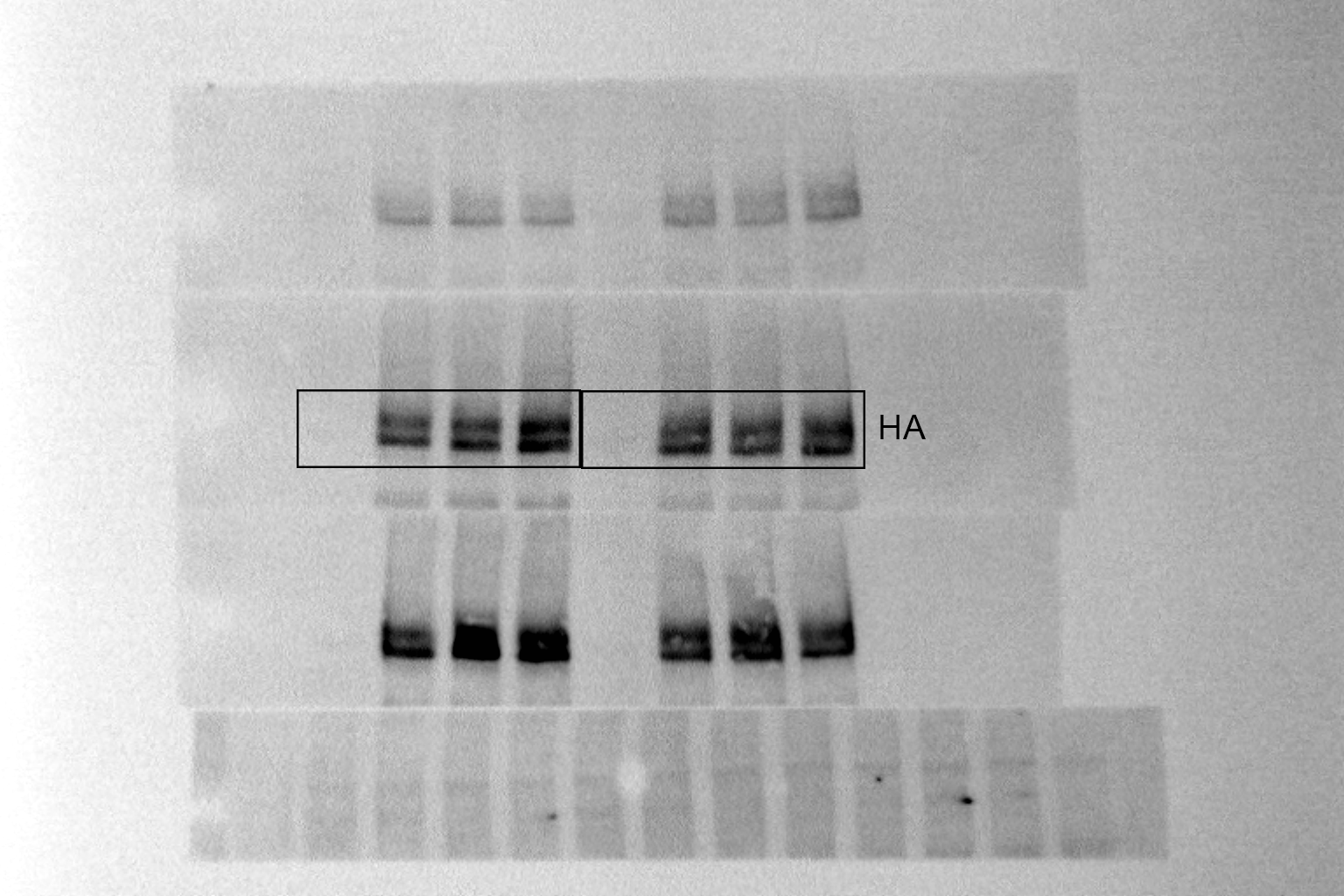

Supplement: Figure 5—source data 1. [file elife-70885-fig5-data1.zip › Figure 5-source data1/Figure 5B/Figure 5B_Input (HA).tif]

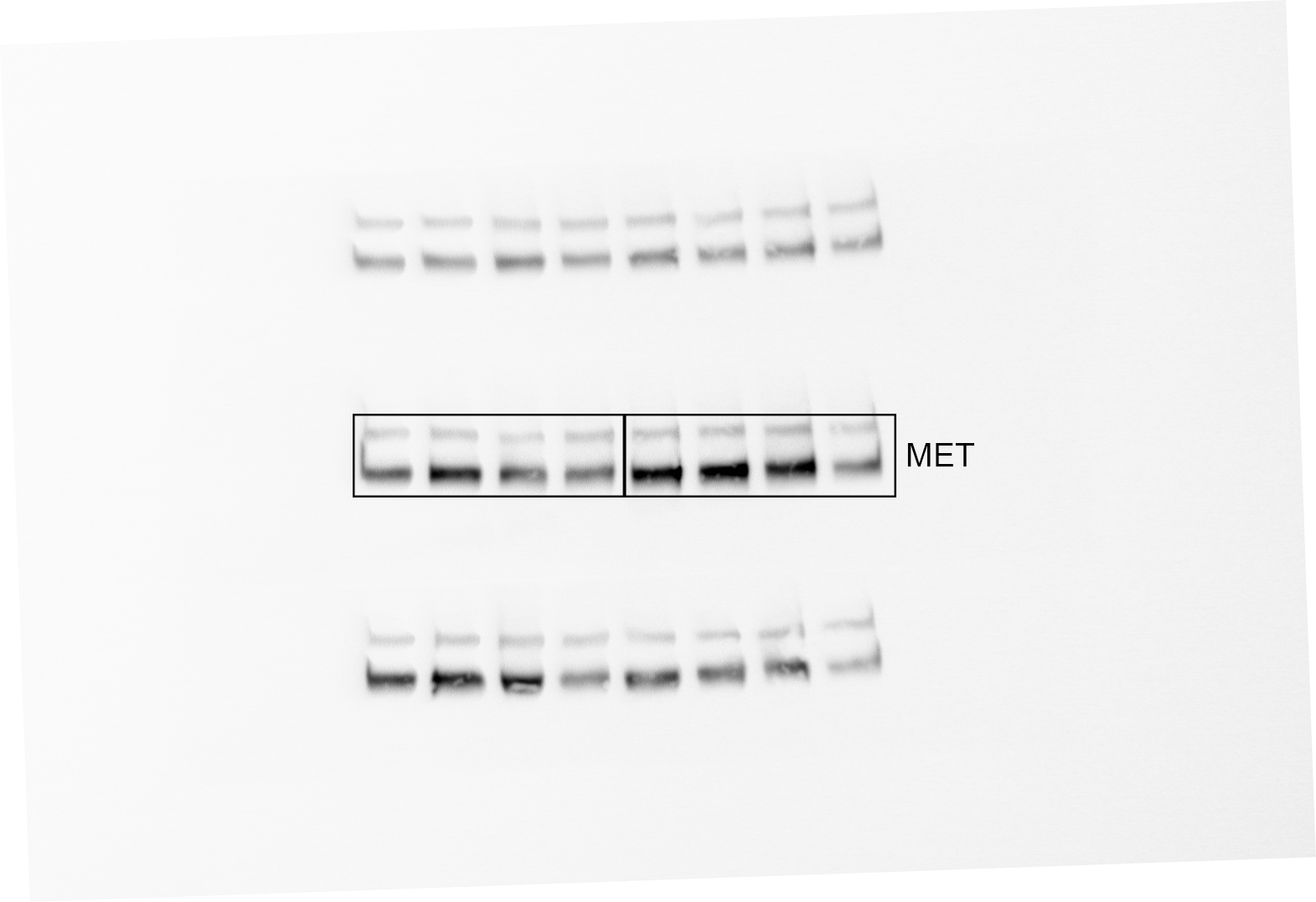

Supplement: Figure 5—source data 1. [file elife-70885-fig5-data1.zip › Figure 5-source data1/Figure 5B/Figure 5B_Input (MET).tif]

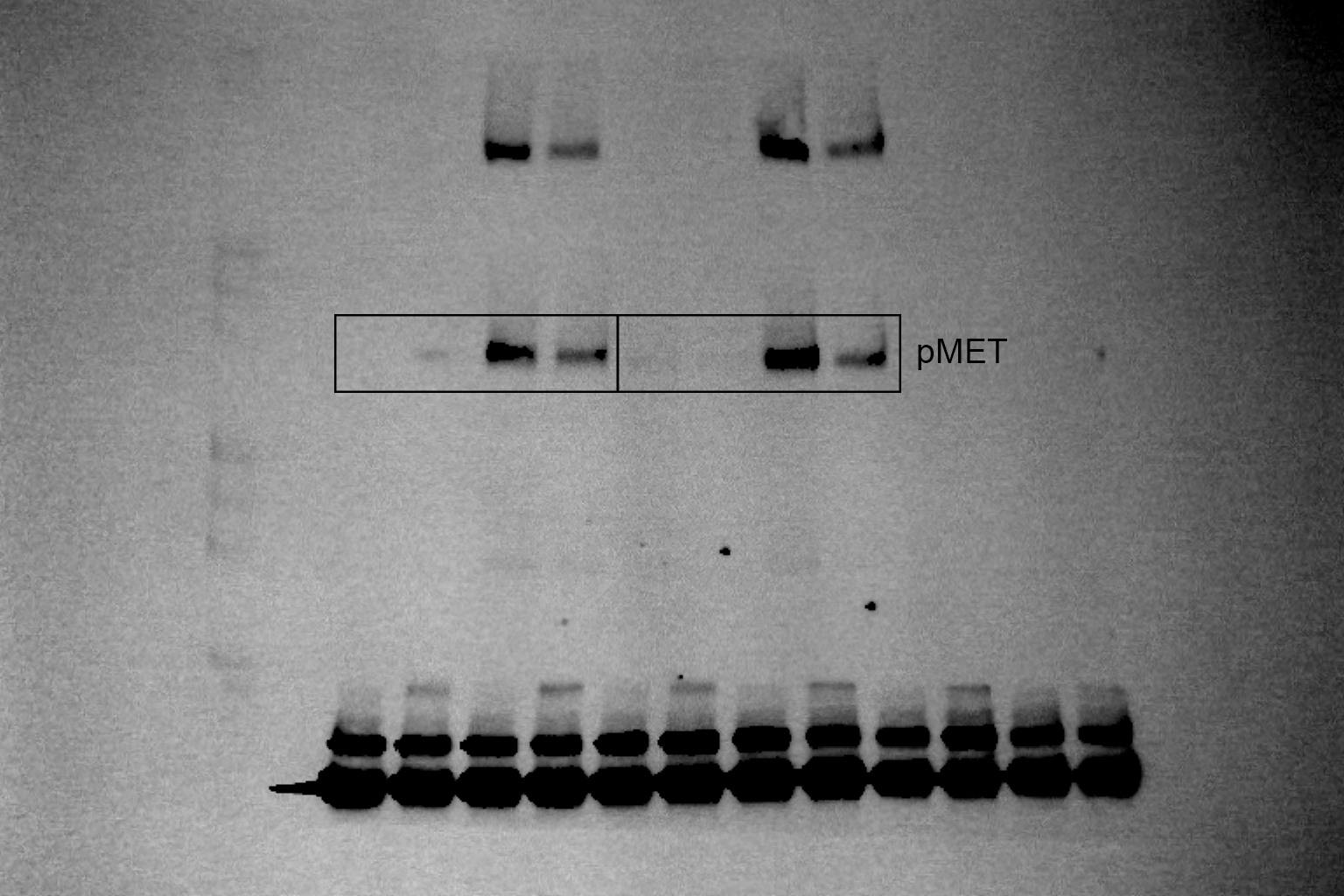

Supplement: Figure 5—source data 1. [file elife-70885-fig5-data1.zip › Figure 5-source data1/Figure 5B/Figure 5B_Input (pMET).tif]

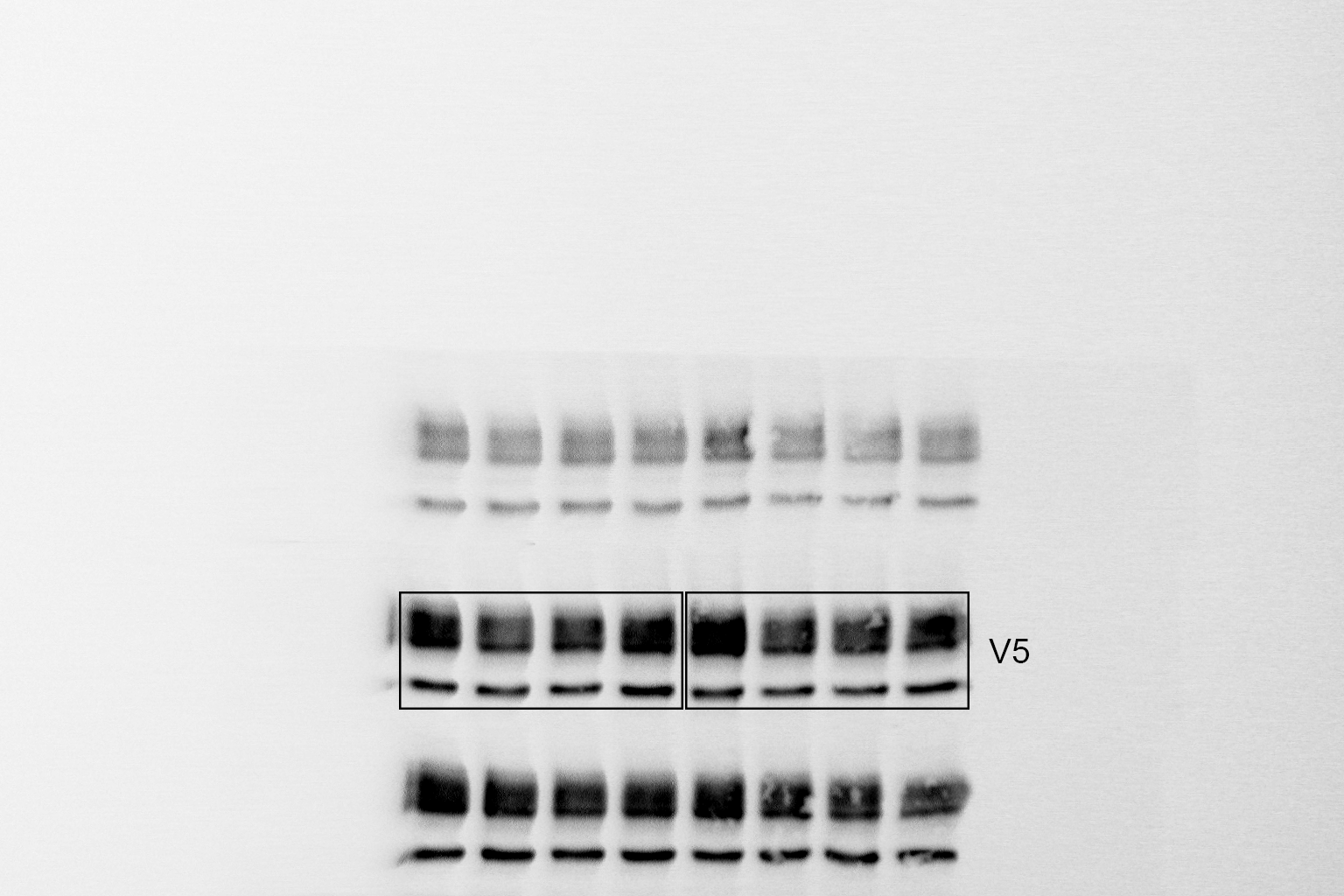

Supplement: Figure 5—source data 1. [file elife-70885-fig5-data1.zip › Figure 5-source data1/Figure 5B/Figure 5B_Input (V5).tif]

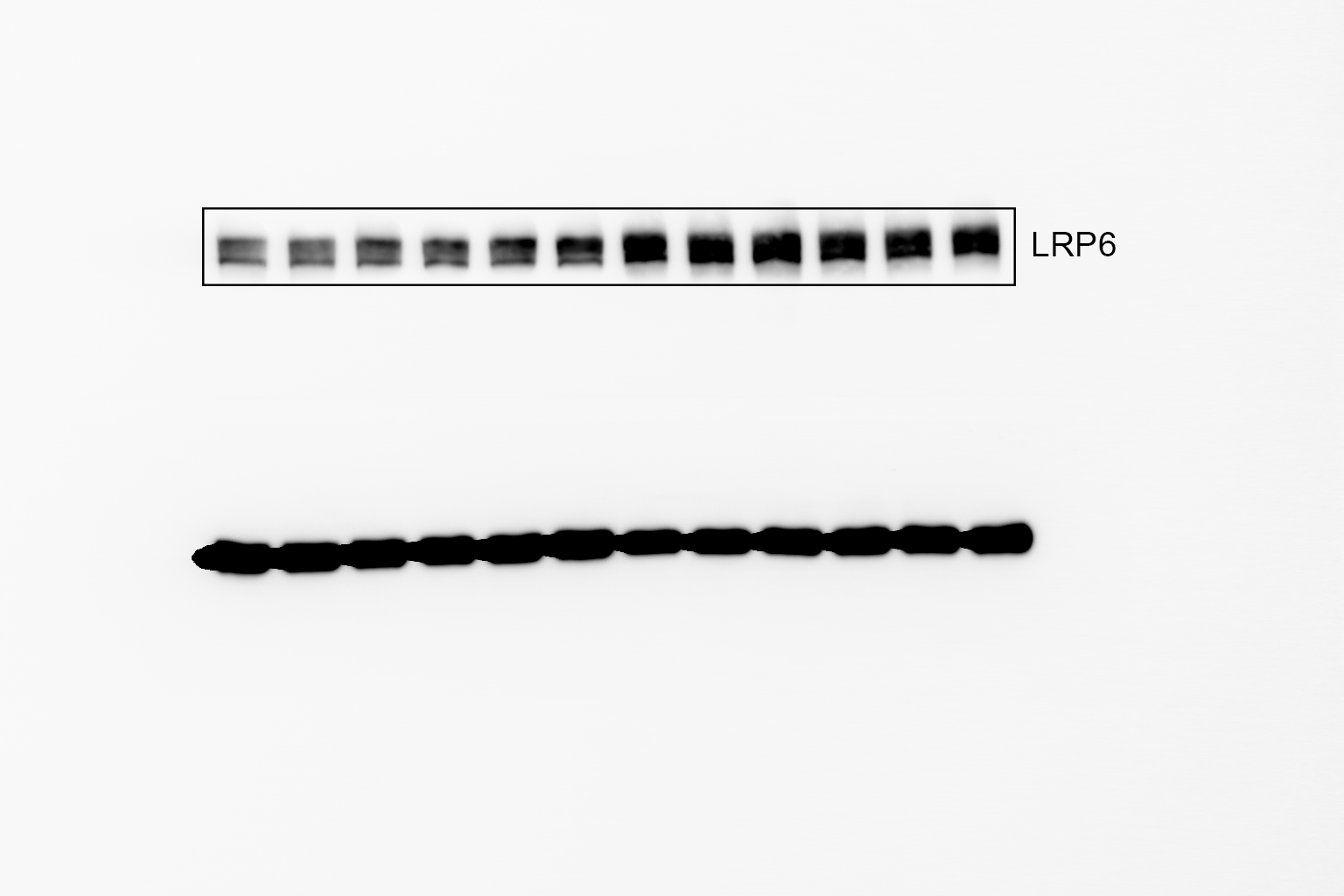

Supplement: Figure 5—source data 1. [file elife-70885-fig5-data1.zip › Figure 5-source data1/Figure 5C/Figure 5C_Input (LRP6).tif]

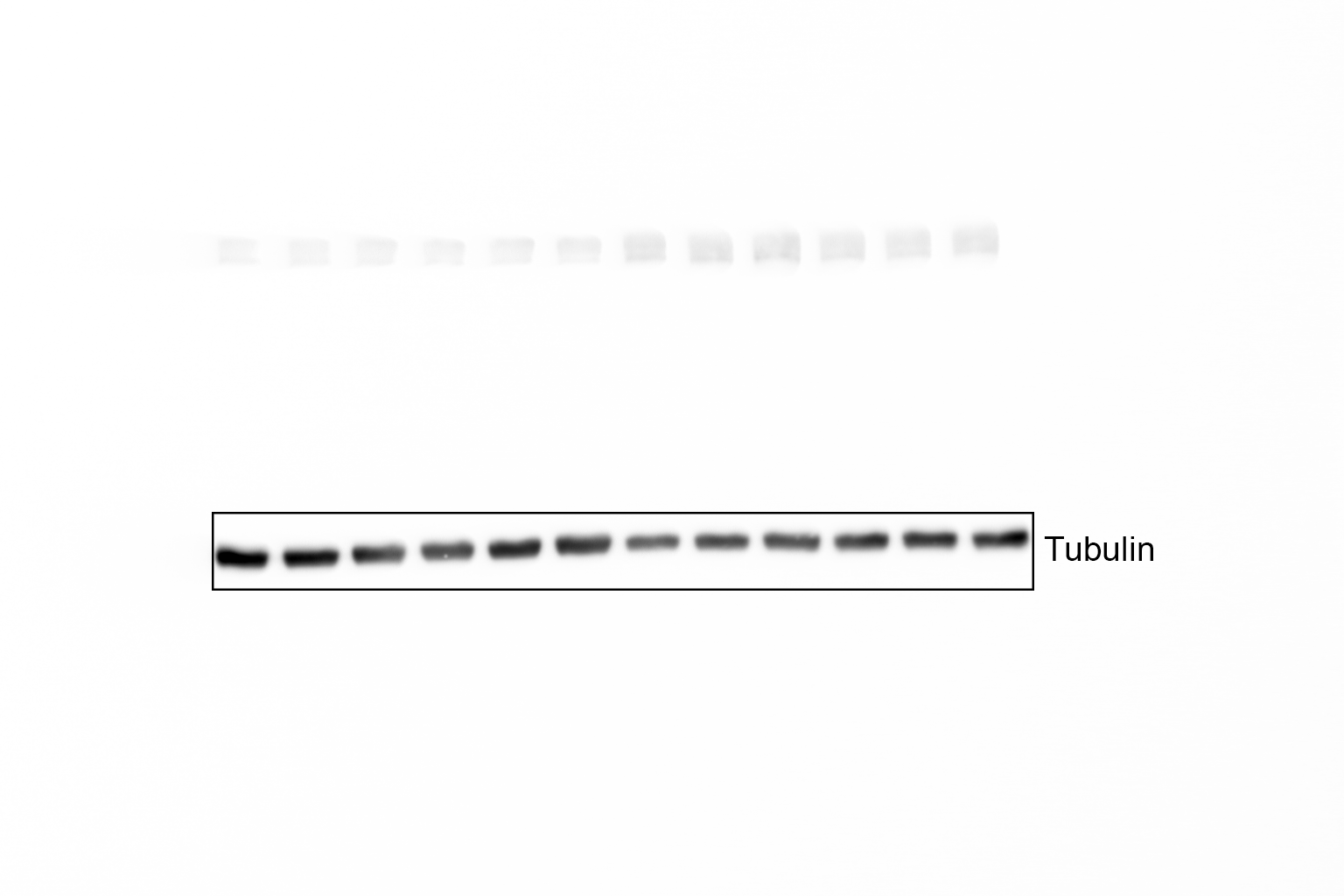

Supplement: Figure 5—source data 1. [file elife-70885-fig5-data1.zip › Figure 5-source data1/Figure 5C/Figure 5C_Input (Tubulin).tif]

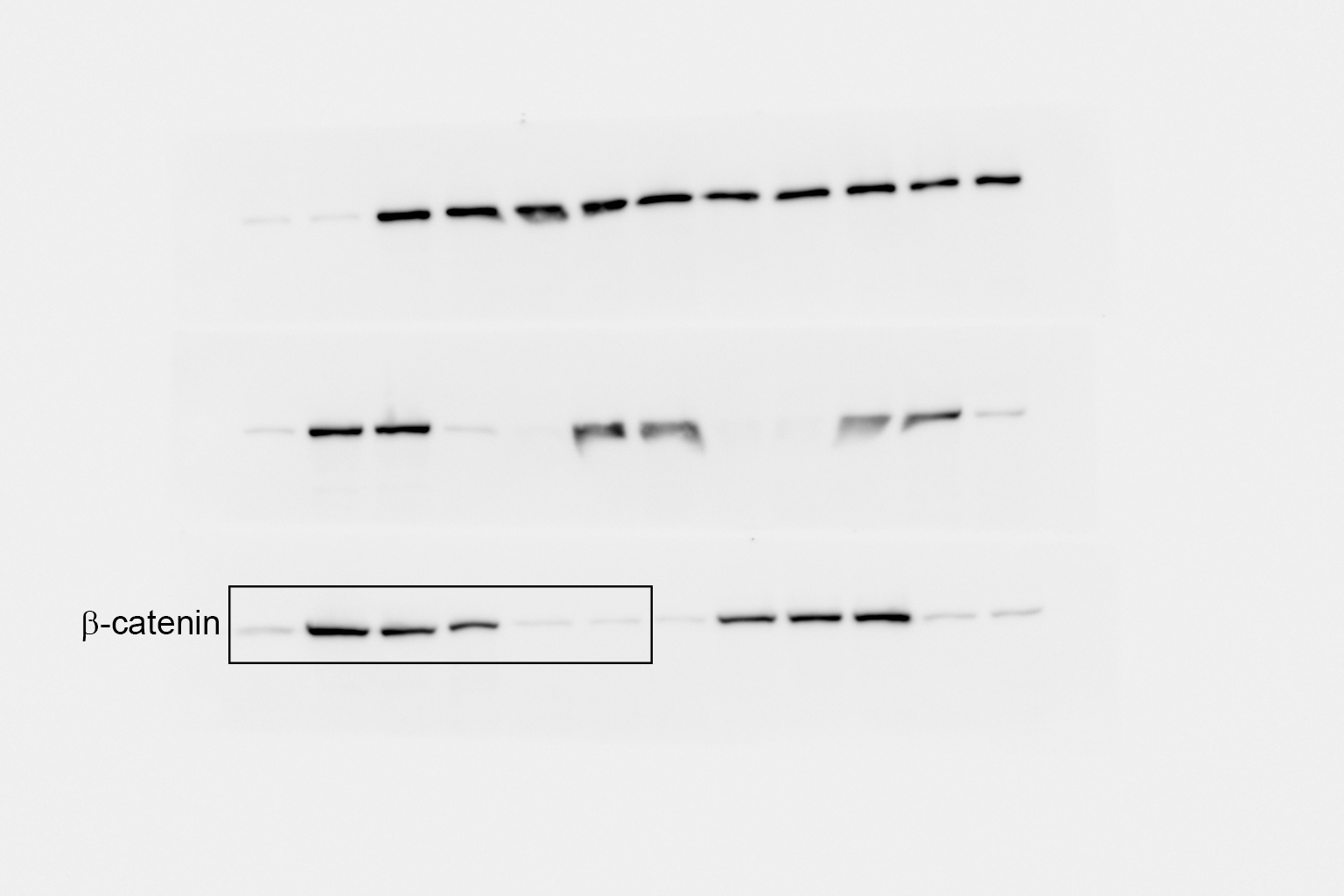

Supplement: Figure 5—source data 1. [file elife-70885-fig5-data1.zip › Figure 5-source data1/Figure 5D/Figure 5D_Input (b-cat).tif]

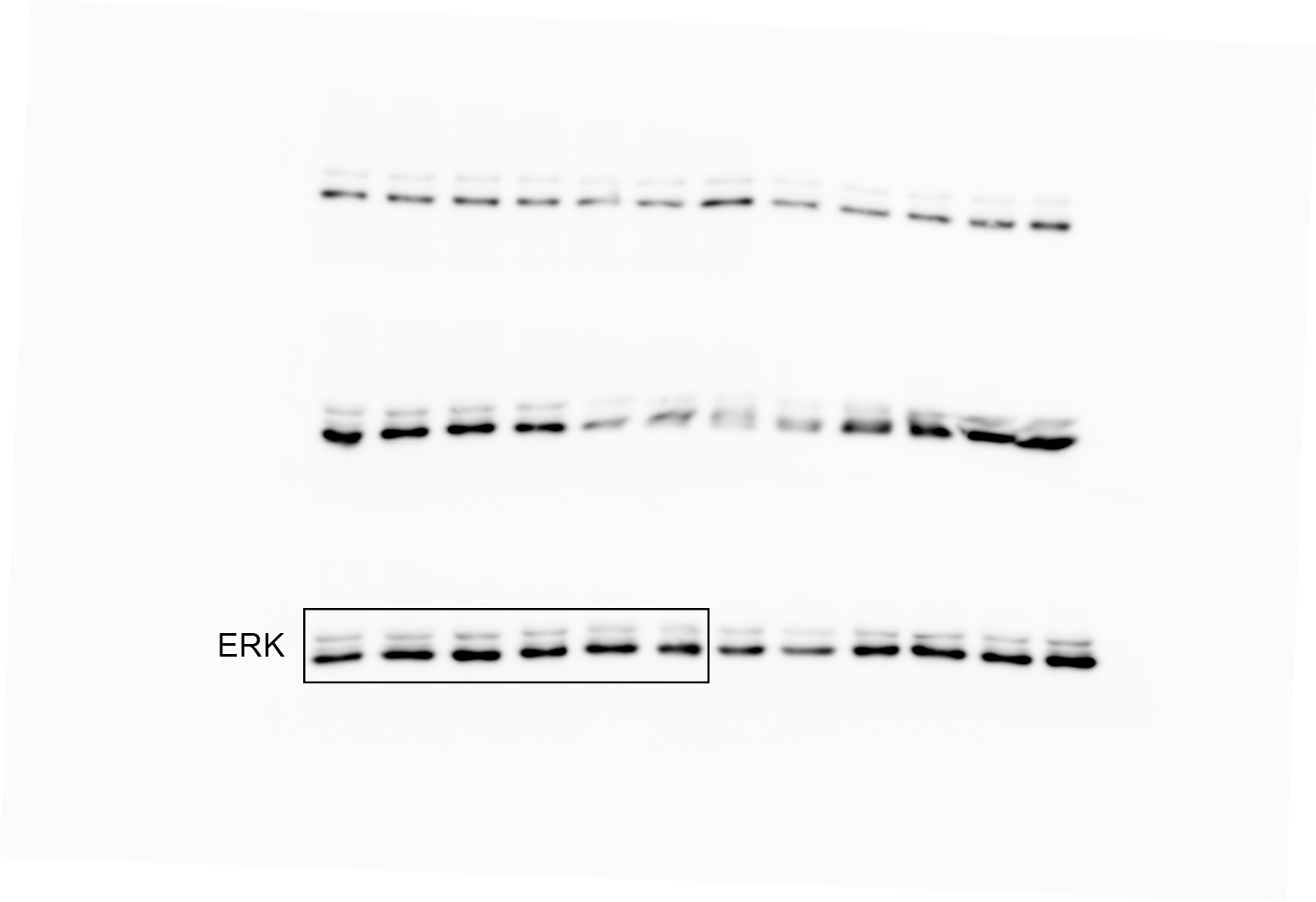

Supplement: Figure 5—source data 1. [file elife-70885-fig5-data1.zip › Figure 5-source data1/Figure 5D/Figure 5D_Input (ERK).tif]

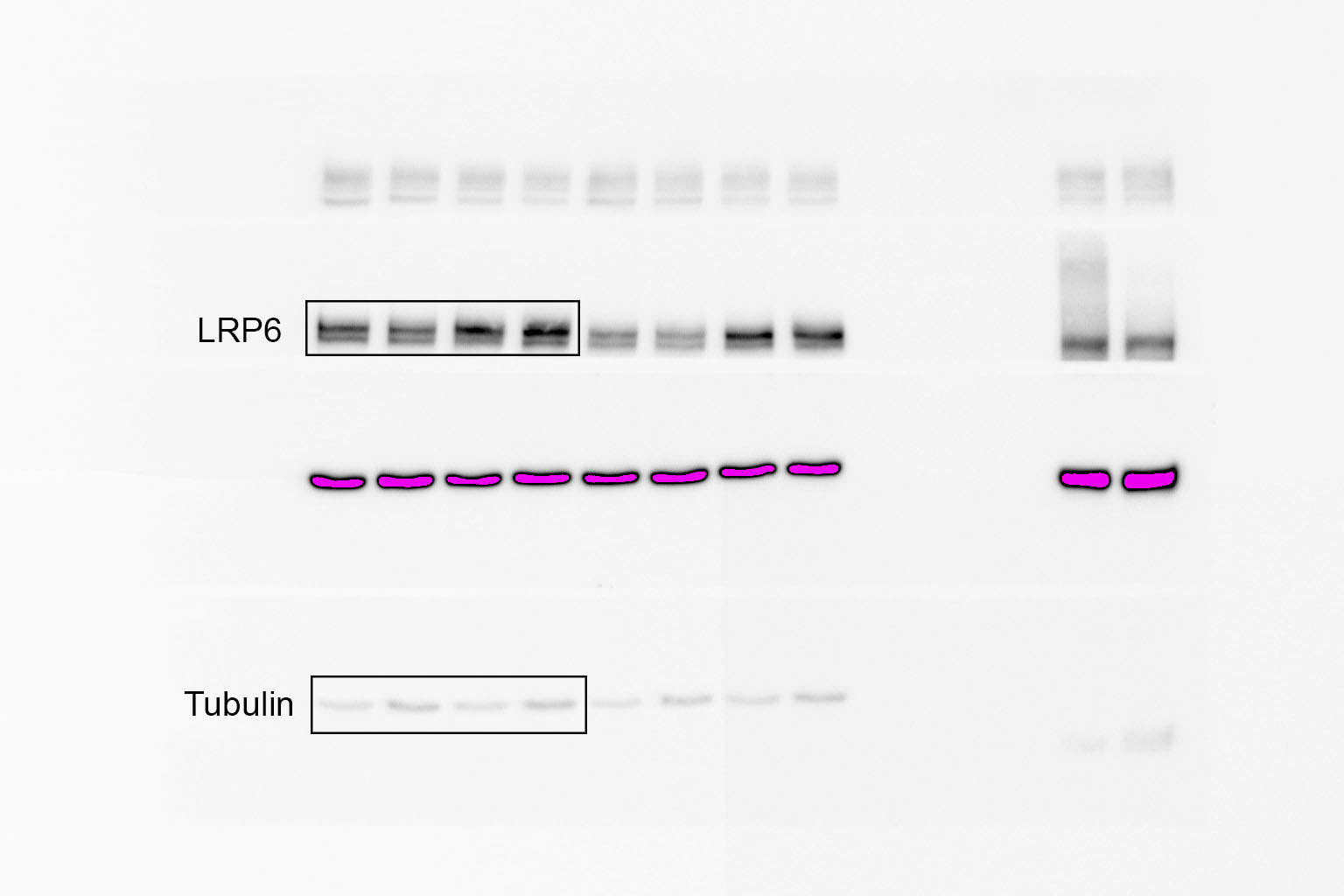

Supplement: Figure 5—figure supplement 1—source data 1. [file elife-70885-fig5-figsupp1-data1.zip › Figure 5-figure supplement 1 - source data 1/Figure 5-figure supplement 1A (LRP6_Tubulin).jpg]

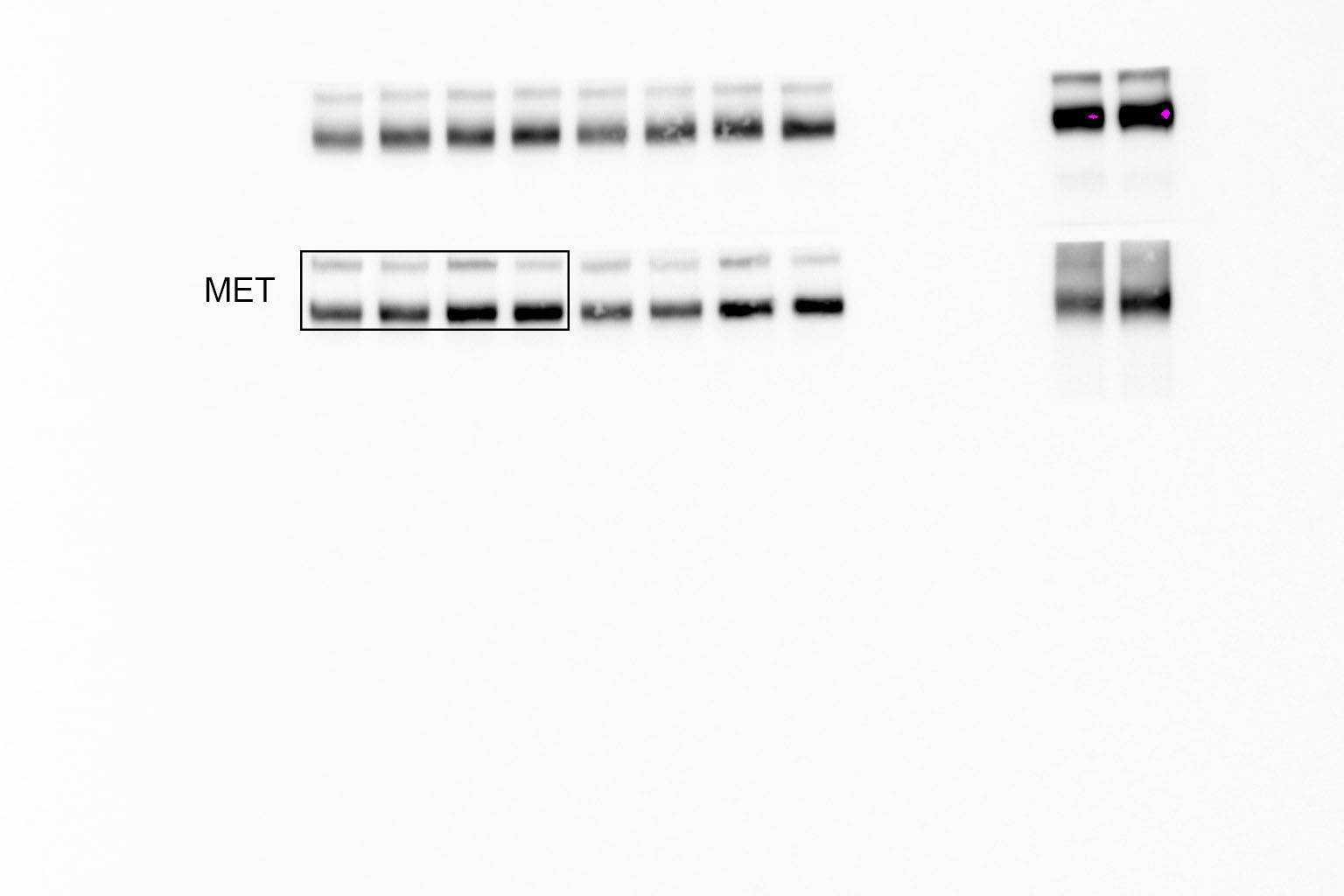

Supplement: Figure 5—figure supplement 1—source data 1. [file elife-70885-fig5-figsupp1-data1.zip › Figure 5-figure supplement 1 - source data 1/Figure 5-figure supplement 1A (MET).jpg]

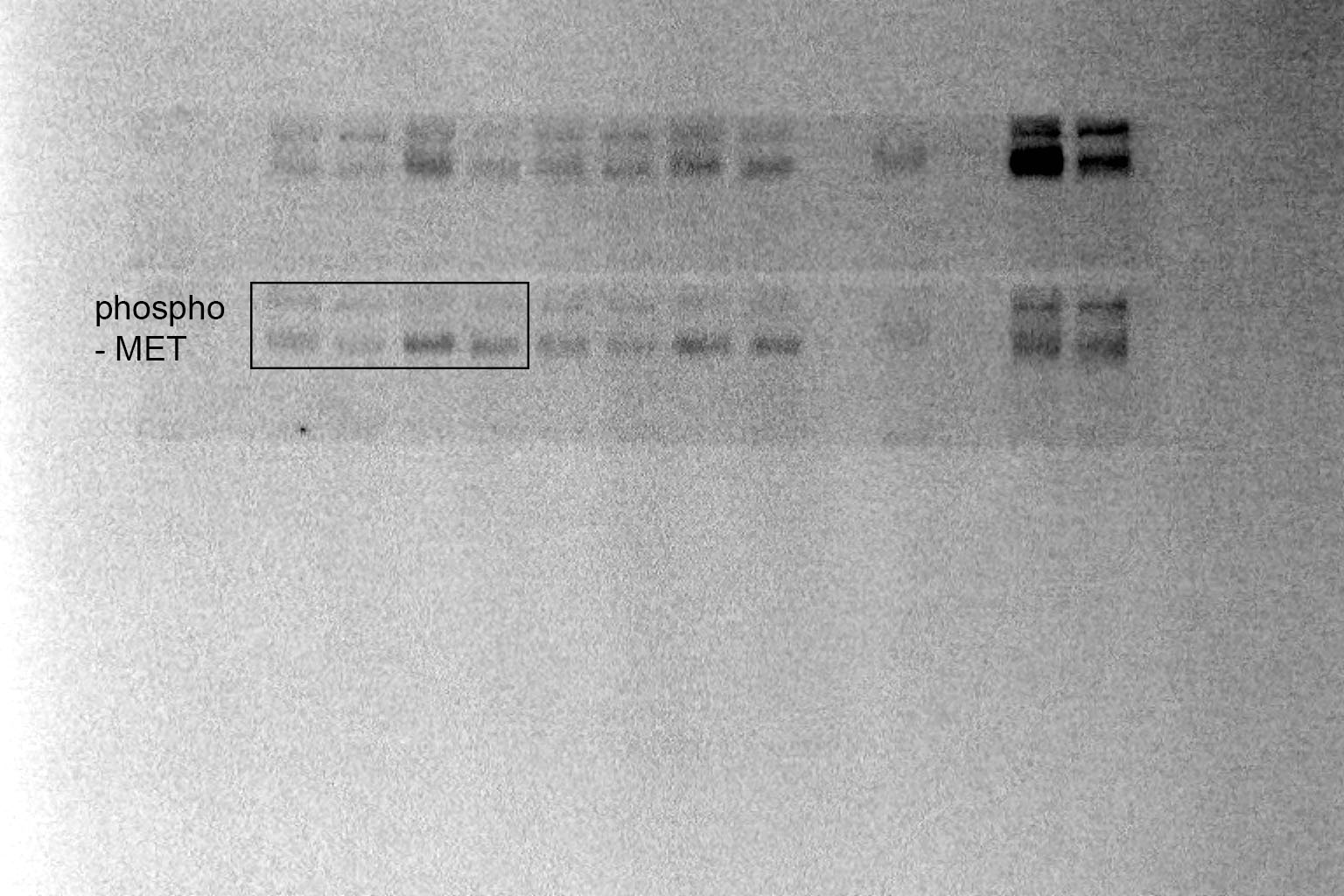

Supplement: Figure 5—figure supplement 1—source data 1. [file elife-70885-fig5-figsupp1-data1.zip › Figure 5-figure supplement 1 - source data 1/Figure 5-figure supplement 1A (phosphoMET).jpg]

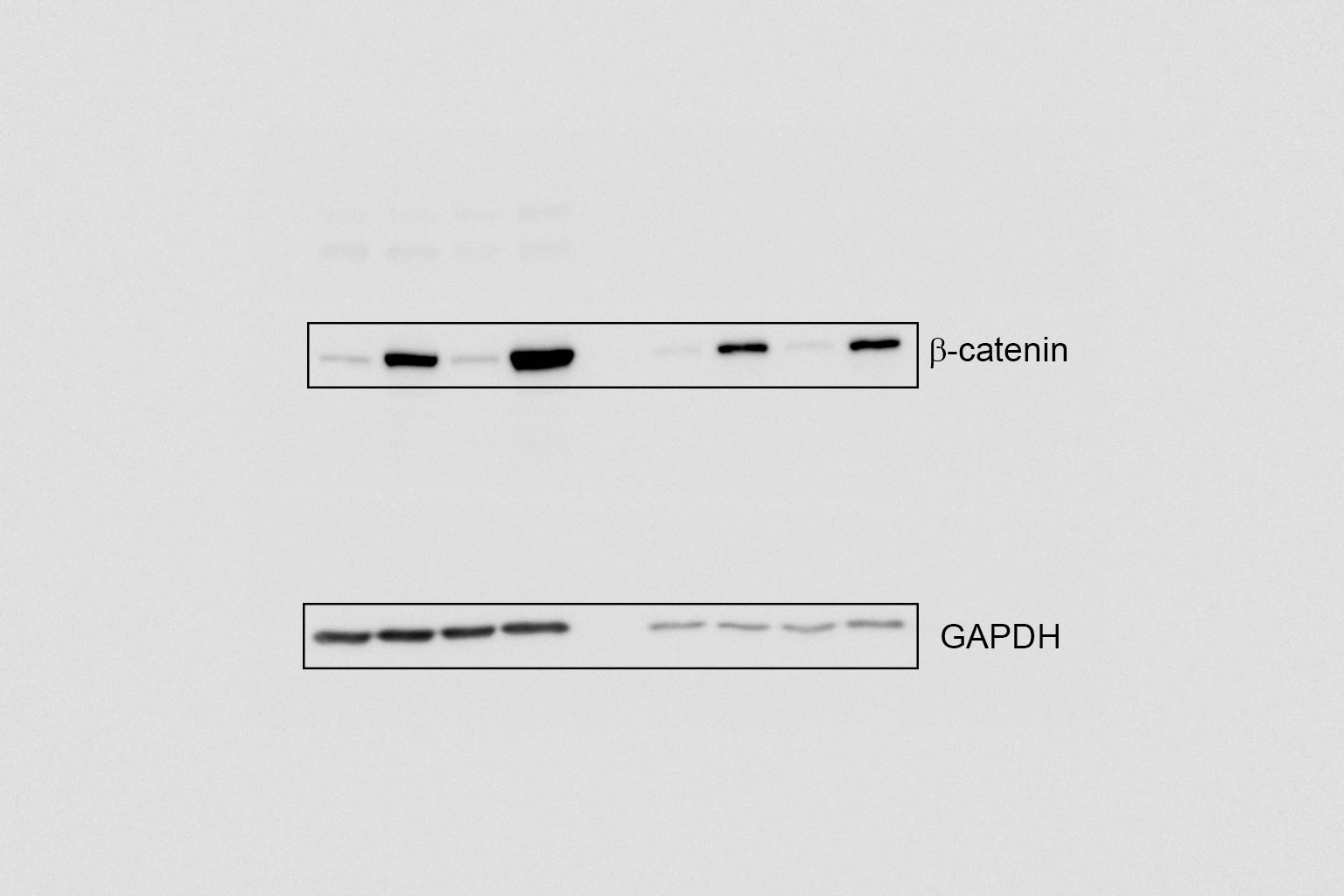

Supplement: Figure 5—figure supplement 1—source data 1. [file elife-70885-fig5-figsupp1-data1.zip › Figure 5-figure supplement 1 - source data 1/Figure 5-figure supplement 1B.jpg]

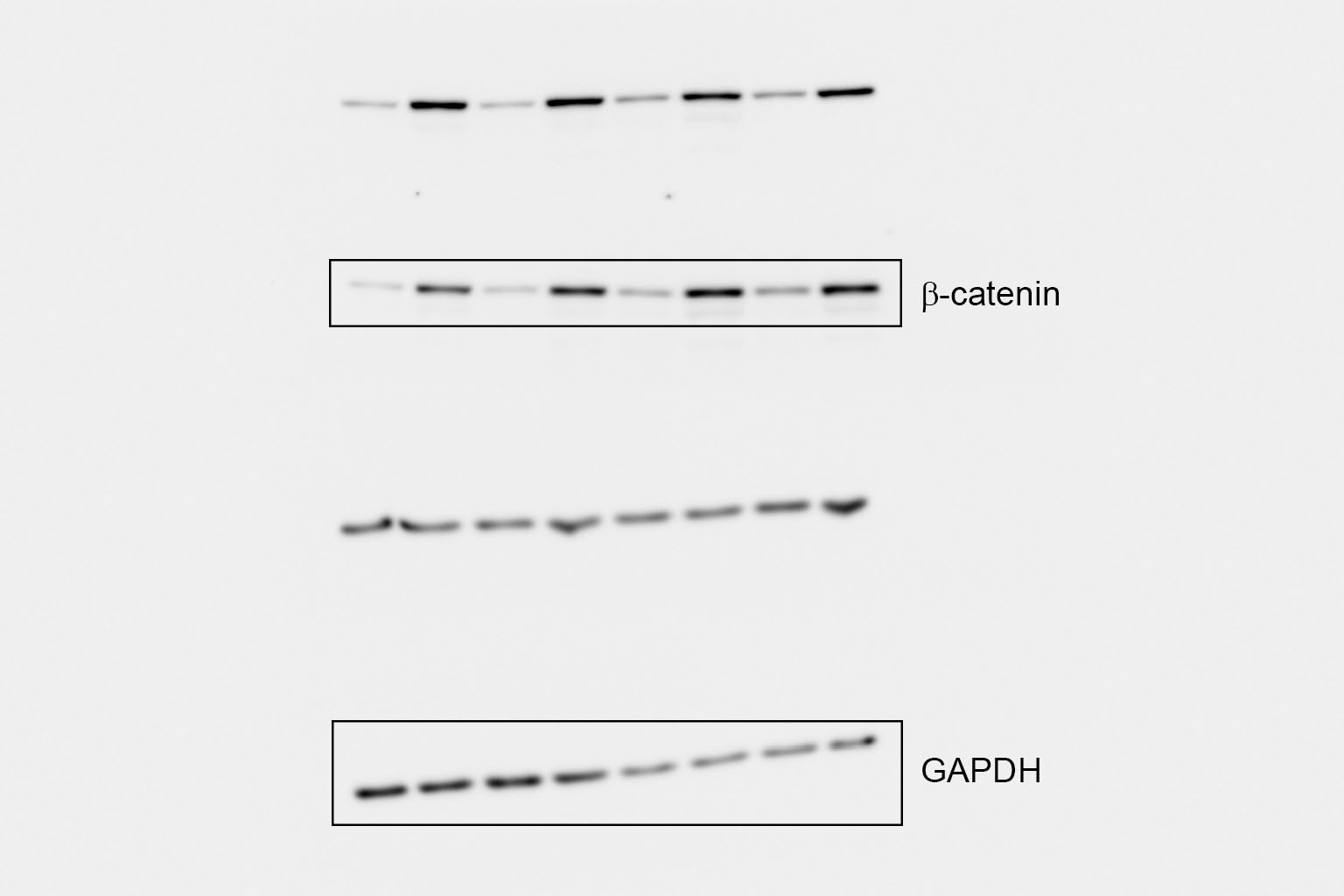

Supplement: Figure 5—figure supplement 1—source data 1. [file elife-70885-fig5-figsupp1-data1.zip › Figure 5-figure supplement 1 - source data 1/Figure 5-figure supplement 1C.jpg]

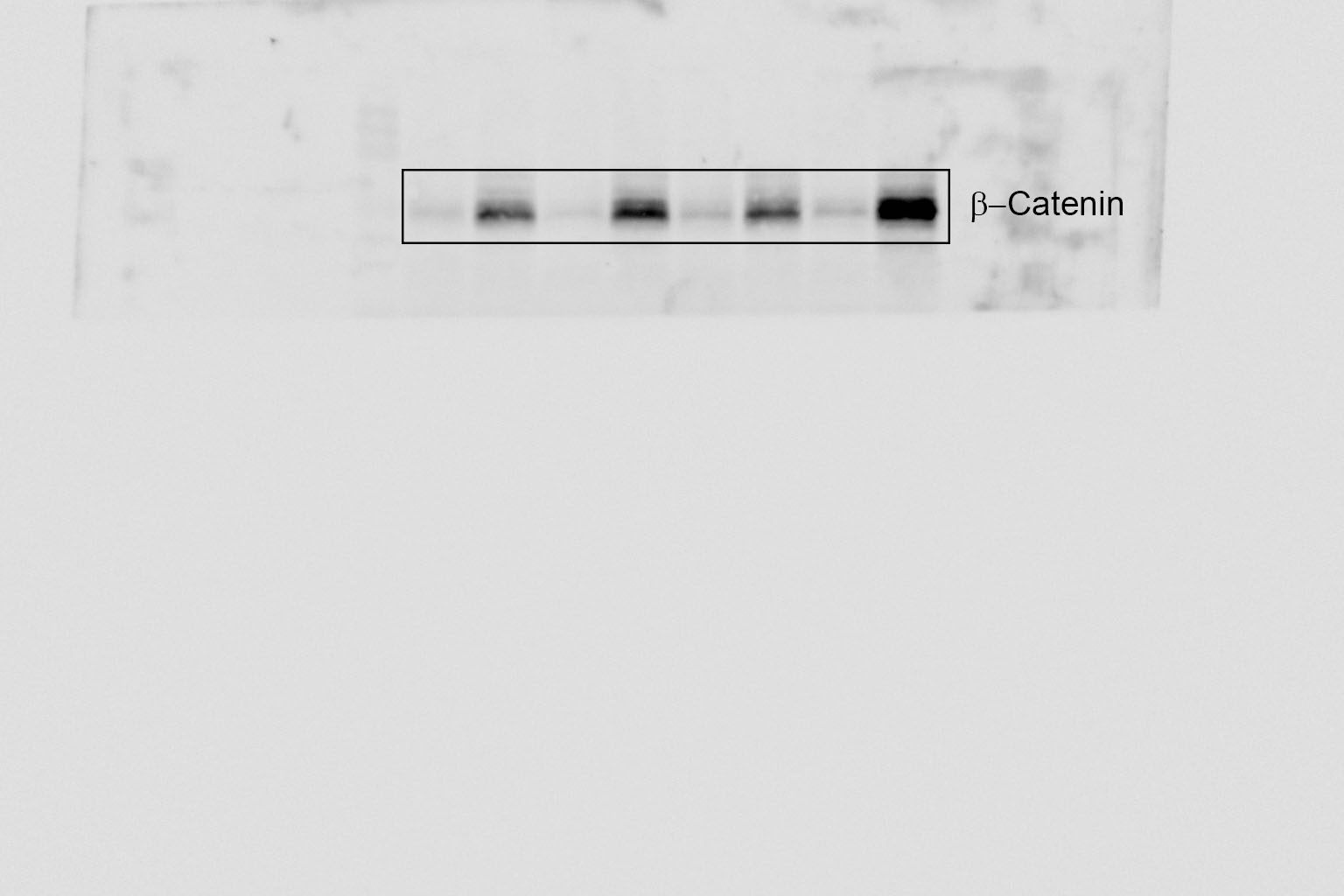

Supplement: Figure 5—figure supplement 1—source data 1. [file elife-70885-fig5-figsupp1-data1.zip › Figure 5-figure supplement 1 - source data 1/Figure 5-figure supplement 1D (bCat).jpg]

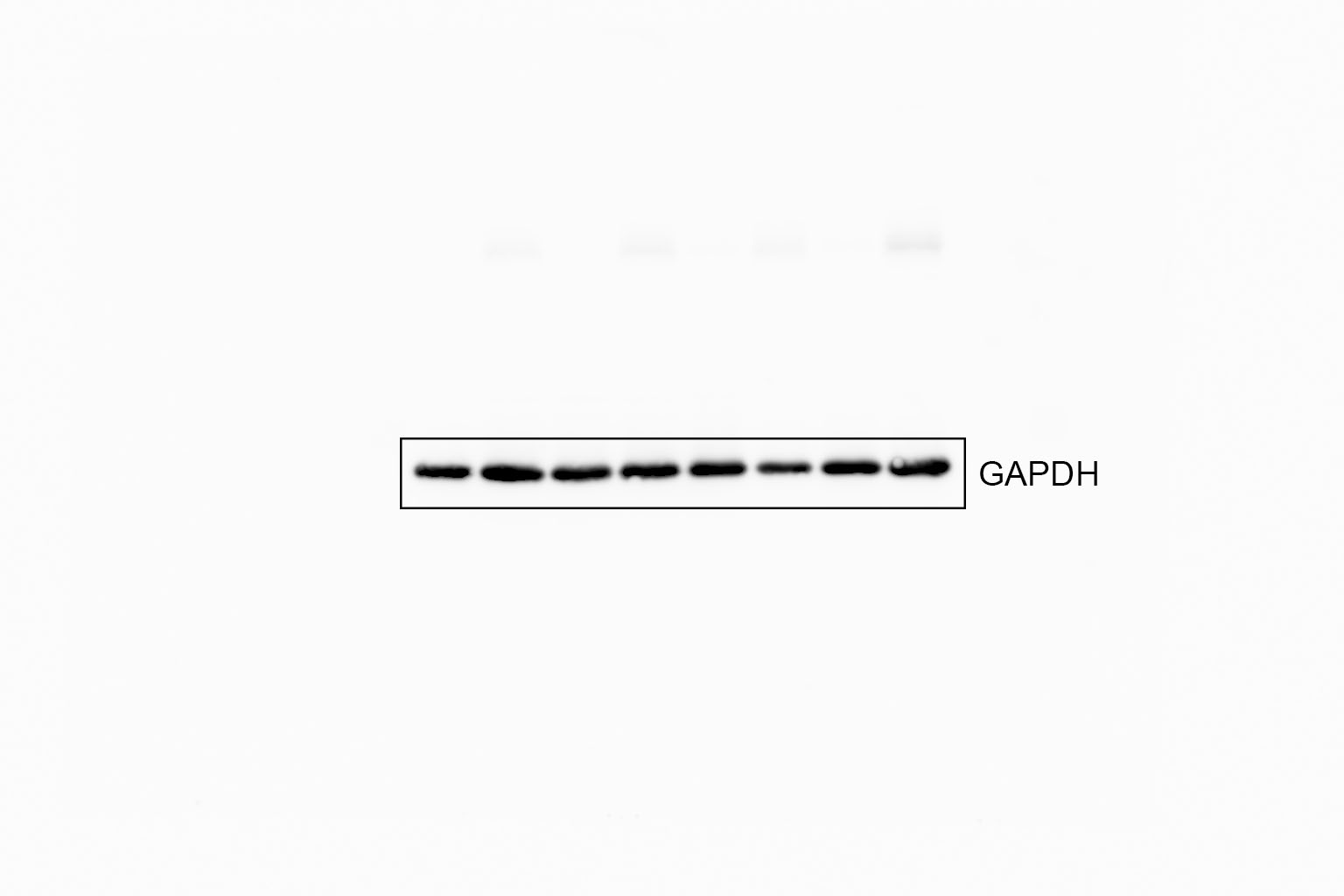

Supplement: Figure 5—figure supplement 1—source data 1. [file elife-70885-fig5-figsupp1-data1.zip › Figure 5-figure supplement 1 - source data 1/Figure 5-figure supplement 1D (GAPDH).jpg]
